# Genetically predicted physical activity levels are associated with lower colorectal cancer risk: a Mendelian randomisation study

**DOI:** 10.1038/s41416-020-01236-2

**Published:** 2021-01-29

**Authors:** Xiaomeng Zhang, Evropi Theodoratou, Xue Li, Susan M. Farrington, Philip J. Law, Peter Broderick, Marion Walker, Yann C. Klimentidis, Jessica M. B. Rees, Richard S. Houlston, Ian P. M. Tomlinson, Stephen Burgess, Harry Campbell, Malcolm G. Dunlop, Maria Timofeeva

**Affiliations:** 1 Centre for Global Health Research, Usher Institute, University of Edinburgh, Edinburgh, UK; 2 Cancer Research UK Edinburgh Centre, Medical Research Council Institute of Genetics and Molecular Medicine, University of Edinburgh, Edinburgh, UK; 3 School of Public Health and the Second Affiliated Hospital, Zhejiang University, Hangzhou, China; 4 Colon Cancer Genetics Group, Cancer Research UK Edinburgh Centre and Medical Research Council Human Genetics Unit, Medical Research Council Institute of Genetics and Molecular Medicine, University of Edinburgh, Edinburgh, UK; 5 Division of Genetics and Epidemiology, The Institute of Cancer Research, London, UK; 6 Department of Epidemiology and Biostatistics, Mel and Enid Zuckerman College of Public Health, University of Arizona, Tucson, AZ USA; 7 Edinburgh Clinical Trials Unit, Centre for Global Health Research, Usher Institute, University of Edinburgh, Edinburgh, UK; 8 MRC Biostatistics Unit, University of Cambridge, Cambridge, UK; 9 Department of Public Health, Danish Institute for Advanced Study (DIAS), University of Southern Denmark, Odense, Denmark

**Keywords:** Cancer epidemiology, Colorectal cancer, Cancer prevention

## Abstract

**Background:**

We conducted a Mendelian randomisation (MR) study to investigate whether physical activity (PA) causes a reduction of colorectal cancer risk and to understand the contributions of effects mediated through changes in body fat.

**Methods:**

Common genetic variants associated with self-reported moderate-to-vigorous PA (MVPA), acceleration vector magnitude PA (AMPA) and sedentary time were used as instrumental variables. To control for confounding effects of obesity, we included instrumental variables for body mass index (BMI), body fat percentage, waist circumference and arm, trunk and leg fat ratios. We analysed the effect of these instrumental variables in a colorectal cancer genome-wide association study comprising 31,197 cases and 61,770 controls of European ancestry by applying two-sample and multivariable MR study designs.

**Results:**

We found decreased colorectal cancer risk for genetically represented measures of MVPA and AMPA that were additional to effects mediated through genetic measures of obesity. Odds ratio and 95% confidence interval (CI) per standard deviation increase in MVPA and AMPA was 0.56 (0.31, 1.01) and 0.60 (0.41, 0.88), respectively. No association has been found between sedentary time and colorectal cancer risk. The proportion of effect mediated through BMI was 2% (95% CI: 0, 14) and 32% (95% CI: 12, 46) for MVPA and AMPA, respectively.

**Conclusion:**

These findings provide strong evidence to reinforce public health measures on preventing colorectal cancer that promote PA at a population level regardless of body fatness.

## Background

Colorectal cancer is one of the most common cancers of developed societies.^1^ Obesity is one of the risk factors for colorectal cancer.^2^ Ready access to high-calorie foodstuffs combined with a sedentary lifestyle means that obesity has become a major public health problem in developed countries, further contributing to increased colorectal cancer incidence.

Evidence from observational epidemiological studies is consistent with the premise that increased physical activity (PA) reduces colorectal cancer risk. Furthermore, the magnitude of the effect is sufficient to be meaningful for the individual.^3^ An umbrella review of 22 anatomical cancer sites concluded that there is strong evidence for a protective association between self-reported recreational PA and colorectal cancer.^4^ In addition, a recent meta-analysis of 17 cohorts and 21 case–control studies found that occupational activity, recreational activity, transport-related PA and reduced occupational sedentary behaviour were each associated with lower colorectal cancer risk.^5^ The estimated effects of increased recreational PA and occupational sedentary behaviour for colon cancer risk were 0.80 (95% confidence interval (CI): 0.71, 0.89) and 1.44 (95% CI: 1.28, 1.62), respectively, and for rectal cancer risk were 0.87 (95% CI: 0.75, 1.01) and 1.02 (95% CI: 0.82, 1.28) respectively.^5^ Despite the strength of such correlative evidence on the effect of PA on colorectal cancer risk, causality cannot be ascribed by observational studies, since the observed association could be due to confounding factors or residual confounding. Furthermore, PA is routinely measured in observational studies as a self-reported activity, and this may be systematically overestimated.^6^

Given the lack of randomised clinical trials (RCTs) to formally test the effect of a PA intervention on colorectal cancer risk, one approach is to apply Mendelian randomisation (MR) approaches to test whether the association is causal. MR explores the effect of the exposure (PA) on colorectal cancer risk through a genetic instrumental variable.^7^ Since the instrumental variable is randomly assorted at conception, it can overcome the aforementioned shortcomings such as confounding effects. Common genetic variants shown in genome-wide association studies (GWASs) to be associated with PA can be used as instrumental variables for various measures of PA. One recent MR study supports a causal association between higher PA measured by accelerometer and lower colorectal cancer risk.^8^ However, potential confounding or mediating effect of body fatness was not taken into account.^8^

As the most commonly used measure of body fatness, body mass index (BMI) is often considered as a proxy of overall body fat. The body fat percentage measured by bioimpedance is another proxy of overall body fat to compare with the results from BMI. However, for people with the same overall body fat (i.e. BMI and body fat percentage), body fat distribution changes with factors such as sex, age, ethnicity, nutritional status and fitness training level.^9^ Evidence shows that people with normal BMI but excess trunk fat are at higher risk of metabolic diseases,^10^ while those with a normal BMI but with excess leg fat are at lower myocardial infarction risk.^11^ The effect of excess body fat (BMI, body fat percentage, waist circumference and body fat distribution) on colorectal cancer risk has been well described in observational^2^ and MR studies.^12,13^

Here, using MR approaches, we have investigated the observed association between PA and colorectal cancer to establish, or refute, causality. Further, we have tested whether the effects are confounded by, or mediated through, measures of body fat. In order to comprehensively assess the influence of body fat, we employed measures of body fat including BMI, body fat percentage, waist circumference and three types of body fat distribution measured by bioimpedance.

## Methods

### Genotype data resources for colorectal cancer case–control genome-wide association analysis

We used genome-wide summary-level genotyping data imputed to a merged reference panel comprising the 1000 Genome Project and UK10K from a meta-analysis of 15 GWAS datasets^14^ from populations of European ancestry (Supplementary Method and Table S1). Briefly, the colorectal cancer GWAS meta-analysis included the following GWASs: NSCCG, the SCOT study, SOCCS/GS, SOCCS/LBC and UK Biobank GWAS, as well as ten previously published GWASs: UK1, Scotland1, VQ58, CCFR1, CCFR2, COIN, Finnish GWAS, CORSA, DACHS and Croatia. Standard quality-control measures were applied to each GWAS and summary statistic data from 31,197 cases and 61,770 controls were included in the analyses.^14^ GWAS data from the UK Biobank was excluded from the sensitivity analysis to avoid bias caused by sample overlap between exposure and outcome datasets. All studies were approved by respective ethics/institutional review committees, in accordance with the Declaration of Helsinki. Participants of all the included studies have signed the relevant consent forms.

### Generation of genetic instruments

We implemented genetic instrumental variables for three validated measures of continuous PA: self-reported moderate-to-vigorous PA (MVPA), overall acceleration vector magnitude PA (AMPA) and sedentary time. Instrumental variables for PA were extracted from two GWASs that established associations between common genetic variants (minor allele frequency ≥ 5%) and PA (Table S2): (i) a meta-analysis of GWASs of 337,234 UK Biobank participants on habitual PA;^15^ (ii) a GWAS of average acceleration vector magnitude on 91,105 UK Biobank participants.^16^ All summary-level statistics from the GWASs used in the current work were restricted to CEU (northern and western European) populations. Therefore, population stratification is not a potential bias in our study. Data for the MVPA measure were derived from self-reported questionnaires completed as part of the UK Biobank dataset collected between 2006 and 2010.^17^ AMPA and sedentary time were collected from a subset of 91,105 UK Biobank participants wearing an accelerometer 7 days between 2013 and 2015.^18^ Details of MVPA, AMPA and sedentary time were described in the Supplementary Methods. Klimentidis et al.^15^ detected eight single-nucleotide polymorphism (SNPs) for MVPA at *P* < 5 × 10^−9^ (Table 1). Doherty et al.^16^ detected three SNPs at *P* < 5 × 10^−9^ and five SNPs at *P* < 5 × 10^−8^ for AMPA and six SNPs for the sedentary time at *P* < 5 × 10^−8^ (Table 1). The SNP-based heritability estimate was 5% for MVPA, 21% for AMPA and 12.9% for sedentary time.^15,16^

**Table 1:** Description of instrumental variable (IV) for each measure.

Exposures	No. of SNPs for the IV	Variance explained by the IV (*R*^2^)^a^	Sample size of exposure GWAS	Population	Reference (PMID)
MVPA	7	0.0007	385,790	European	Klimentidis et al.^15^ (29899525)
AMPA	5	0.002	91,105	European	Doherty et al.^16^ (30531941)
AMPA^b^	3	0.001	91,105	European	Doherty et al.^16^ (30531941)
Sedentary time	6	0.001	91,105	European	Doherty et al.^16^ (30531941)
BMI	68	0.0232	322,154	European	Locke et al.^60^ (25673413)
Body fat percentage	370	0.053	331,117	European	Bycroft et al.^61^ (30305743)
Waist circumference	59	0.010	224,459	European	Shungin et al.^62^ (25673412)
AFR	15	0.002	362,499	European	Rask-Andersen et al.^11^ (30664634)
TFR	50	0.002	362,499	European	Rask-Andersen et al.^11^ (30664634)
LFR	46	0.002	362,499	European	Rask-Andersen et al.^11^ (30664634)

*GWAS* genome-wide association study, *MVPA* self-reported moderate-to-vigorous physical activity, *AMPA* acceleration vector magnitude physical activity, *SNP* single-nucleotide polymorphism, *IV* instrumental variable, *BMI* body mass index, *AFR* arm fat ratio, *LFR* leg fat ratio, *TFR* trunk fat ratio.

^a^*R*^2^ was estimated base on the formula: $2 \times {\mathrm{EAF}}\times (1-{\mathrm{EAF}})\times \beta^{2}$.

^b^Threshold at *P* < 5 × 10^−9^.

**Fig. 1:**
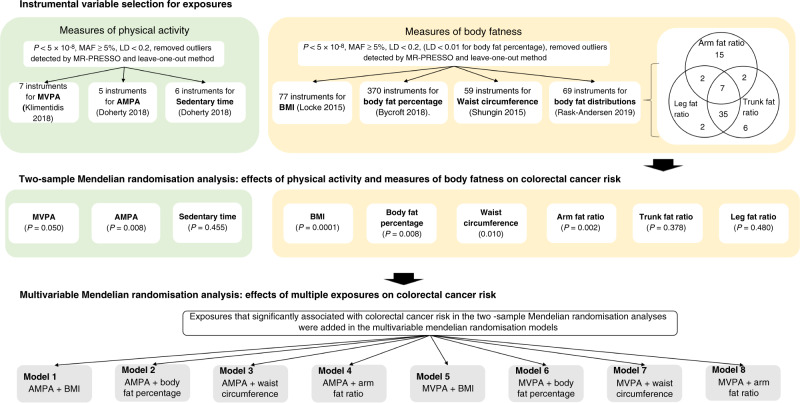
Schematic representation of the study design. MAF minor allele frequency, LD linkage disequilibrium, MVPA self-reported moderate-to-vigorous physical activity, AMPA acceleration vector magnitude physical activity, BMI body mass index.

Several genetic variants associated with MVPA, AMPA and sedentary time were previously found to be also associated with the weight, BMI and arm/body/leg/trunk fat percentage (Table S3). We included BMI, body fat percentage, waist circumference and body fat distribution as indicators of obesity in our analysis. The body fat distribution consists of arm fat ratio (AFR), trunk fat ratio (TFR) and leg fat ratio (LFR) measured by segmental bio-electrical impedance (sBIA). The information about  instrumental variables for measures of body fatness can be found in Supplementary Method, Table 1 and Fig. 1. The threshold of linkage disequilibrium (LD) was set as *R*^2^ > 0.2. Due to the limited variance explained by the MVPA SNPs, and the fact that the inverse variance-weighted, MR-Egger and MR-Robust methods can incorporate correlation between variants, we included all the SNPs to generate instrumental variable and added correlation matrix into these analyses (available in R package ‘MendelianRandomization’). We excluded rs149943 from the median-based method because of LD (*r*^2^ = 0.35) with rs3094622.

### Two-sample MR

Having determined the effect estimates of SNPs on PA and each measure of body fat from GWASs (Table 1), we tested the effects of these SNPs on colorectal cancer risk using genome-wide data from our previous colorectal cancer GWAS meta-analysis.^14^ The causal effects and the corresponding standard errors of exposures on colorectal cancer were calculated by using the random-effect inverse variance-weighted method.^19^ We then evaluated the heterogeneity among the causal effects of each variant (Cochran’s *Q* statistic). *P* values < 0.10 were considered indicative of significant heterogeneity.^20^

### Multivariable MR

We applied multivariable MR^21^ to elucidate the causal relationship between PA and colorectal cancer while keeping measures of body fatness constant. First, we analysed the beta–beta correlations of SNP effects for each exposure through Pearson’s correlation coefficient analysis, including significant SNPs of all measures of PA and body fatness. Next, we ran multivariable MR for measures of PA that significantly associated with colorectal cancer risk in the two-sample MR by adding measures of body fatness that significantly associated with colorectal cancer risk in the two-sample MR as covariates. In total, eight models were fitted (Fig. 1). All SNPs associated with each trait were included to generate instrumental variables for each model (Fig. 1). The pairwise LD threshold between all of these SNPs was set at *R*^2^ > 0.2.

The rationale of the study design is shown in Fig. 1.

### Sensitivity analysis

We applied a variety of sensitivity analyses testing different MR assumptions.^22^ Specifically, we performed MR-Pleiotropy Residual Sum and Outlier (MR-PRESSO),^23^ MR-Robust,^24^ MR-Egger,^25^ leave-one-out method,^26^ mode-based estimate^27^ and the median-based method.^28^ MR-Robust applies MM-estimation (modified maximum-likelihood estimation) with Tukey’s bisquare function, which efficiently limits the contribution of outliers.^24^ MR-Egger was applied to explore any potential bias introduced by pleiotropy. In particular, when the intercept of MR-Egger differs from zero (at *p* < 0.05), then either directional pleiotropy is indicated or the InSIDE assumption is violated.^25^ We also applied the mode-based estimate, which works well when most estimates of identical individual-instrument causal effects are derived from valid instrumental variables and the weighted median-based method, which allows for 50% of invalid weights.^27,28^

MR-PRESSO was applied to identify horizontal pleiotropic outliers.^23^ When both MR-PRESSO and leave-one-out method indicated an outlier, we took the analysis after removing the outlier as our main analysis. Two-sample MR estimates can be biased when samples between exposure and outcome GWASs overlapped.^29^ To minimise the risk of this bias, we performed sensitivity analysis after excluding UK Biobank cohort participants from colorectal cancer GWAS meta-analysis. In addition, we checked the GWAS Catalogue^30,31^ (https://www.ebi.ac.uk/gwas/home, accessed on 2 February 2020) and PhenoScanner^32,33^ (http://www.phenoscanner.medschl.cam.ac.uk/, accessed on 2 February 2020) to determine whether the PA instrumental variables were associated with other traits, consistent with pleiotropic effects. For all MR analyses, the *P* value threshold was set at 0.05. All statistical analyses were performed on R v3.6.1 with packages ‘MendelianRandomization’ and ‘TwoSampleMR’.^26,34^

### Power calculation

The non-centrality parameter-based approach was applied to estimate the power of this study.^35^ The *R*^2^ (the variance explained by each genetic instrument) was estimated by the following formula: $2 \times {\mathrm{EAF}} \times \left( {1 - {\mathrm{EAF}}} \right) \times \beta ^2$ and *F*-statistic $F = R^2 \times (N - 2)/(1 - R^2)$ was applied to estimate the strength of genetic instrument,^36,37^ where EAF is effect allele frequency, beta is the effect size of instrumental variables per standard deviation (SD) change of PA and *N* is the sample size of PA GWAS. The MVPA-, AMPA- and sedentary time-related variants explained ~0.07%, 0.2% and 0.1% of the phenotypic variance, respectively. We fixed the type I error at *α* < 0.05 and listed the effect estimates that could be detected for each SD increase of the PA time. We required 80% power to detect any effects. Effect sizes that can be detected with the power of 0.8, as well as *F*-statistics for instrumental variables are presented in Table S4. The *F*-statistic for all the analyses did not indicate weak instruments (*F* > 10).

## Results

### Two-sample MR

**Fig. 2:**
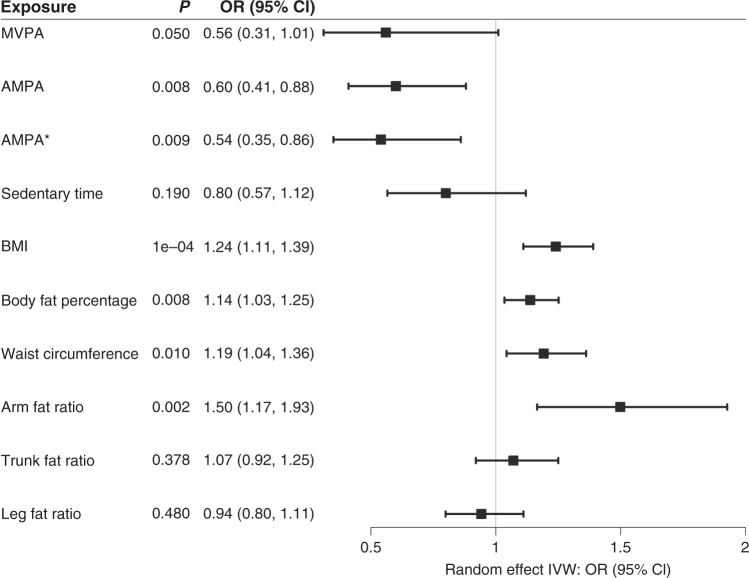
Forest plot of two-sample Mendelian randomisation studies exploring associations between exposures to colorectal cancer risk. MVPA self-reported moderate-to-vigorous physical activity, AMPA acceleration vector magnitude physical activity at *P* < 5 × 10^−8^, AMPA* acceleration vector magnitude physical activity at *P* < 5 × 10^−9^, BMI body mass index, OR odds ratio, CI confidence interval, IVW inverse variant-weighted method, *P* *P* value for random effect IVW result.

The MR result indicated a decreased colorectal cancer risk through the effect of MVPA. The odds ratio (OR) for inverse variance-weighted MR was 0.56 for colorectal cancer risk per 1 SD increase of MVPA (95% CI: 0.31, 1.01) (Figs. 2 and S1) and each sensitivity analysis method generated similar effect estimates (Table S5). The intercept of MR-Egger regression test did not identify any horizontal pleiotropy and/or violation of the InSIDE assumption (*P* = 0.75) and the *Q*-statistic did not indicate heterogeneity (*P* = 0.56). After removing the UK Biobank data case–control study (4800 cases and 20,289 controls) from the outcome population, the CI of effect sizes was wider (Table S5).

Implementing the MR-PRESSO and leave-one-out methods highlight rs429358 as an outlier (Fig. S2). This SNP maps to the APOE gene and showed the strongest association with PA. It is also associated with multiple traits, including total cholesterol, low-density lipoprotein cholesterol, triglyceride and Alzheimer’s disease.^38,39^ This finding was supported by searching from GWAS Catalogue and PhenoScanner (Table S3). Therefore, we generated the instrumental variable by using seven SNPs for MVPA after removing rs429358. The effect sizes of the seven MVPA SNPs with MVPA and with colorectal cancer were presented in Table S6.

Evidence for a causal association was detected between AMPA and colorectal cancer risk by using both five SNPs at *P* < 5 × 10^−8^ and three SNPs at *P* < 5 × 10^−9^ as genetic instruments (Fig. 2). In particular, the ORs of inverse variance-weighted MR were 0.60 (95% CI: 0.41, 0.88) and 0.54 (95% CI: 0.35, 0.86) for each genetic instrument (Figs. 2 and S1). All sensitivity analyses showed a similar effect size of the association between AMPA and colorectal cancer risk (Table S5). The *Q*-statistic suggested no heterogeneity (*P* = 0.34 and 0.35, respectively) and the intercept of MR-Egger suggested no pleiotropy. Removing UK Biobank colorectal cancer GWAS from the outcome populations narrowed the CIs of the effect sizes (Table S5). No association was detected between sedentary time and colorectal cancer risk and no pleiotropy or heterogeneity was indicated (Fig. 2 and Table S5). The effect estimates for each instrumental variable on AMPA and colorectal cancer or sedentary time and colorectal cancer were listed in Table S6.

Two-sample MR on the associations between measures of body fatness and colorectal cancer risk, as well as the effect estimates of each SNP on exposures were listed in Table S7–10. BMI, body fat percentage, waist circumference and AFR were significantly associated with increased colorectal cancer risk, with OR (95% CI) 1.24 (1.11, 1.39), 1.14 (1.03, 1.25), 1.19 (1.04, 1.36) and 1.50 (1.17, 1.93), respectively (Fig. 2). We did not observe any significant associations between TFR or LFR and colorectal cancer risk.

### Multivariable MR

**Fig. 3:**
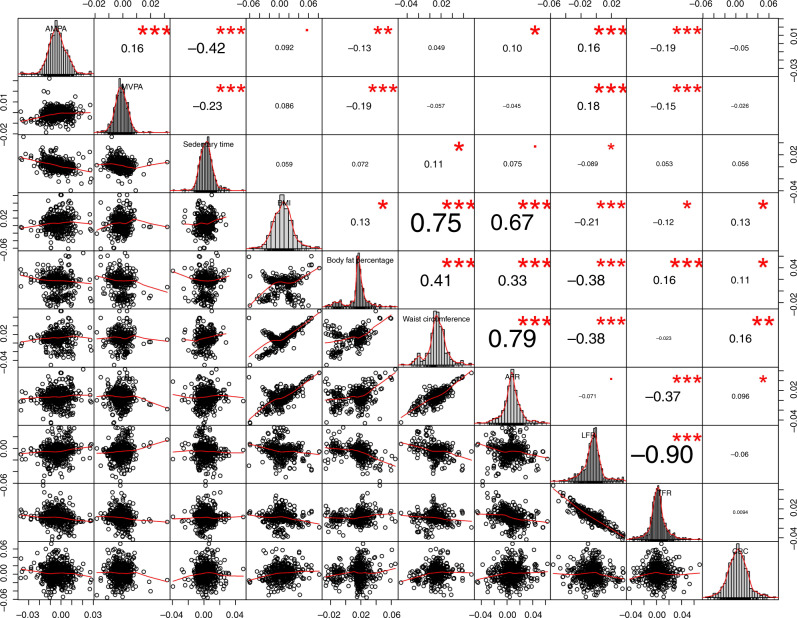
Genetic correlation coefficient between each covariate. MVPA self-reported moderate-to-vigorous physical activity, AMPA acceleration vector magnitude physical activity, BMI body mass index, CRC colorectal cancer, AFR arm fat ratio, LFR leg fat ratio, TFR trunk fat ratio.

Instrumental variables for BMI, waist circumference and AFR were highly correlated as their correlation coefficients (*r*) range from 0.67 to 0.79 (Fig. 3). Body fat percentage was positively correlated with BMI (*r* = 0.13), waist circumference (*r* = 0.41), AFR (*r* = 0.33) and TFR (*r* = 0.16). LFR and TFR were highly inversely correlated (*r* = −0.90). MVPA and AMPA were positively correlated with each other (*r* = 0.16) and negatively correlated with sedentary time (*r* = −0.23 and −0.42 respectively). The three measures of PA were not correlated with colorectal cancer risk.

We estimated the direct effect of MVPA and AMPA and four different measures of obesity and overweight on colorectal cancer risk by applying the multivariable MR method (Table 2). The direct effect sizes (OR) of MVPA or AMPA on colorectal cancer when BMI was kept constant were 0.56 (95% CI: 0.36, 0.89) and 0.70 (95% CI: 0.52, 0.96), respectively. The direct effect sizes (OR) of BMI on colorectal cancer when MVPA or AMPA was kept constant were 1.24 (95% CI: 1.12, 1.37) and 1.22 (95% CI: 1.09, 1.36), respectively. There was no statistically significant heterogeneity and MR-Egger did not indicate a significant pleiotropy effect. Compared to the effect estimates from two-sample MR, the estimated CIs of the two types of PA from multivariable MR became much wider, while the BMI effects were not affected (Table 2 and Fig. 2). Results from two-step network MR also indicated a partial mediation effect of BMI on the PA-colorectal cancer association (Table S11).

**Table 2:** Results of multivariable Mendelian randomisation analysis: causal estimation of MVPA, AMPA and measures of body fatness on colorectal cancer risk.

	Methods							
	IVW				MR-Egger			
	OR (95% CI)	*P*	*P*_int_^a^	*P*_het_^b^	OR (95% CI)	*P*	*P*_int_^a^	*P*_het_^b^
Model 1 (AMPA + BMI)								
AMPA	0.70 (0.52, 0.96)	0.02	/	0.15	0.71 (0.46, 1.10)	0.13	0.95	0.13
BMI	1.22 (1.09,1.36)	4.65E – 04			1.01 (0.83, 1.23)	0.001		
Model 2 (AMPA + body fat percentage)								
AMPA	0.86 (0.67, 1.10)	0.24	/	9.74E – 13	0.83 (0.59, 1.15)	0.26	0.70	8.01E – 13
Body fat percentage	1.10 (0.98, 1.24)	0.10			1.11 (0.98, 1.25)	0.09		
Model 3 (AMPA + waist circumference)								
AMPA	0.61 (0.39, 0.97)	0.04	/	0.05	0.61 (0.39, 0.97)	0.04	0.26	0.05
Waist circumference	1.21 (1.07, 1.36)	0.003			1.21 (1.07, 1.36)	0.003		
Model 4 (AMPA + AFR)								
AMPA	0.64 (0.45, 0.92)	0.02	/	0.35	0.52 (0.32, 0.84)	0.01	0.23	0.49
AFR	1.46 (1.09, 1.97)	0.01			1.47 (1.15, 1.89)	0.002		
Model 5 (MVPA + BMI)								
MVPA	0.56 (0.36, 0.89)	0.01	/	0.22	0.58 (0.29, 1.15)	0.12	0.91	0.19
BMI	1.24 (1.12, 1.37)	7.67E – 5			1.24 (1.11, 1.38)	1.32E – 4		
Model 6 (MVPA + body fat percentage)								
MVPA	0.84 (0.56, 1.26)	0.41	/	3.00E – 12	0.60 (0.33, 1.06)	0.08	0.10	5.36E – 12
Body fat percentage	1.12 (1.02, 1.24)	0.02			1.13 (1.02, 1.25)	0.02		
Model 7 (MVPA + waist circumference)								
MVPA	0.70 (0.42, 1.17)	0.18	/	0.05	0.64 (0.30, 1.34)	0.24	0.73	0.05
Waist circumference	1.19 (1.05, 1.34)	0.01			1.20 (1.05, 1.36)	0.01		
Model 8 (MVPA + AFR)								
MVPA	0.58 (0.33, 1.02)	0.06	/	0.50	0.44 (0.20, 0.93)	0.03	0.27	0.52
AFR	1.39 (1.09, 1.79)	0.01			1.42 (1.10, 1.82)	0.01		

*MVPA* self-reported moderate-to-vigorous physical activity, *AMPA* acceleration vector magnitude physical activity, *BMI* body mass index, *AFR* arm fat ratio, *OR* odds ratio, *CI* confidence interval, *IVW* inverse variance-weighted, *P P* value for the effect estimate.

^a^*P*_int_: *P* value for the intercept of MR-Egger’s test.

^b^*P*_het_: *P* value of *χ*^2^ *Q* test for heterogeneity.

The direct effect sizes (OR [95% CI]) of MVPA or AMPA on colorectal cancer when AFR was kept constant were 0.58 (0.33, 1.02) and 0.64 (0.45, 0.92), respectively. The direct effect sizes of AMPA on colorectal cancer when waist circumference was kept constant were 0.61 (0.39, 0.97), while no association observed for MVPA after adjusting for waist circumference. The associations between MVPA or AMPA and colorectal cancer risk disappeared when keeping body fat percentage constant.

**Fig. 4:**
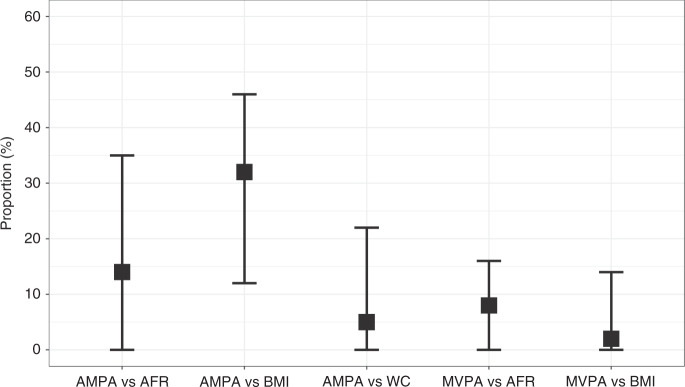
Proportion estimates through BMI and body fat composition. BMI body mass index, MVPA self-reported moderate-to-vigorous physical activity, AMPA acceleration vector magnitude physical activity, AFR arm fat ratio, WC waist circumference, AMPA vs AFR the proportion of effects of AMPA on colorectal cancer risk mediated through AFR, AMPA vs BMI the proportion of effects of AMPA on colorectal cancer risk mediated through BMI, AMPA vs WC the proportion of effects of AMPA on colorectal cancer risk mediated through WC, MVPA vs AFR: the proportion of effects of AMPA on colorectal cancer risk mediated through AFR, MVPA vs BMI the proportion of effects of AMPA on colorectal cancer risk mediated through BMI.

Based on the total effect estimates from two-sample MR and direct effect estimates from multivariable MR, we evaluated the proportion of effects of PA on colorectal cancer risk mediated through measures of body fat. The attenuated direct effects indicated that part of the effects of the two measures of PA on colorectal cancer was mediated through BMI or AFR. AMPA also can affect colorectal cancer risk through waist circumference. For the effects of MVPA and AMPA on colorectal cancer risk, the proportion mediated through BMI was 2% (95% CI: 0, 14) and 32% (95% CI: 12, 46), respectively, while the proportion mediated through AFR was 8% (95% CI: 0, 16) and 14% (95% CI: 0, 35), respectively (Fig. 4). The proportion of effects mediated through waist circumference was 5% (95% CI: 0, 22) for AMPA (Fig. 4).

## Discussion

Colorectal cancer is a common cancer with appreciable morbidity and mortality. Prospective cohort, case–control, and cross-sectional observational studies support an inverse association between PA and colorectal cancer risk.^40–42^ The association between AMPA and colorectal cancer risk has been reported in a previous MR study.^8^ However, the mechanism(s) through which PA influences colorectal cancer risk is not clear yet, especially the relative role of body weight and distribution in this association. Applying two-sample and multivariable MR approaches, we used genetic variants from large GWAS as instruments to explore whether the apparent beneficial effects of three measures of PA (MVPA, AMPA and sedentary time) on colorectal cancer risk are mediated through body fatness. Our results show that both lower MVPA and AMPA increase colorectal cancer risk, both independently and through body fatness. In line with our findings, a meta-analysis has indicated that PA is associated with colorectal cancer risk in both high and low BMI groups.^43^

It is important to note that we found that increasing PA causes decreased colorectal cancer risk independent of measures of obesity and body fat distribution. One SD of MVPA is ~4.96 metabolic equivalent task (MET)-h/day. MET is an objectively measured ratio of energy expenditure relative to the mass of a person when performing PA compared to sitting quietly. Although there is no standard method to transform milli-gravities (accelerometer measurement units) to energy expenditure, each SD of AMPA was 8.14 milli-gravities (or 0.08 m/s^2^), which approximates to 3 MET-h/day.^18^ For each SD increase in MVPA or AMPA, colorectal cancer risk decreased by 44% and 40%, respectively. These estimates imply that if individuals replace daily sedentary behaviour with 20–90 min of MVPA or with 13–60 min accumulated MVPA, their risk of colorectal cancer will decrease by 40% (Table S12).^44^ In our study, MVPA estimated a longer time spending on PA compared to the estimation by AMPA to achieve a similar benefit of colorectal cancer risk, which is consistent with the existing evidence that MVPA tends to overestimate time engaged in PA in the general population.^45^ Part of the decreased effect on colorectal cancer risk (2% [95% CI: 0, 14] and 8% [95% CI: 0, 16] for MVPA and 32% [95% CI: 12, 46] and 14% [95% CI: 0, 35] for AMPA) was mediated through the effects of BMI and AFR, respectively. Since the genetic instrument of MVPA is weaker compare to AMPA, the BMI and AFR mediation effect for MVPA may be underestimated.^46^

Diverse biological mechanisms have been proposed to explain the observed inverse association between PA and colorectal cancer. These include beneficial effects on bowel transit time,^47^ immune system reactions,^48^ metabolisms of bile acid, better insulin sensitivity^49^ and the reduction of prostaglandin E2 levels in colonic mucosa.^50^ Evidence from RCTs supports that PA can reduce the bowel transit time and therefore reduce the time of contact between carcinogens and colonic mucosa.^47^ The decrease of prostaglandin E2 synthesis may also promote intestinal peristalsis and hence reduce transit time.^51^ Besides, prostaglandin E2 can promote tumour generation directly or through its multifaceted effects on inflammation.^52^ PA also results in a lower concentration of bile acid, which is an essential mediator of the cholesterol mechanism and the lower bile acid concentration is associated with lower blood triglycerides.^53^ The effect between PA and colorectal cancer risk could be through these pathways, although none of the genetic variants included as an instrumental variable for PA was located within genes involved in indicators of above-mentioned metabolism pathways. In addition, regular moderate PA may have a benefit on natural cytotoxicity and T-lymphocyte proliferation, on reducing the production of pro-inflammatory cytokines and on increasing the count of T cells, B cells and immunoglobulins.^48^

### Strengths and limitations

One of the strengths of this study was that we explored both subjective and objective measures of PA (MVPA, AMPA and sedentary time). Previous studies showed that there are discrepancies between MVPA and AMPA.^54^ Compared to MVPA where recall and reporting bias are problematic, AMPA explains 44–47% variance of energy expenditure.^55^ MVPA tends to overestimate time engaged in PA in the general population.^45^ Nevertheless, MVPA is commonly used in epidemiological and observational studies, because it is data that is readily collected and inexpensive. Our results for MVPA and AMPA were consistent, with both supporting a causal effect of PA in reducing cancer risk. The confidence intervals for the effect estimates observed for AMPA were appreciably narrower than for MVPA, suggesting the possibility of recall bias, but the health-promoting effect of actually wearing an accelerometer might also influence our results.^56^ Nonetheless, our use of a variety of instrumental variable methods provides new insight into the effect of PA on cancer risk. Another main advantage of our study is that we have clarified the association pathways among three measures of PA, different measures of body fatness and colorectal cancer for the first time, including the total effect and direct effect, as well as the proportion of the indirect effect. Several measures of body fatness have been considered in our study, BMI and body fat percentage represent two different measures of total body fat while waist circumference, AFR, TFR and LFR represent body fat in different areas. In the two-sample MR study, BMI, body fat percentage, waist circumference and AFR are associated with colorectal cancer risk, which is consistent with the previous evidence.^12,57^ Based on the results of multivariable MR, AMPA can affect colorectal cancer risk both through and independent of BMI, waist circumference and AFR, while MVPA can affect colorectal cancer risk both through and independent of BMI and AFR. After adjusting for body fat percentage, associations between AMPA or MVPA and colorectal cancer risk disappeared, which may be due to the high heterogeneity introduced by using hundreds of SNPs as the instrumental variable. Papdimitriou et al. performed an MR on PA and CRC risk and they also considered the effects of BMI in the sensitivity analysis. In the current study, we have expanded previous work by additionally including genetic instruments for measures of body fatness along with PA in multivariable MR.

We acknowledge that the study has several limitations. First, although we derived instrumental variables from the largest available GWAS for PA, the SNPs for self-reported PA explain only 0.07% of the variance in MVPA. However, the calculated *F*-statistic (*F*-statistic = 273) reached a widely-accepted threshold level.^37^ As a result, our analysis of MVPA was underpowered (<0.8). Second, with a 21% SNP heritability for AMPA and 5% for MVPA, the low variance of genetic instruments (AMPA: 0.2%; MVPA: 0.07%) may imply that the current discovered SNPs cannot be considered as powerful proxies for PA. Furthermore, although we applied the most up-to-date MR methods, we cannot completely rule out any potential horizontal pleiotropy until we know all biological functions for each SNP. Third, in two-sample MR analysis, weak instrument bias is in the direction of the null while the partial overlapping data between exposure and outcome from UK Biobank may bias against the null.^29^ However, the sensitivity analysis by removing UK Biobank participants from the colorectal cancer GWAS broadens the CI for association with MVPA while narrowing the CI for AMPA slightly. The overall results did not change. Fourth, sBIA is not a perfect method to measure body fat distribution. However, the correlation between body fat measured by sBIA and MRI is ~0.8^58^ and there is no available GWAS with enough power on MRI or DEXA measured body fat distribution or body fat percentage available. Fifth, because we do not have access to individual-level data we did not perform stratified analysis by gender or tumour site, even though evidence from a European multinational cohort study showed that PA was associated with proximal colon cancer and distal colon cancer risk but not with rectal cancer risk.^59^ Hence, we may have underestimated the effect of PA on colonic cancer risk in our dataset by including rectal cancer. Finally, all analyses were performed using instrumental variables and summary level data derived from the GWAS on individuals of European ancestry, which may impact the generalisability of our findings to non-European populations.

## Conclusions

The results of this study establish a causal role of both subjectively and objectively measured PA in colorectal cancer risk, independent of the obesity and body fat distribution. Our results suggest that promoting and facilitating exercise could result in a decrease in colorectal cancer incidence, regardless of individuals’ weight or body fat distribution.

## Supplementary information

Supplementary File

## Data Availability

Availability of colorectal cancer GWASs was described by Law et al.^14^ (PMID 31089142). Availability of self-reported habitual physical activity GWAS was described by Klimentidis et al.^15^ (PMID 29899525). The summary-level data from the average acceleration vector magnitude GWAS was available at Doherty et al.^16^ (PMID 30531941). The summary-level data for GWAS on BMI, body fat percentage, waist circumference and body fat distribution were extracted from Locke et al.^60^ (PMID 25673413), Bycroft et al.^61^ (PMID 30305743), Shungin et al.^62^ (PMID 25673412) and Ralk-Andersen et al.^11^ (PMID 30664634), respectively. All data generated during this study are included in this published article and its supplementary information files.
