# Supplementary material for: Genetically predicted physical activity levels are associated with lower colorectal cancer risk: a Mendelian randomisation study
Source: Br J Cancer. 2021 Jan 29;124(7):1330–8. doi: 10.1038/s41416-020-01236-2 (PMC8007642; doi:10.1038/s41416-020-01236-2)
Supplement: Supplementary file 1 — Supplementary File [file 41416_2020_1236_MOESM1_ESM.docx]

**Title:**

**Genetically-predicted physical activity levels are associated with lower colorectal cancer risk: a Mendelian Randomization Study.**

**Running title: Physical activity reduces colorectal cancer risk**

Authors: Xiaomeng Zhang^1,*^, Evropi Theodoratou^1,2,*^, Xue Li^1,3^, Susan M Farrington^4^, Philip J Law^5^, Peter Broderick^5^, Marion Walker^4^, Yann C Klimentidis^6^, Jessica MB Rees^7^, Richard S Houlston^5^, Ian PM Tomlinson^2^, Stephen Burgess^8^, Harry Campbell^1^, Malcolm G Dunlop^4,#^, Maria Timofeeva^4,9, #^

^1^ Centre for Global Health Research, Usher Institute, University of Edinburgh, Edinburgh, UK

^2^ Cancer Research UK Edinburgh Centre, Medical Research Council Institute of Genetics and Molecular Medicine, University of Edinburgh, Edinburgh, UK

^3^ School of Public Health and the Second Affiliated Hospital, Zhejiang University, Hangzhou, China

^4^ Colon Cancer Genetics Group, Cancer Research UK Edinburgh Centre and Medical Research Council Human Genetics Unit, Medical Research Council Institute of Genetics and Molecular Medicine, University of Edinburgh, Edinburgh, UK

^5^ Division of Genetics and Epidemiology, The Institute of Cancer Research, London, UK

^6^ Department of Epidemiology and Biostatistics, Mel and Enid Zuckerman College of Public Health, University of Arizona, Arizona, USA

^7^ Edinburgh Clinical Trials Unit, Centre for Global Health Research, Usher Institute, University of Edinburgh, Edinburgh, UK

^8^ MRC Biostatistics Unit, University of Cambridge, Cambridge, UK

^9^ Danish Institute for Advanced Study (DIAS), Department of Public Health, University of Southern Denmark, Odense, Denmark

^#^Correspondence to

Dr. Maria Timofeeva (mtimofeeva@health.sdu.dk), Danish Institute for Advanced Study (D-IAS), Department of Public Health, University of Southern Denmark, J.B. Winsløws Vej 9, DK-5000 Odense C, Denmark

Prof Malcolm G Dunlop ([malcolm.dunlop@ed.ac.uk](mailto:malcolm.dunlop@ed.ac.uk)), Colon Cancer Genetics Group, Cancer Research UK Edinburgh Centre and Medical Research Council Human Genetics Unit, Medical Research Council Institute of Genetics and Molecular Medicine, University of Edinburgh, Edinburgh, EH4 2XU, UK

^*^ Authors contributed equally at these positions

Contents

[Supplementary methods 3](#_Toc57824808)

[Genome-wide association study of colorectal cancer risk 3](#_Toc57824809)

[Description of measures of physical activity 3](#_Toc57824810)

[Instrumental variable for measures of body fatness 3](#_Toc57824811)

[Valid instrumental variables assumption estimation 4](#_Toc57824812)

[Supplementary tables 5](#_Toc57824813)

[Table S1 Summary of studies used in the meta-analysis of genome-wide association studies of colorectal cancer 5](#_Toc57824814)

[Table S2 Genetic variants associated with three continuous physical activities recorded from the physical activity GWASs. 6](#_Toc57824815)

[Table S3 Searching results from GWAS Catalog and Phenoscanner for SNPs of physical activity 7](#_Toc57824816)

[Table S4 Effect sizes can be detected with the power of 0.8 given the sample size, proportion of cases and variance explained by instrumental variable for each Mendelian randomisation analysis 11](#_Toc57824817)

[Table S5 Results of Two-sample Mendelian randomisation analyses of physical activity on colorectal cancer risk 11](#_Toc57824818)

[Table S6 Summary of associations between genetic variants and physical activity and colorectal cancer risk 12](#_Toc57824819)

[Table S7 Genetic variants associated with body mass index recorded by Locke (2015) 13](#_Toc57824820)

[Table S8 Genetic variants associated with body fat percentage recorded by Bycroft (2018) 14](#_Toc57824821)

[Table S9 Genetic variants associated with waist circumference recorded by Shungin (2015) 20](#_Toc57824822)

[Table S10 Genetic variants associated with body fat distribution 21](#_Toc57824823)

[Table S11 Results of network analysis 24](#_Toc57824824)

[Table S12 Time spend on physical activity to achieve risk reduction of colorectal cancer 25](#_Toc57824825)

[Supplementary figures 25](#_Toc57824826)

[Figure S1 Visualisation of Mendelian randomisation analysis of the effect of self-reported moderate-to-vigorous physical activity (MVPA), acceleration vector magnitude physical activity (AMPA) and sedentary time on colorectal cancer risk 25](#_Toc57824827)

[Figure S2 Results of Mendelian randomisation (MR) analysis using MR-PRESSO and leave-one-out methods for the association between self-reported moderate-to-vigorous physical activity (MVPA) and colorectal cancer (CRC) risk 27](#_Toc57824828)

[Reference 28](#_Toc57824829)

# Supplementary methods

## Genome-wide association study of colorectal cancer risk

The colorectal cancer summary statistics were obtained from a previously published meta-analysis of 15 genome-wide association studies of colorectal cancer.^1^ Additionally, for sensitivity analysis, the original meta-analysis was re-run to exclude UK Biobank case-control GWAS study. Meta-analyses were performed using the fixed-effects inverse-variance method using META v1.7.^2^ Meta-analysis and imputation refine the association of 15q25 with smoking quantity.^3^ Cochran’s Q-statistic to test for heterogeneity and the I^2^ statistic to quantify the proportion of the total variation due to heterogeneity were calculated. The summary characteristics and sample size of the included studies are presented in Table S1.

## Description of measures of physical activity

UK Biobank collected physical activity data during work and leisure time through a self-reported questionnaire and 7 days accelerometer wearing.^4, 5^ For self-reported moderate PA (MPA) and vigorous PA (VPA), participants were asked: ‘In a typical WEEK, on how many days did you do 10 min or more of moderate physical activities like carrying light loads, cycling at a normal pace? (Do not include walking)’ and ‘In a typical WEEK, how many days did you do 10 min or more of vigorous physical activity? (These are activities that make you sweat or breathe hard such as fast cycling, aerobics, heavy lifting)’ respectively. For each of these questions, those who indicated 1 or more such days were then asked, ‘How many minutes did you usually spend doing moderate/vigorous activities on a typical DAY’. Moderate-to-vigorous PA (MVPA) was calculated by taking the sum of total minutes/week of MPA multiplied by four and the total number of VPA minutes/week multiplied by eight. In the UK Biobank, about 103712 datasets collected from participants wearing an Axivity AX3 wrist-worn accelerometer for seven days. We included a measure from accelerometer wearing: overall acceleration vector magnitude measured PA (AMPA) and sedentary time. The unit for physical activity vector magnitude is milli-gravities (mg) and the calculation described in a UK Biobank study.^5^ The details of self-reported MVPA and AMPA were summarized in Table S2 and Table S6.

## Instrumental variable for measures of body fatness

Sixty-eight SNPs were extracted from Locke et al at p<5×10^-8^ (a GWAS based on 322 154 individuals of European ancestry) to generate the genetic instrument for BMI (mean and standard deviation (SD) of BMI were 27.37kg/m^2^ and 4.48kg/m^2^ respectively).^6^ Three hundred and seventy significant SNPs were extracted from Bycroft et al at p<5×10^-8^ (a GWAS based on 331 117 participants of European ancestry) to generate the genetic instrument for body fat percentage.^7^ Fifty-nine significant SNP were extracted from Shungin et al at p<5×10^-8^ (a GWAS based on 224 459 participants of European ancestry) to generate the genetic instrument for waist circumference.^8^ Sixty-nine significant SNPs for both genders were extracted from Rask-Andersen et al at p<5×10^-8^ (a GWAS performed among bio-electrical impedance analysis data from UK Biobank) as the genetic instrument of body fat distribution, which included 15 SNPs for arm fat ratio (AFR), 50 SNPs for trunk fat ratio (TFR) and 46 SNPs for leg fat ratio (LFR) (Table 1, Figure 1).^9^ Rask-Andersen et al has reported 26 SNPs associated with AFR, considering the correlation between AFR and TFR (or LFR), we take the 15 SNPs that exclusively associated with AFR as the instrumental variable of AFR.

## Valid instrumental variables assumption estimation

We explored whether horizontal pleiotropy could have biased our results before applying the PA instrumental variables to perform a two-sample MR. We checked the GWAS Catalog and PhenoScanner to investigate whether the PA instrumental variables were associated with other traits. Apart from the measures of body fatness, we found that rs2696625 and rs2854277 are also associated with neuroticism and schizophrenia respectively (Table S3). Furthermore, a recent MR study supported a protective effect of PA on major depressive disorder.^10^ However, there is no known epidemiological link between depression and CRC risk^11^, and so both SNPs were retained for all analyses.

**Network (two-step) Mendelian randomisation**

To estimate the proportion of the effect of PA on CRC mediated through BMI, we conducted network MR.***^12-14^*** The first step was to perform a two-sample MR of PA on BMI and the second step was to perform a two-sample MR of BMI on CRC. For the first step, effect estimates of the SNPs on PA were obtained from the two PA GWASs respectively. Effect estimates of these PA SNPs on BMI were obtained from the BMI GWAS.***^6^*** For the second step, 68 SNPs (P<5×10^-8^) for BMI were extracted from Locke et al. and the effect sizes of these SNPs on CRC were obtained from the large meta-analysis of 15 primary CRC GWAS.***^1^*** If SNPs for the exposure phenotypes (PA or BMI) were missing in the summary statistics of the outcome phenotypes (BMI or CRC), we used proxy variants in high LD (r^2^≥0.8). The causal effects and the corresponding standard errors were calculated by using random-effects inverse-variant weighted method.

# Supplementary tables

| Table S1 Summary of studies used in the meta-analysis of genome-wide association studies of colorectal cancer | | | |
| --- | --- | --- | --- |
| **Series** | **Study setting** | **Sampling** | **Number of cases and controls post quality control** |
| *CCFR1* | Colon Cancer Family Registry | Recently diagnosed cases reported to population complete cancer registries in the USA (Seattle Familial Colorectal Cancer Registry). Canada (Ontario Familial Cancer Registry) and Australia (Australasian Colorectal Cancer Family Study). Population-based controls | 1,175 cases  999 controls |
| *CCFR2* | Colon Cancer Family Registry | Cases from the Colon Cancer Family Registry, recruited from centres in Australia, Ontario, Seattle, USC, Mayo and Hawaii. Controls from the Cancer Genetic Markers of Susceptibility studies of breast and prostate cancer | 795 cases  2,234 controls |
| *COIN* | COIN trial | Multicentre study of cetuximab and other therapies in metastatic CRC. Cases recruited as a clinical-based series and controls as population-based series. Controls were unselected blood donors | 1,950 cases  2,162 controls |
| *CORSA* | CORSA (COloRectal cancer Study of Austria) | Population based screening study in colorectal cancer and adenoma | 919 cases  788 controls |
| *Croatia* | Case-control study, Cases recruited from a teaching hospital in Zagreb | Recently diagnosed cases. Spouses controls from the same population | 689 cases  441 controls |
| *DACHS* | DACHS Study, excluding previously reported cases and controls (GECCO study) | Population-based incidence cases aged>30 at diagnosis in the Rhine-Neckar-Odenwald region (southwest Germany). Community-based controls were randomly selected from population registries, matched by age (5-year groups), sex, and county | 1,028 cases  661 controls |
| *FIN* | Finnish Colorectal Cancer Predisposition Study | Cases requited through Finnish Hospitals and Finnish Cancer Registry. Population-based controls from FINRISK, Health 2000, Finnish Twin Cohort and Helsinki Birth Cohort Studies | 1,172 cases  8,266 controls |
| *NSCCG-OncoArray* | National Study of Colorectal Cancer Genetics | Patients were selected for having a family history of CRC (at least one first degree relative) or age of diagnosis below 58. Controls obtained from cancer-free patients from the BCAC and PRACTICAL studies | 6,596 cases  7,205 controls |
| *SCOT* | Short Course Oncology Treatment (SCOT) trial | Cases obtained from a study of adjuvant chemotherapy in colorectal cancer by the CACTUS and OCTO groups. Controls comprised of cancer-free individuals from The Heinz Nixdorf Recall study | 2,910 cases  4,095 controls |
| *Scotland1* | COGS (Colorectal Cancer Susceptibility Study) | Population-based incidence cases aged <55 at diagnosis; Scotland. Population-based controls frequency matched by area of residence within Scotland | 932 cases  943 controls |
| *SOCCS/GS* | Scottish Colorectal Cancer Study 3 (SOCCS3), Generation Scotland | Population based incident cases (Scottish colorectal cancer study 3), and population based controls from Scotland (Generation Scotland and Scottish colorectal cancer study 3 controls) | 4,551 cases  8,804 controls |
| *SOCCS/LBC* | Scottish Colorectal Cancer Study 3 (SOCCS3), Lothian Birth Cohort | Population based incidence cases from Scotland. Population based controls from the Lothian Birth Cohorts 1921 and 1936 | 996 cases  1,297 controls |
| *UK1* | CORGI (Colorectal Tumour Gene Identification Consortium) | Cases enriched for family history of CRC, ascertained through UK clinical genetics clinics. Spouse controls with no personal history or family history of CRC. | 890 cases  900 controls |
| *VQ58* | VICTOR, post treatment stager of a phase III, randomised trial of rofecoxib (VIOXX) in patients after potentially curative therapy. QUASAR2, multi-centre study of capectibine±bevacizumb as adjuvant treatment. 1958 Birth cohort controls | Cases recruited as a clinical-based series, controls as population-based series | 932 cases  943 controls |
| *UK Biobank* | Cases: prevalence (~51%) and incidence cases of colorectal cancer across UK: controls: population based controls without history of cancer | Population based incidence and prevalence cases of colorectal cancer,UK. Population based controls without history of cancer and/or colorectal adenoma matched 1:4 by age, gender, date of blood draw, ethnicity, and region of residence (two first letters of postal code) | 4,800 cases  20,289 controls |

| Table S2 Genetic variants associated with three continuous physical activities recorded from the physical activity GWASs. | | | | | | | |
| --- | --- | --- | --- | --- | --- | --- | --- |
| **Category** | **Summary** | **rsid** | **Chr** | **Closest gene** | **Position** | **EA** | **EAF** |
| Moderate-to-vigorous physical activity (MVPA)^4^(MET-minutes/week)  Heritability=5% | **Description:**  N=337,234  Age: range=40-69, mean=56.5, SD=8.1  Sex: female=45.6%  BMI (Kg/m^2^): mean=27.4, SD=4.8, N=499,518  MVPA: Mean=1650, Median=960, SD=2084  **Analysis:**  Linear regression model adjusted age, sex, genotyping chip, first ten genomic principal components, centre and season at centre visit or wearing accelerometer. | rs2988004 | 9 | PAX5 | 37,044,388 | T | 0.56 |
|  |  | rs2035562 | 3 | CADM2 | 85,056,521 | A | 0.33 |
|  |  | rs149943 | 6 | ZNF165 | 28,002,388 | G | 0.85 |
|  |  | rs3094622 | 6 | RPP21 | 30,327,952 | A | 0.86 |
|  |  | rs2854277 | 6 | HLA-DQB1 | 32,628,084 | C | 0.92 |
|  |  | rs7804463 | 7 | EXOC4 | 133,447,651 | T | 0.53 |
|  |  | rs7791992 | 7 | C7orf72/SPATA48 | 50,237,784 | C | 0.41 |
|  | | | | | | | |
| The overall acceleration vector magnitude PA (AMPA) (milli-gravities)^15^  Heritability=21% | **Description:**  N=91,105; Age:45-79; Sex: female=56.3%  Mean=31 for people aged 45-54 (20%)  Mean=29 for people aged 55-64 (35%)  Mean=26 for people aged 65-74 (42%)  Mean=23 for people aged 75-79 (3%)  **Analysis:**  Linear mixed model adjusted assessment centre, genotyping array, age, age squared, and season of wear. | rs2696625 | 17 | KANSL1-AS1 | 44,326,864 | A | 0.77 |
|  |  | rs59499656 | 18 | RIT2/SYT4 | 40,768,309 | A | 0.66 |
|  |  | rs6895232 | 5 | LINC01470 | 152,039,421 | T | 0.66 |
|  |  | rs6775319 | 3 | SATB1-AS1 | 18,758,501 | A | 0.27 |
|  |  | rs564819152 | 10 | SKIDA1 | 21,820,650 | A | 0.68 |
| The sedentary time (milli-gravities)^15^  Heritability=12.9% |  | rs26579 | 5 | MEF2C-AS2 | 87,985,295 | G | 0.42 |
|  |  | rs25981 | 5 | EFNA5 | 106,822,908 | G | 0.53 |
|  |  | rs1858242 | 3 | LOC105377146 | 68,527,135 | A | 0.26 |
|  |  | rs34858520 | 7 | CALN1 | 71,723,883 | A | 0.56 |
|  |  | rs6870096 | 5 | NMUR2 | 151,945,811 | C | 0.32 |
|  |  | rs61776614 | 1 | FAAP20 | 2,166,406 | C | 0.93 |
| PA: physical activity, genetic variants for PA were extracted from two physical activity GWASs base on UK Biobank data. SD: standard deviation, EA: effect allele, EAF: effect allele frequency, BMI: body mass index | | | | | | | |

| Table S3 Searching results from GWAS Catalog and Phenoscanner for SNPs of physical activity | | | | |
| --- | --- | --- | --- | --- |
| rsid | Phenoscanner and GWAS CATALOG | Study | PMID | Ancestry |
| AMPA | | | | |
| rs2696625* | Types of human blood cell traits | Astle W | 27863252 | European |
|  | Invasive ovarian cancer | Phelan M | 28346442 | European |
|  | Neuroticism | SSGAC | 29292387 | European |
| rs55657917* | Types of human blood cell traits | Astle W | 27863252 | European |
|  | Invasive ovarian cancer | Phelan M | 28346442 | European |
|  | Neuroticism | SSGAC | 27089181 | European |
|  | Alcohol intake frequency | Neale B | UKBB | European |
|  | Comparative height size at age 10 | Neale B | UKBB | European |
|  | Daytime dozing or sleeping | Neale B | UKBB | European |
|  | Fed-up feelings | Neale B | UKBB | European |
|  | Forced expiratory volume in 1-second | Neale B | UKBB | European |
|  | Forced expiratory volume in 1-second, best measure | Neale B | UKBB | European |
|  | Forced expiratory volume in 1-second, predicted percentage | Neale B | UKBB | European |
|  | Forced vital capacity | Neale B | UKBB | European |
|  | Forced vital capacity, best measure | Neale B | UKBB | European |
|  | Frequency of tenseness or restlessness in last 2 weeks | Neale B | UKBB | European |
|  | Hair or balding pattern: pattern 3 | Neale B | UKBB | European |
|  | Hair or balding pattern: pattern 4 | Neale B | UKBB | European |
|  | Hand grip strength | Neale B | UKBB | European |
|  | Heel bone mineral density | Neale B | UKBB | European |
|  | Height | Neale B | UKBB | European |
|  | Illnesses of mother: parkinsons disease | Neale B | UKBB | European |
|  | Impedance of arm left/arm right/ whole body | Neale B | UKBB | European |
|  | Irritability | Neale B | UKBB | European |
|  | Medication for pain relief, constipation, heartburn: none of the above | Neale B | UKBB | European |
|  | Medication for pain relief, constipation, heartburn: paracetamol | Neale B | UKBB | European |
|  | Miserableness | Neale B | UKBB | European |
|  | Mood swings | Neale B | UKBB | European |
|  | Mouth or teeth dental problems: mouth ulcers | Neale B | UKBB | European |
|  | Nap during day | Neale B | UKBB | European |
|  | Nervous feelings | Neale B | UKBB | European |
|  | Neuroticism score | Neale B | UKBB | European |
|  | Pain type experienced in last month: headache | Neale B | UKBB | European |
|  | Peak expiratory flow | Neale B | UKBB | European |
|  | Qualifications: college or university degree | Neale B | UKBB | European |
|  | Relative age of first facial hair | Neale B | UKBB | European |
|  | Relative age voice broke | Neale B | UKBB | European |
|  | Sensitivity or hurt feelings | Neale B | UKBB | European |
|  | Sitting height | Neale B | UKBB | European |
|  | Systolic blood pressure | Neale B | UKBB | European |
|  | Treatment with paracetamol | Neale B | UKBB | European |
|  | Worrier or anxious feelings | Neale B | UKBB | European |
| rs59499656 | Arm/Leg/trunk fat mass | Neale B | UKBB | European |
|  | Arm/body/leg/trunk fat percentage | Neale B | UKBB | European |
|  | Waist circumference | Neale B | UKBB | European |
|  | Weight | Neale B | UKBB | European |
| rs6775319 | Arm/body/leg/trunk fat percentage | Neale B | UKBB | European |
| rs6895232 | / | / | / | / |
| rs564819152 | Invasive ovarian cancer | Phelan M | 28346442 | European |
| MVPA | | | | |
| rs429358 | Types of human blood cell traits | Astle W | 27863252 | European |
|  | Coronary artery disease | CARDIoGRAMplusC4D | 26343387 | Mixed |
|  | Myocardial infarction | CARDIoGRAMplusC4D | 26343387 | Mixed |
|  | Type II diabetes | DIAGRAM | 28566273 | European |
|  | Age-related macular degeneration | Fritsche LG | 26691988 | European |
|  | APOE apolipoprotein E, APOE apolipoprotein E females ,APOE apolipoprotein E males | Deelen J | 21418511 | European |
|  | Alzheimers disease | Potkin SG | 19668339 | Mixed |
|  | Alzheimers disease age of onset | Kamboh MI | 22005931 | European |
|  | Cognitive ageing | Davies G | 23207651 | European |
|  | Cognitive ageing females | Davies G | 23207651 | European |
|  | Cortical amyloid beta load | Ramanan VK | 23419831 | European |
|  | LDL cholesterol change with statins | Thompson | 20031582 | Mixed |
|  | Late onset Alzheimers disease | Naj AC | 20885792 | Unspecified |
|  | Late onset Alzheimers disease | Hu X | 21390209 | European |
|  | Late onset Alzheimers disease | Reitz C | 23571587 | African |
|  | Alzheimers disease | IGAP | 24162737 | European |
|  | Advanced age related macular degeneration | Fritsche LG | 26691988 | Mixed |
|  | Alzheimers disease biomarkers | Ramanan VK | 23419831 | European |
|  | Blood protein levels | Suhre K | 28240269 | European |
|  | Brain imaging | Shen | 20100581 | European |
|  | Cerebral amyloid deposition PET imaging | Li QS | 26252872 | European |
|  | Cerebral amyloid deposition positivity PET imaging | Li QS | 26252872 | European |
|  | Cerebrospinal AB1 42 levels in Alzheimers disease dementia | Ramirez A | 25027320 | European |
|  | Cognitive decline age related | Raj T | 28078323 | African |
|  | Dementia with Lewy bodies | Guerreiro R | 29263008 | European |
|  | HDL cholesterol | Surakka I | 25961943 | European |
|  | Lewy body disease | Beecham GW | 25188341 | Unspecified |
|  | Lifespan | Joshi PK | 27029810 | Mixed |
|  | Parental lifespan | Joshi PK | 29030599 | Mixed |
|  | Coronary artery disease | Nelson CP | 28714975 | Mixed |
|  | C-reactive protein | Prins B | 28887542 | European |
|  | Low density lipoprotein | Prins B | 28887542 | European |
|  | Total cholesterol | Prins B | 28887542 | European |
|  | Posterior cortical atrophy | Schott JM | 26993346 | Mixed |
|  | Coronary artery disease | van der Harst P | 29212778 | Mixed |
|  | Arm/Leg/trunk fat mass | Neale B | UKBB | European |
|  | Arm/body/leg/trunk fat percentage | Neale B | UKBB | European |
|  | Body mass index | Neale B | UKBB | European |
|  | Cause of death: alzheimers disease, unspecified | Neale B | UKBB | European |
|  | Cause of death: unspecified dementia | Neale B | UKBB | European |
|  | Chronic ischaemic heart disease | Neale B | UKBB | European |
|  | Fathers age at death | Neale B | UKBB | European |
|  | Frequency of stair climbing in last 4 weeks | Neale B | UKBB | European |
|  | Illnesses of father: alzheimers disease or dementia | Neale B | UKBB | European |
|  | Illnesses of father: diabetes | Neale B | UKBB | European |
|  | Illnesses of father: none of the above, group 1 | Neale B | UKBB | European |
|  | Illnesses of mother: alzheimers disease or dementia | Neale B | UKBB | European |
|  | Illnesses of mother: diabetes | Neale B | UKBB | European |
|  | Illnesses of mother: none of the above, group 1 | Neale B | UKBB | European |
|  | Illnesses of siblings: alzheimers disease or dementia | Neale B | UKBB | European |
|  | Medication for cholesterol, blood pressure or diabetes: cholesterol lowering medication | Neale B | UKBB | European |
|  | Medication for cholesterol, blood pressure or diabetes: none of the above | Neale B | UKBB | European |
|  | Mother still alive | Neale B | UKBB | European |
|  | Mothers age at death | Neale B | UKBB | European |
|  | No treatment with medication for cholesterol, blood pressure, diabetes, or take exogenous hormones | Neale B | UKBB | European |
|  | Number of days or week of moderate physical activity 10+ minutes | Neale B | UKBB | European |
|  | Pulse rate | Neale B | UKBB | European |
|  | Self-reported dementia or alzheimers/cognitive impairment | Neale B | UKBB | European |
|  | Self-reported high cholesterol | Neale B | UKBB | European |
|  | Treatment with atorvastatin | Neale B | UKBB | European |
|  | Treatment with cholesterol lowering medication | Neale B | UKBB | European |
|  | Treatment with ezetimibe | Neale B | UKBB | European |
|  | Treatment with ezetrol 10mg tablet | Neale B | UKBB | European |
|  | Treatment with lipitor 10mg tablet | Neale B | UKBB | European |
|  | Treatment with rosuvastatin | Neale B | UKBB | European |
|  | Treatment with simvastatin | Neale B | UKBB | European |
|  | Waist circumference | Neale B | UKBB | European |
|  | Weight | Neale B | UKBB | European |
|  | Weight change compared with 1 year ago | Neale B | UKBB | European |
|  | Whole body fat mass | Neale B | UKBB | European |
| rs2035562 | Arm/leg/trunk/body fat-free mass | Neale B | UKBB | European |
|  | Arm/leg /trunkpredicted mass | Neale B | UKBB | European |
|  | Basal metabolic rate | Neale B | UKBB | European |
|  | Impedance of arm left | Neale B | UKBB | European |
|  | Impedance of arm right | Neale B | UKBB | European |
|  | Impedance of whole body | Neale B | UKBB | European |
|  | Weight | Neale B | UKBB | European |
| rs149943 | Types of human blood cell traits | Astle W | 27863252 | European |
|  | IgA deficiency | Bronson P | 27723758 | European |
|  | Primary sclerosing cholangitis | Ji S | 27992413 | European |
|  | Schizophrenia | PGC | 25056061 | Mixed |
|  | Comparative body size at age 10 | Neale B | UKBB | European |
|  | Diastolic blood pressure | Neale B | UKBB | European |
|  | Doctor diagnosed sarcoidosis | Neale B | UKBB | European |
|  | Ever depressed for a whole week | Neale B | UKBB | European |
|  | Forced expiratory volume in 1-second | Neale B | UKBB | European |
|  | Forced expiratory volume in 1-second, best measure | Neale B | UKBB | European |
|  | Forced expiratory volume in 1-second, predicted percentage | Neale B | UKBB | European |
|  | Forced vital capacity | Neale B | UKBB | European |
|  | Heel bone mineral density | Neale B | UKBB | European |
|  | Intestinal malabsorption | Neale B | UKBB | European |
|  | Leg fat mass left | Neale B | UKBB | European |
|  | Leg fat mass right | Neale B | UKBB | European |
|  | Medication for pain relief, constipation, heartburn: none of the above | Neale B | UKBB | European |
|  | Medication for pain relief, constipation, heartburn: paracetamol | Neale B | UKBB | European |
|  | Mouth or teeth dental problems: dentures | Neale B | UKBB | European |
|  | Peak expiratory flow | Neale B | UKBB | European |
|  | Potassium in urine | Neale B | UKBB | European |
|  | Seen a psychiatrist for nerves, anxiety, tension or depression | Neale B | UKBB | European |
|  | Seen doctor for nerves, anxiety, tension or depression | Neale B | UKBB | European |
|  | Self-reported hyperthyroidism or thyrotoxicosis | Neale B | UKBB | European |
|  | Self-reported hypothyroidism or myxoedema | Neale B | UKBB | European |
|  | Self-reported malabsorption or coeliac disease | Neale B | UKBB | European |
|  | Self-reported sarcoidosis | Neale B | UKBB | European |
|  | Treatment with insulin product | Neale B | UKBB | European |
|  | Treatment with levothyroxine sodium | Neale B | UKBB | European |
|  | Unspecified haematuria | Neale B | UKBB | European |
|  | Weight | Neale B | UKBB | European |
| rs3094622 | Types of human blood cell traits | Astle W | 27863252 | European |
|  | IgA deficiency | Bronson P | 27723758 | European |
|  | Primary sclerosing cholangitis | Ji S | 27992413 | European |
|  | Arm fat-free mass left | Neale B | UKBB | European |
|  | Arm fat-free mass right | Neale B | UKBB | European |
|  | Arm predicted mass left | Neale B | UKBB | European |
|  | Arm predicted mass right | Neale B | UKBB | European |
|  | Asthma | Neale B | UKBB | European |
|  | Basal metabolic rate | Neale B | UKBB | European |
|  | Comparative body size at age 10 | Neale B | UKBB | European |
|  | Diastolic blood pressure | Neale B | UKBB | European |
|  | Doctor diagnosed sarcoidosis | Neale B | UKBB | European |
|  | Fluid intelligence score | Neale B | UKBB | European |
|  | Forced expiratory volume in 1-second | Neale B | UKBB | European |
|  | Forced expiratory volume in 1-second, predicted percentage | Neale B | UKBB | European |
|  | Height | Neale B | UKBB | European |
|  | Hip circumference | Neale B | UKBB | European |
|  | Intestinal malabsorption | Neale B | UKBB | European |
|  | Leg fat-free mass left | Neale B | UKBB | European |
|  | Leg fat-free mass right | Neale B | UKBB | European |
|  | Leg predicted mass left | Neale B | UKBB | European |
|  | Leg predicted mass right | Neale B | UKBB | European |
|  | Medication for cholesterol, blood pressure or diabetes: insulin | Neale B | UKBB | European |
|  | Medication for pain relief, constipation, heartburn: none of the above | Neale B | UKBB | European |
|  | Medication for pain relief, constipation, heartburn: paracetamol | Neale B | UKBB | European |
|  | Mouth or teeth dental problems: dentures | Neale B | UKBB | European |
|  | Number of days or week of moderate physical activity 10+ minutes | Neale B | UKBB | European |
|  | Peak expiratory flow | Neale B | UKBB | European |
|  | Potassium in urine | Neale B | UKBB | European |
|  | Seen a psychiatrist for nerves, anxiety, tension or depression | Neale B | UKBB | European |
|  | Seen doctor for nerves, anxiety, tension or depression | Neale B | UKBB | European |
|  | Self-reported adrenocortical insufficiency or addisons disease | Neale B | UKBB | European |
|  | Self-reported asthma | Neale B | UKBB | European |
|  | Self-reported enlarged prostate | Neale B | UKBB | European |
|  | Self-reported hyperthyroidism or thyrotoxicosis | Neale B | UKBB | European |
|  | Self-reported hypothyroidism or myxoedema | Neale B | UKBB | European |
|  | Self-reported malabsorption or coeliac disease | Neale B | UKBB | European |
|  | Self-reported psoriasis | Neale B | UKBB | European |
|  | Self-reported sarcoidosis | Neale B | UKBB | European |
|  | Self-reported systemic lupus erythematosis or sle | Neale B | UKBB | European |
|  | Sitting height | Neale B | UKBB | European |
|  | Started insulin within one-year diagnosis of diabetes | Neale B | UKBB | European |
|  | Treatment with carbimazole | Neale B | UKBB | European |
|  | Treatment with fludrocortisone | Neale B | UKBB | European |
|  | Treatment with insulin | Neale B | UKBB | European |
|  | Treatment with insulin product | Neale B | UKBB | European |
|  | Treatment with levothyroxine sodium | Neale B | UKBB | European |
|  | Trunk fat-free mass | Neale B | UKBB | European |
|  | Trunk predicted mass | Neale B | UKBB | European |
|  | Unspecified haematuria | Neale B | UKBB | European |
|  | Weight | Neale B | UKBB | European |
|  | Whole body fat-free mass | Neale B | UKBB | European |
|  | Whole body water mass | Neale B | UKBB | European |
| rs2854277 | Types of human blood cell traits | Astle W | 27863252 | European |
|  | IgA deficiency | Bronson P | 27723758 | European |
|  | Age-related macular degeneration | Fritsche LG | 26691988 | European |
|  | Rheumatoid arthritis | Okada Y | 24390342 | European |
|  | Rheumatoid arthritis | Okada Y | 24390342 | Mixed |
|  | Schizophrenia | PGC | 25056061 | Mixed |
| rs7804463 | Number of days or week of vigorous physical activity 10+ minutes | Neale B | UKBB | European |
| rs7791992 | Types of human blood cell traits | Astle W | 27863252 | European |
| rs2988004 | / | / | / | / |
| Sedentary time | | | | |
| rs26579 | Trunk fat percentage | Neale B | UKBB | European |
| rs25981 | / | / | / | / |
| rs1858242 | / | / | / | / |
| rs34858520 | / | / | / | / |
| rs6870096 | / | / | / | / |
| rs61776614 | / | / | / | / |
| PA: Physical activity, MVPA: self-reported moderate-to-vigorous physical activity, AMPA: acceleration vector magnitude physical activity, *: the two SNPs are in linkage disequilibrium. | | | | |

| Table S4 Effect sizes can be detected with the power of 0.8 given the sample size, proportion of cases and variance explained by instrumental variable for each Mendelian randomisation analysis | | | | | | |
| --- | --- | --- | --- | --- | --- | --- |
| Exposure | Sample size of exposure GWAS | Variance explained by instrument (F statistic) | Outcome | Sample size of outcome GWAS | Proportion of cases in outcome GWAS | Power=0.8 |
| **Two-sample MR** |  |  |  |  |  |  |
| Self-reported MVPA | 385,790 | 0.0007 (273) | CRC (without UKBB data) * | 92,967 (67,878) * | 0.34 (0.39) * | OR=0.44 (OR=0.40) * |
| AMPA (5 SNP at P<5×10^-8^**^)^** | 91,105 | 0.002 (163) | CRC (without UKBB data) * | 92,967 (67,878) * | 0.34 (0.39) * | OR=0.63 (OR=0.60) * |
| AMPA (3 SNP at P<5×10^-9^**^)^** | 91,105 | 0.001 (106) | CRC (without UKBB data) * | 92,967 (67,878) * | 0.34 (0.39) * | OR=0.51 (OR=0.48) * |
| Sedentary time | 91,105 | 0.001 (106) | CRC (without UKBB data) * | 92,967 (67,878) * | 0.34 (0.39) * | OR=0.51 (OR=0.48) * |
| BMI | 322,154 | 0.0232 (7300) | CRC (without UKBB data) * | 92,967 (67,878) * | 0.34 (0.39) * | OR=1.13 (OR=1.15) * |
| Body fat percentage | 331,117 | 0·053 (5186) | CRC (without UKBB data) * | 92,967 (67,878) * | 0.34 (0.39) * | OR=1.09 (OR=1.10) * |
| Waist circumference | 224,459 | 0.010 (939) | CRC (without UKBB data) * | 92,967 (67,878) * | 0.34 (0.39) * | OR=1.21 (OR=1.24) * |
| AFR | 362,499 | 0.002 (725) | CRC | 92,967 | 0.34 | OR=1.59 |
| TFR | 362,499 | 0.002 (725) | CRC | 92,967 | 0.34 | OR=1.59 |
| LFR | 362,499 | 0.002 (725) | CRC | 92,967 | 0.34 | OR=1.59 |
| **Network MR** |  |  |  |  |  |  |
| Self-reported MVPA | 385,790 | 0.0007 (273) | BMI | 322,154 | / | OR=0.42 |
| AMPA (5 SNP at P<5×10^-8^**^)^** | 91,105 | 0.002 (163) | BMI | 322,154 | / | OR=0.60 |
| AMPA (3 SNP at P<5×10^-9^**^)^** | 91,105 | 0.001 (106) | BMI | 322,154 | / | OR=0.49 |
| BMI | 322,154 | 0.0232 (7300) | CRC (without UKBB data) * | 92,967 (67,878) * | 0.34 (0.39) * | OR=1.13 (OR=1.15) * |
| GWAS: genome-wide association study, MR: mendelian randomisation, PA: physical activity, MVPA: self-reported moderate-to-vigorous physical activity, AMPA: acceleration vector magnitude physical activity, UKBB: UK Biobank, CRC: colorectal cancer, BMI: body mass index, OR: odds ratio, *: results after excluding UKBB data from the analysis were presented in the round bracket, AFR: arm fat ratio, LFR: leg fat ratio, TFR: trunk fat ratio. | | | | | | |

| Table S5 Results of Two-sample Mendelian randomisation analyses of physical activity on colorectal cancer risk | | | | | | | | | | | | | | | | | | | |
| --- | --- | --- | --- | --- | --- | --- | --- | --- | --- | --- | --- | --- | --- | --- | --- | --- | --- | --- | --- |
|  | ^#^**IV includes 7 MVPA SNPs at P<5×10^-9^** | | | | | **IV includes 5 AMPA SNPs at P<5×10^-8^** | | | | | **IV includes 3 AMPA SNPs at P<5×10^-9^** | | | | | **IV includes 6 sedentary time SNPs at P<5×10^-8^** | | | |
|  | With UK Biobank data (P_int_^a^=0.75, P_het_^b^=0.56) | | *Without UK Biobank data (P_int_^a^=0.85, P_het_^b^=0.89) | | With UK Biobank data (Pinta=0.12, P_het_^b^=0.34) | | | *Without UK Biobank data (P_int_^a^=0.09, P_het_^b^=0.40) | | With UK Biobank data (P_int_^a^=0.16, P_het_^b^=0.35) | | | *Without UK Biobank data (P_int_^a^=0.13, P_het_^b^=0.32) | | With UK Biobank data (P_int_^a^=0.18, P_het_^b^=0.40) | | | *Without UK Biobank data (P_int_^a^=0.33, P_het_^b^=0.79) | |
|  | Causal effects (95%CI) | P | Causal effects (95%CI) | P | Causal effects (95%CI) | | P | Causal effects (95%CI) | P | Causal effects (95%CI) | | P | Causal effects (95%CI) | P | Causal effects (95%CI) | | P | Causal effects (95%CI) | P |
| **Main analysis** | | | | | | | | | | | | | | | | | | | |
| IVW | 0.56 (0.31,1.01) | 0.05 | 0.54 (0.24,1.20) | 0.13 | 0.60 (0.41,0.88) | | 0.008 | 0.46 (0.30,0.72) | 0.0009 | 0.54 (0.35, 0.86) | | 0.009 | 0.41 (0.23, 0.72) | 0.002 | 0.80 (0.57,1.12) | | 0.19 | 0.85 (0.57,1.29) | 0.45 |
| **Sensitivity analysis** | | | | | | | | | | | | | | | | | | | |
| MBE | 0.49 (0.17,1.45) | 0.2 | 0.73 (0.17, 3.06) | 0.67 | 0.61 (0.30,1.24) | | 0.18 | 0.53 (0.22,1.28) | 0.16 | 0.69 (0.32, 1.46) | | 0.32 | 0.58 (0.22,1.53) | 0.28 | 1.09 (0.55,2.16) | | 0.81 | 0.91 (0.47,1.76) | 0.77 |
| Weighted median | 0.57 (0.25,1.31) | 0.18 | 0.53 (0.18,1.51) | 0.23 | 0.59 (0.36,0.95) | | 0.03 | 0.49 (0.27,0.89) | 0.02 | 0.59 (0.33, 1.03) | | 0.06 | 0.47 (0.24,0.90) | 0.02 | 0.85 (0.56,1.30) | | 0.46 | 0.92 (0.55,1.51) | 0.73 |
| MR-Egger | 0.39 (0.04,3.85) | 0.42 | 0.87 (0.01,160.25) | 0.96 | 0.05 (0.002,1.10) | | 0.06 | 0.02 (0.0006,0.75) | 0.03 | 0.05 (0.002, 1.46) | | 0.08 | 0.02 (0.0003,1.06) | 0.05 | 3.25 (0.41,25.85) | | 0.27 | 2.85 (0.24,33.09) | 0.40 |
| MR-Robust | 0.56 (0.31,1.01) | 0.05 | 0.54 (0.24,1.20) | 0.13 | 0.60 (0.41,0.86) | | 0.006 | 0.47 (0.28,0.72) | 0.003 | 0.55 (0.41, 0.73) | | 3.18E-05 | 0.41 (0.24,0.72) | 0.002 | 0.80 (0.58,1.11) | | 0.18 | 0.86 (0.64,1.15) | 0.30 |
| MR-PRESSO | 0.52 (0.29,0.93) | 0.03 | 0.54 (0.24,1.20) | 0.13 | 0.60 (0.41,0.88) | | 0.008 | 0.46 (0.30,0.72) | 0.0009 | / | | / | / | / | 0.80 (0.57,1.12) | | 0.19 | 0.85 (0.57,1.29) | 0.45 |
| MVPA: self-reported moderate-to-vigorous physical activity, AMPA: acceleration vector magnitude physical activity, CI: confidence interval, P_int_^a^: P-value for the intercept of MR-Egger’s test, P_het_^b^: P-values of χ2 Q test for heterogeneity, IVW: inverse variance-weighted, MBE: mode-based estimate, MR-PRESSO: MR–Pleiotropy Residual Sum and Outlier, *: UK Biobank samples were removed from the meta-analysis of 15 primary CRC GWAS, ^#^: correlation matrix was added in the analyses of IVW, MR-Egger and MR-Robust methods, rs149943 was removed because of linkage disequilibrium (r^2^=0.35) for median-based method. | | | | | | | | | | | | | | | | | | | |

| Table S6 Summary of associations between genetic variants and physical activity and colorectal cancer risk | | | | | | | | | | | |
| --- | --- | --- | --- | --- | --- | --- | --- | --- | --- | --- | --- |
| **Category** | **Chr** | **Position** | **rsid** | **EA for PA/CRC** | **Beta.PA** | **SE.PA** | **Beta.CRC** | **SE.CRC** | ***Beta.CRC** | ***SE.CRC** |  |
| MVPA | 3 | 85,056,521 | rs2035562 | A (G) | -0.014 | 0.004 | 0.012 | 0.011 | 0.021 | 0.013 |  |
| MVPA | 6 | 28,002,388 | rs149943 | G (A) | 0.019 | 0.005 | -0.018 | 0.016 | -0.006 | 0.021 |  |
| MVPA | 6 | 30,327,952 | rs3094622 | A (G) | 0.020 | 0.005 | -0.041 | 0.018 | -0.026 | 0.027 |  |
| MVPA | 6 | 32,628,084 | rs2854277 | C (T) | 0.032 | 0.008 | -0.014 | 0.020 | -0.010 | 0.033 |  |
| MVPA | 7 | 133,447,651 | rs7804463 | T (C) | 0.015 | 0.003 | 0.006 | 0.011 | 0.003 | 0.016 |  |
| MVPA | 7 | 50,237,784 | rs7791992 | C (A) | -0.014 | 0.004 | 0.013 | 0.011 | 0.012 | 0.013 |  |
| MVPA | 9 | 37,044,388 | rs2988004 | T (G) | -0.013 | 0.004 | 0.003 | 0.011 | 0.003 | 0.014 |  |
| AMPA | 17 | 44,326,864 | rs2696625 | A (G) | -0.037 | 0.005 | 0.037 | 0.013 | 0.050 | 0.015 |  |
| AMPA | 18 | 40,768,309 | rs59499656 | A (T) | -0.028 | 0.005 | 0.007 | 0.011 | 0.014 | 0.016 |  |
| AMPA | 5 | 152,039,421 | rs6895232 | T (A) | 0.027 | 0.005 | 0.005 | 0.013 | 0.003 | 0.019 |  |
| AMPA | 3 | 18,758,501 | rs6775319 | A (T) | 0.027 | 0.005 | -0.020 | 0.012 | -0.024 | 0.016 |  |
| AMPA | 10 | 21,820,650 | rs564819152 | A (G) | 0.028 | 0.005 | -0.013 | 0.011 | -0.016 | 0.013 |  |
| Sedentary time | 5 | 87,985,295 | rs26579 | G (C) | 0.028 | 0.005 | 0.003 | 0.011 | -0.002 | 0.012 |  |
| Sedentary time | 5 | 106,822,908 | rs25981 | G (C) | 0.028 | 0.005 | -0.017 | 0.011 | -0.013 | 0.018 |  |
| Sedentary time | 3 | 68,527,135 | rs1858242 | A (G) | 0.031 | 0.005 | 0.003 | 0.012 | -0.002 | 0.014 |  |
| Sedentary time | 7 | 71,723,883 | rs34858520 | A (G) | 0.028 | 0.005 | -0.023 | 0.013 | -0.018 | 0.016 |  |
| Sedentary time | 5 | 151,945,811 | rs6870096 | C (G) | -0.028 | 0.005 | 0.011 | 0.012 | 0.006 | 0.014 |  |
| Sedentary time | 1 | 2,166,406 | rs61776614 | C (T) | 0.050 | 0.009 | 0.012 | 0.023 | 0.014 | 0.027 |  |
| PA: physical activity, BMI: body mass index, CRC: colorectal cancer, MVPA: self-reported moderate-to-vigorous physical activity, AMPA: acceleration vector magnitude physical activity, EA: effect allele, EAF: effect allele frequency, SE: standard error  Associations between genetic variants and MVPA were extracted from Klimentidis et al.,^4^ Association between genetic variants and AMPA and sedentary time were extracted from Doherty et al.^15^, Associations between PA genetic variants and CRC risk were extracted from a meta-analysis of 15 primary CRC GWAS.^1^ Associations between genetic variants and BMI were extracted from a meta-analysis of anthropometric traits GWAS study,^6^ The Proxy of BMI was selected in linkage disequilibrium (r^2^>0.8), *: UK Biobank samples were removed from the meta-analysis of 15 primary CRC GWAS. | | | | | | | | | | | |

| Table S7 Genetic variants associated with body mass index recorded by Locke (2015) | | | | | | | | |
| --- | --- | --- | --- | --- | --- | --- | --- | --- |
| rsid | Chr | Position | EA | EAF | Beta.BMI | SE.BMI | Beta.CRC | SE.CRC |
| rs1000940 | 17 | 5283252 | G | 0.32 | 0.019 | 0.003 | -0.011 | 0.012 |
| rs10132280 | 14 | 25928179 | C | 0.68 | 0.023 | 0.003 | 0.008 | 0.012 |
| rs1016287 | 2 | 59305625 | T | 0.29 | 0.023 | 0.003 | 0.007 | 0.012 |
| rs10733682 | 9 | 129460914 | A | 0.48 | 0.017 | 0.003 | 0.008 | 0.011 |
| rs10938397 | 4 | 45182527 | G | 0.43 | 0.04 | 0.003 | 0.011 | 0.011 |
| rs10968576 | 9 | 28414339 | G | 0.32 | 0.025 | 0.003 | 0.02 | 0.011 |
| rs11030104 | 11 | 27684517 | A | 0.79 | 0.041 | 0.004 | 0.053 | 0.013 |
| rs11057405 | 12 | 122781897 | G | 0.9 | 0.031 | 0.006 | -0.027 | 0.018 |
| rs11126666 | 2 | 26928811 | A | 0.28 | 0.021 | 0.003 | -0.011 | 0.012 |
| rs11165643 | 1 | 96924097 | T | 0.58 | 0.022 | 0.003 | 0.017 | 0.011 |
| rs11191560 | 10 | 104869038 | C | 0.09 | 0.031 | 0.005 | 0.032 | 0.02 |
| rs1167827 | 7 | 75163169 | G | 0.55 | 0.02 | 0.003 | 0.005 | 0.011 |
| rs11688816 | 2 | 63053048 | G | 0.53 | 0.017 | 0.003 | 0.002 | 0.011 |
| rs11847697 | 14 | 30515112 | T | 0.04 | 0.049 | 0.008 | 0.009 | 0.027 |
| rs12286929 | 11 | 115022404 | G | 0.52 | 0.022 | 0.003 | 0.01 | 0.011 |
| rs12401738 | 1 | 78446761 | A | 0.35 | 0.021 | 0.003 | 0 | 0.011 |
| rs12429545 | 13 | 54102206 | A | 0.13 | 0.033 | 0.05 | 0.016 | 0.016 |
| rs12446632 | 16 | 19935389 | G | 0.87 | 0.04 | 0.005 | 0.005 | 0.015 |
| rs12566985 | 1 | 75002193 | G | 0.45 | 0.024 | 0.003 | -0.018 | 0.011 |
| rs12940622 | 17 | 78615571 | G | 0.58 | 0.018 | 0.003 | -0.007 | 0.011 |
| rs13021737 | 2 | 632348 | G | 0.83 | 0.06 | 0.004 | 0.018 | 0.014 |
| rs13107325 | 4 | 103188709 | T | 0.07 | 0.048 | 0.007 | 0.039 | 0.02 |
| rs13191362 | 6 | 163033350 | A | 0.88 | 0.028 | 0.005 | 0.009 | 0.017 |
| rs1516725 | 3 | 185824004 | C | 0.87 | 0.045 | 0.005 | -0.006 | 0.016 |
| rs1528435 | 2 | 181550962 | T | 0.63 | 0.018 | 0.003 | -0.001 | 0.011 |
| rs1558902 | 16 | 53803574 | A | 0.42 | 0.082 | 0.003 | -0.001 | 0.011 |
| rs16851483 | 3 | 141275436 | T | 0.07 | 0.048 | 0.008 | 0.011 | 0.022 |
| rs17001654 | 4 | 77129568 | G | 0.15 | 0.031 | 0.005 | 0.035 | 0.015 |
| rs17024393 | 1 | 110154688 | C | 0.04 | 0.066 | 0.009 | 0.009 | 0.033 |
| rs17094222 | 10 | 102395440 | C | 0.21 | 0.025 | 0.004 | 0.013 | 0.014 |
| rs17405819 | 8 | 76806584 | T | 0.7 | 0.022 | 0.003 | -0.002 | 0.012 |
| rs17724992 | 19 | 18454825 | A | 0.75 | 0.019 | 0.004 | -0.008 | 0.012 |
| rs1808579 | 18 | 21104888 | C | 0.53 | 0.017 | 0.003 | 0.002 | 0.011 |
| rs1928295 | 9 | 120378483 | T | 0.55 | 0.019 | 0.003 | 0.013 | 0.011 |
| rs2033529 | 6 | 40348653 | G | 0.29 | 0.019 | 0.003 | 0.009 | 0.012 |
| rs2033732 | 8 | 85079709 | C | 0.75 | 0.019 | 0.004 | -0.015 | 0.013 |
| rs205262 | 6 | 34563164 | G | 0.27 | 0.022 | 0.004 | -0.011 | 0.012 |
| rs2112347 | 5 | 75015242 | T | 0.63 | 0.026 | 0.003 | 0.011 | 0.011 |
| rs2121279 | 2 | 143043285 | T | 0.15 | 0.025 | 0.004 | 0.038 | 0.016 |
| rs2176598 | 11 | 43864278 | T | 0.25 | 0.02 | 0.004 | 0.014 | 0.012 |
| rs2207139 | 6 | 50845490 | G | 0.18 | 0.045 | 0.004 | 0.006 | 0.014 |
| rs2245368 | 7 | 76608143 | C | 0.18 | 0.032 | 0.006 | 0.008 | 0.019 |
| rs2287019 | 19 | 46202172 | C | 0.8 | 0.036 | 0.004 | -0.005 | 0.014 |
| rs2365389 | 3 | 61236462 | C | 0.58 | 0.02 | 0.003 | 0.024 | 0.011 |
| rs2780337 | 13 | 28007830 | T | 0.2 | 0.03 | 0.005 | 0.015 | 0.011 |
| rs29941 | 19 | 34309532 | G | 0.67 | 0.018 | 0.003 | -0.003 | 0.012 |
| rs3101336 | 1 | 72751185 | C | 0.61 | 0.033 | 0.003 | 0.005 | 0.011 |
| rs3736485 | 15 | 51748610 | A | 0.45 | 0.018 | 0.003 | 0.026 | 0.011 |
| rs3810291 | 19 | 47569003 | A | 0.67 | 0.028 | 0.004 | 0.003 | 0.012 |
| rs3817334 | 11 | 47650993 | T | 0.41 | 0.026 | 0.003 | -0.003 | 0.011 |
| rs3849570 | 3 | 81792112 | A | 0.36 | 0.019 | 0.003 | 0.016 | 0.011 |
| rs3888190 | 16 | 28889486 | A | 0.4 | 0.031 | 0.003 | -0.006 | 0.011 |
| rs4256980 | 11 | 8673939 | G | 0.65 | 0.021 | 0.003 | 0.004 | 0.011 |
| rs4740619 | 9 | 15634326 | T | 0.54 | 0.018 | 0.003 | 0.012 | 0.011 |
| rs543874 | 1 | 177889480 | G | 0.19 | 0.048 | 0.004 | -0.011 | 0.013 |
| rs6477694 | 9 | 111932342 | C | 0.37 | 0.017 | 0.003 | 0.012 | 0.012 |
| rs6567160 | 18 | 57829135 | C | 0.24 | 0.056 | 0.004 | 0.022 | 0.013 |
| rs657452 | 1 | 49589847 | A | 0.39 | 0.023 | 0.003 | 0.005 | 0.011 |
| rs6804842 | 3 | 25106437 | G | 0.58 | 0.019 | 0.003 | -0.012 | 0.011 |
| rs7138803 | 12 | 50247468 | A | 0.38 | 0.032 | 0.003 | 0.002 | 0.011 |
| rs7141420 | 14 | 79899454 | T | 0.53 | 0.024 | 0.003 | 0.021 | 0.011 |
| rs7243357 | 18 | 56883319 | T | 0.81 | 0.022 | 0.004 | 0.01 | 0.014 |
| rs758747 | 16 | 3627358 | T | 0.27 | 0.023 | 0.004 | 0.002 | 0.012 |
| rs7599312 | 2 | 213413231 | G | 0.72 | 0.022 | 0.003 | 0.005 | 0.012 |
| rs7899106 | 10 | 87410904 | G | 0.05 | 0.04 | 0.007 | -0.026 | 0.025 |
| rs7903146 | 10 | 114758349 | C | 0.71 | 0.023 | 0.003 | -0.006 | 0.012 |
| rs9400239 | 6 | 108977663 | C | 0.69 | 0.019 | 0.003 | 0.015 | 0.012 |
| rs9925964 | 16 | 31129895 | A | 0.62 | 0.019 | 0.003 | 0.024 | 0.011 |
| BMI: body mass index, CRC: colorectal cancer, EA: effect allele, EAF: effect allele frequency, associations between genetic variants and BMI were extracted from Locke (2015)., associations between BMI genetic variants and CRC risk were extracted from a meta-analysis of 15 primary CRC GWAS (Law 2019). | | | | | | | | |

| Table S8 Genetic variants associated with body fat percentage recorded by Bycroft (2018) | | | | | | | | | | | | |
| --- | --- | --- | --- | --- | --- | --- | --- | --- | --- | --- | --- | --- |
| SNP | Chr | Position | EA | EAF | Beta.BFP | SE.BFP | P.BFP | Beta.CRC | SE.CRC | P.CRC | P.CRC |  |
| rs10100245 | 8 | 76314684 | A | 0.557 | 0.019 | 0.002 | 1.93E-14 | 0.001 | 0.012 | 9.51E-01 | 5.97E-01 |  |
| rs1013293 | 1 | 62104649 | G | 0.428 | -0.018 | 0.002 | 3.24E-13 | 0.021 | 0.012 | 9.44E-02 | 3.10E-01 |  |
| rs10172196 | 2 | 36553406 | A | 0.308 | 0.017 | 0.003 | 2.45E-10 | 0.011 | 0.013 | 4.26E-01 | 6.34E-01 |  |
| rs10259620 | 7 | 27162670 | A | 0.777 | -0.021 | 0.003 | 6.24E-12 | -0.012 | 0.015 | 4.20E-01 | 9.21E-01 |  |
| rs10278040 | 7 | 99543750 | G | 0.069 | -0.031 | 0.005 | 1.31E-09 | -0.007 | 0.026 | 7.87E-01 | 4.82E-01 |  |
| rs1038088 | 17 | 29747545 | G | 0.513 | 0.016 | 0.002 | 2.53E-10 | -0.025 | 0.012 | 3.78E-02 | 5.75E-01 |  |
| rs10423928 | 19 | 45679046 | T | 0.206 | -0.030 | 0.003 | 7.47E-22 | -0.005 | 0.016 | 7.38E-01 | 3.00E-01 |  |
| rs1046080 | 6 | 31628105 | A | 0.734 | 0.024 | 0.003 | 2.94E-18 | 0.057 | 0.014 | 7.68E-05 | 3.26E-01 |  |
| rs10499014 | 6 | 97499879 | C | 0.268 | -0.018 | 0.003 | 1.61E-10 | -0.003 | 0.014 | 8.57E-01 | 7.47E-01 |  |
| rs10510025 | 10 | 116891485 | T | 0.246 | 0.018 | 0.003 | 1.77E-10 | -0.016 | 0.014 | 2.47E-01 | 8.23E-01 |  |
| rs10514641 | 2 | 41321639 | G | 0.315 | 0.017 | 0.003 | 4.45E-10 | -0.003 | 0.015 | 8.21E-01 | 3.78E-01 |  |
| rs1056441 | 20 | 63738996 | C | 0.685 | 0.018 | 0.003 | 8.69E-12 | -0.039 | 0.013 | 3.21E-03 | 9.07E-01 |  |
| rs10732335 |  |  | A | 0.447 | -0.028 | 0.002 | 4.84E-29 | -0.018 | 0.012 | 1.40E-01 | 6.86E-01 |  |
| rs10756798 | 9 | 16739765 | C | 0.645 | -0.020 | 0.003 | 2.65E-15 | -0.001 | 0.015 | 9.40E-01 | 8.12E-01 |  |
| rs10758031 | 9 | 31174648 | A | 0.749 | -0.016 | 0.003 | 4.50E-09 | -0.022 | 0.014 | 1.20E-01 | 7.81E-01 |  |
| rs10766077 | 11 | 13328234 | G | 0.388 | -0.016 | 0.003 | 2.99E-10 | 0.015 | 0.013 | 2.30E-01 | 3.25E-01 |  |
| rs10774018 | 12 | 2048759 | C | 0.234 | 0.017 | 0.003 | 1.81E-08 | 0.024 | 0.015 | 9.65E-02 | 7.87E-01 |  |
| rs10791109 | 11 | 130980482 | G | 0.506 | 0.016 | 0.002 | 9.55E-11 | 0.000 | 0.012 | 9.71E-01 | 5.00E-01 |  |
| rs10803762 | 2 | 160249365 | A | 0.685 | 0.016 | 0.003 | 2.24E-09 | -0.005 | 0.013 | 7.17E-01 | 7.38E-01 |  |
| rs10830566 | 11 | 90740789 | A | 0.273 | -0.015 | 0.003 | 2.04E-08 | -0.022 | 0.014 | 1.11E-01 | 8.46E-01 |  |
| rs10848835 | 12 | 3232344 | G | 0.093 | -0.029 | 0.004 | 3.09E-12 | -0.052 | 0.022 | 1.59E-02 | 3.78E-01 |  |
| rs10854853 | 22 | 48478600 | T | 0.450 | 0.014 | 0.002 | 3.01E-08 | 0.024 | 0.015 | 1.23E-01 | 1.59E-02 |  |
| rs10938397 | 4 | 45180510 | G | 0.439 | 0.026 | 0.002 | 5.59E-25 | 0.017 | 0.012 | 1.58E-01 | 7.48E-01 |  |
| rs10947510 | 6 | 34563523 | T | 0.470 | -0.014 | 0.002 | 4.69E-09 | -0.032 | 0.013 | 1.22E-02 | 1.40E-01 |  |
| rs10947793 | 6 | 12142584 | A | 0.375 | -0.015 | 0.003 | 1.38E-09 | 0.010 | 0.013 | 4.33E-01 | 5.99E-01 |  |
| rs10954772 | 8 | 31006422 | T | 0.687 | -0.018 | 0.003 | 2.82E-11 | -0.004 | 0.013 | 7.59E-01 | 2.81E-04 |  |
| rs10999460 | 10 | 70668527 | T | 0.270 | 0.022 | 0.003 | 2.01E-15 | -0.007 | 0.014 | 5.95E-01 | 9.30E-01 |  |
| rs11012732 | 10 | 21541175 | G | 0.333 | 0.024 | 0.003 | 1.04E-19 | 0.012 | 0.013 | 3.39E-01 | 3.11E-02 |  |
| rs11030016 | 11 | 27466445 | T | 0.731 | 0.016 | 0.003 | 1.93E-08 | 0.039 | 0.014 | 4.66E-03 | 6.25E-01 |  |
| rs11042030 | 11 | 8669171 | T | 0.279 | -0.018 | 0.003 | 4.24E-11 | -0.009 | 0.014 | 4.96E-01 | 3.67E-01 |  |
| rs11113445 | 12 | 107694905 | G | 0.398 | 0.019 | 0.003 | 1.34E-13 | 0.027 | 0.012 | 3.35E-02 | 1.07E-01 |  |
| rs11119364 | 1 | 209904236 | T | 0.166 | 0.021 | 0.003 | 4.35E-11 | 0.013 | 0.016 | 4.37E-01 | 6.71E-01 |  |
| rs11150745 | 17 | 80783826 | A | 0.310 | -0.017 | 0.003 | 1.22E-10 | 0.007 | 0.013 | 5.97E-01 | 5.89E-02 |  |
| rs111743285 | 6 | 126727085 | T | 0.235 | 0.021 | 0.003 | 3.32E-12 | 0.046 | 0.015 | 1.67E-03 | 5.32E-02 |  |
| rs11258438 | 10 | 13528312 | A | 0.550 | 0.013 | 0.002 | 4.58E-08 | 0.014 | 0.012 | 2.53E-01 | 1.53E-01 |  |
| rs113019802 | 2 | 46657685 | G | 0.212 | -0.019 | 0.003 | 1.98E-10 | -0.017 | 0.015 | 2.66E-01 | 1.17E-01 |  |
| rs113230003 | 19 | 18350146 | G | 0.256 | -0.020 | 0.003 | 2.01E-12 | 0.002 | 0.014 | 8.82E-01 | 5.02E-01 |  |
| rs114052060 | 1 | 150153170 | A | 0.058 | -0.038 | 0.005 | 5.10E-13 | -0.040 | 0.041 | 3.26E-01 | 3.33E-03 |  |
| rs114295766 | 3 | 99637517 | A | 0.063 | -0.027 | 0.005 | 2.68E-08 | -0.009 | 0.097 | 9.30E-01 | 6.27E-01 |  |
| rs11594905 | 10 | 75899975 | A | 0.119 | 0.020 | 0.004 | 1.99E-08 | -0.016 | 0.023 | 5.02E-01 | 8.19E-01 |  |
| rs11603783 | 11 | 122882246 | C | 0.242 | 0.017 | 0.003 | 4.66E-09 | -0.005 | 0.014 | 7.47E-01 | 2.24E-01 |  |
| rs116298073 | 3 | 84046615 | C | 0.060 | -0.033 | 0.005 | 1.48E-10 | 0.007 | 0.026 | 7.81E-01 | 6.77E-01 |  |
| rs116399833 | 19 | 18391564 | A | 0.228 | 0.017 | 0.003 | 1.15E-08 | -0.014 | 0.017 | 4.02E-01 | 7.89E-01 |  |
| rs11642015 | 16 | 53768582 | T | 0.410 | 0.052 | 0.003 | 3.10E-96 | 0.005 | 0.012 | 7.04E-01 | 7.55E-01 |  |
| rs116792274 | 1 | 45699867 | C | 0.033 | -0.035 | 0.006 | 3.25E-08 | 0.008 | 0.034 | 8.23E-01 | 3.41E-01 |  |
| rs11685627 | 2 | 120713470 | G | 0.478 | -0.014 | 0.002 | 3.50E-08 | -0.001 | 0.015 | 9.49E-01 | 3.35E-02 |  |
| rs11695700 | 2 | 109391866 | T | 0.424 | 0.014 | 0.002 | 2.35E-08 | -0.009 | 0.012 | 4.75E-01 | 9.44E-01 |  |
| rs117068593 | 14 | 92651884 | C | 0.185 | -0.019 | 0.003 | 1.36E-09 | -0.022 | 0.016 | 1.67E-01 | 1.80E-02 |  |
| rs11742930 | 5 | 106438397 | T | 0.558 | 0.014 | 0.002 | 7.75E-09 | -0.006 | 0.012 | 6.39E-01 | 9.44E-01 |  |
| rs117632017 | 15 | 51967910 | A | 0.031 | 0.036 | 0.006 | 1.04E-08 | 0.000 | 0.056 | 9.95E-01 | 3.39E-01 |  |
| rs11781222 | 8 | 23532058 | T | 0.129 | -0.021 | 0.004 | 5.91E-09 | -0.053 | 0.021 | 1.08E-02 | 4.38E-01 |  |
| rs11782341 | 8 | 4955937 | G | 0.187 | 0.020 | 0.003 | 4.58E-10 | 0.005 | 0.019 | 8.07E-01 | 8.53E-02 |  |
| rs11786089 | 8 | 22118008 | G | 0.450 | 0.015 | 0.002 | 3.42E-10 | 0.000 | 0.013 | 9.71E-01 | 8.51E-01 |  |
| rs11790018 | 9 | 126940563 | C | 0.399 | -0.014 | 0.003 | 3.22E-08 | 0.027 | 0.013 | 3.53E-02 | 1.32E-01 |  |
| rs11810577 | 1 | 109595487 | T | 0.189 | 0.018 | 0.003 | 8.75E-09 | 0.009 | 0.016 | 5.67E-01 | 3.58E-02 |  |
| rs1182143 | 7 | 2873294 | T | 0.298 | -0.016 | 0.003 | 1.90E-09 | -0.036 | 0.014 | 9.68E-03 | 1.04E-02 |  |
| rs11852419 | 15 | 41164176 | T | 0.263 | 0.017 | 0.003 | 3.45E-09 | -0.002 | 0.014 | 8.98E-01 | 4.47E-01 |  |
| rs11856579 | 15 | 77720346 | G | 0.254 | -0.017 | 0.003 | 2.14E-09 | -0.017 | 0.015 | 2.53E-01 | 3.66E-01 |  |
| rs11857221 | 15 | 47457237 | A | 0.399 | 0.014 | 0.003 | 1.59E-08 | 0.025 | 0.012 | 4.34E-02 | 2.86E-01 |  |
| rs11866219 | 16 | 69515846 | A | 0.590 | -0.022 | 0.002 | 7.41E-19 | 0.030 | 0.013 | 1.80E-02 | 1.08E-02 |  |
| rs11873305 | 18 | 60381959 | A | 0.037 | -0.059 | 0.006 | 1.76E-20 | 0.015 | 0.032 | 6.34E-01 | 4.20E-01 |  |
| rs11943456 | 4 | 55410167 | C | 0.464 | 0.017 | 0.002 | 5.74E-12 | 0.017 | 0.012 | 1.54E-01 | 7.60E-02 |  |
| rs12037698 | 1 | 243421043 | G | 0.144 | -0.022 | 0.004 | 2.86E-10 | -0.017 | 0.018 | 3.41E-01 | 3.44E-01 |  |
| rs12072739 | 1 | 97850337 | G | 0.255 | 0.018 | 0.003 | 5.43E-10 | -0.008 | 0.014 | 5.82E-01 | 6.34E-01 |  |
| rs12124126 | 1 | 6600289 | A | 0.327 | -0.015 | 0.003 | 1.12E-08 | -0.012 | 0.013 | 3.42E-01 | 9.40E-01 |  |
| rs12127506 | 1 | 221902272 | C | 0.327 | -0.015 | 0.003 | 1.96E-08 | 0.054 | 0.013 | 3.14E-05 | 9.99E-01 |  |
| rs1218822 | 13 | 27437826 | A | 0.662 | 0.015 | 0.003 | 2.50E-09 | -0.019 | 0.013 | 1.45E-01 | 8.52E-01 |  |
| rs12218858 | 10 | 124785631 | T | 0.572 | 0.015 | 0.002 | 3.90E-09 | -0.003 | 0.012 | 8.15E-01 | 6.72E-02 |  |
| rs12330631 | 3 | 123370987 | C | 0.370 | -0.017 | 0.003 | 3.69E-11 | -0.011 | 0.015 | 4.56E-01 | 7.08E-01 |  |
| rs12339822 | 9 | 89572263 | G | 0.536 | 0.019 | 0.002 | 5.97E-14 | -0.015 | 0.012 | 2.21E-01 | 8.82E-01 |  |
| rs12367809 | 12 | 49862280 | T | 0.376 | 0.025 | 0.003 | 1.13E-22 | 0.005 | 0.013 | 6.65E-01 | 1.78E-01 |  |
| rs12370302 | 12 | 97546011 | C | 0.130 | 0.022 | 0.004 | 2.13E-09 | 0.017 | 0.019 | 3.67E-01 | 1.94E-01 |  |
| rs12477088 | 2 | 67614194 | T | 0.412 | -0.016 | 0.002 | 1.26E-10 | 0.001 | 0.012 | 9.15E-01 | 4.85E-01 |  |
| rs12477385 | 2 | 165288340 | G | 0.228 | -0.017 | 0.003 | 4.11E-09 | -0.041 | 0.015 | 5.65E-03 | 8.69E-01 |  |
| rs12550674 | 8 | 9875989 | A | 0.670 | -0.015 | 0.003 | 6.54E-09 | -0.007 | 0.013 | 6.21E-01 | 4.49E-02 |  |
| rs12568522 | 1 | 102111408 | G | 0.315 | -0.015 | 0.003 | 2.51E-08 | -0.016 | 0.013 | 2.37E-01 | 1.05E-02 |  |
| rs12616638 | 2 | 54071535 | A | 0.440 | -0.014 | 0.002 | 3.74E-08 | 0.003 | 0.013 | 7.98E-01 | 4.61E-01 |  |
| rs12619178 | 2 | 100221695 | C | 0.396 | -0.016 | 0.003 | 1.37E-10 | 0.006 | 0.012 | 6.08E-01 | 1.80E-01 |  |
| rs12622267 | 2 | 180703280 | A | 0.355 | -0.017 | 0.003 | 9.35E-11 | 0.005 | 0.013 | 7.04E-01 | 8.15E-01 |  |
| rs12679106 | 8 | 72530963 | G | 0.715 | -0.023 | 0.003 | 9.33E-17 | 0.006 | 0.013 | 6.77E-01 | 4.57E-02 |  |
| rs12702528 | 7 | 6561482 | C | 0.444 | -0.014 | 0.002 | 5.42E-09 | 0.013 | 0.013 | 3.38E-01 | 1.94E-01 |  |
| rs12890931 | 14 | 69286652 | G | 0.378 | 0.016 | 0.003 | 6.14E-10 | -0.010 | 0.013 | 4.33E-01 | 3.61E-01 |  |
| rs12901071 | 15 | 67078051 | G | 0.338 | 0.016 | 0.003 | 8.66E-10 | -0.033 | 0.013 | 1.27E-02 | 1.67E-01 |  |
| rs12923476 | 16 | 24786758 | G | 0.249 | -0.020 | 0.003 | 5.66E-13 | 0.007 | 0.014 | 6.34E-01 | 5.06E-01 |  |
| rs12962597 | 18 | 42116636 | T | 0.099 | 0.024 | 0.004 | 2.18E-08 | -0.016 | 0.021 | 4.55E-01 | 3.99E-03 |  |
| rs1296328 | 4 | 136162038 | A | 0.548 | -0.016 | 0.002 | 5.40E-11 | -0.013 | 0.013 | 2.81E-01 | 2.66E-01 |  |
| rs13062093 | 3 | 35625565 | G | 0.364 | 0.017 | 0.003 | 2.74E-11 | 0.015 | 0.013 | 2.30E-01 | 9.02E-01 |  |
| rs13069856 | 3 | 50163356 | T | 0.067 | -0.028 | 0.005 | 2.18E-08 | 0.009 | 0.027 | 7.48E-01 | 3.34E-01 |  |
| rs13109830 | 4 | 82287804 | A | 0.468 | -0.015 | 0.002 | 2.60E-09 | 0.022 | 0.012 | 6.76E-02 | 8.82E-01 |  |
| rs13135092 | 4 | 102276925 | G | 0.077 | 0.048 | 0.004 | 1.10E-26 | 0.043 | 0.022 | 5.55E-02 | 4.96E-01 |  |
| rs13174863 | 5 | 139701160 | G | 0.147 | 0.021 | 0.003 | 2.80E-09 | 0.006 | 0.018 | 7.61E-01 | 7.44E-01 |  |
| rs1322842 | 6 | 20488666 | A | 0.610 | -0.014 | 0.003 | 1.69E-08 | 0.006 | 0.013 | 6.07E-01 | 9.44E-02 |  |
| rs1322998 | 10 | 110773038 | A | 0.426 | 0.014 | 0.002 | 2.07E-08 | 0.008 | 0.012 | 5.08E-01 | 4.32E-03 |  |
| rs1324088 | 6 | 25840894 | A | 0.126 | 0.019 | 0.004 | 3.34E-08 | 0.006 | 0.018 | 7.62E-01 | 7.17E-01 |  |
| rs1335055 | 1 | 107081941 | G | 0.655 | -0.015 | 0.003 | 1.29E-08 | -0.021 | 0.013 | 1.02E-01 | 9.34E-01 |  |
| rs136309 | 22 | 30779538 | A | 0.196 | 0.017 | 0.003 | 1.27E-08 | -0.009 | 0.015 | 5.55E-01 | 3.85E-01 |  |
| rs141403828 | 15 | 86129053 | A | 0.013 | -0.061 | 0.011 | 3.23E-08 | -0.058 | 0.057 | 3.10E-01 | 7.82E-02 |  |
| rs1414506 | 6 | 51622618 | T | 0.646 | 0.015 | 0.003 | 2.63E-09 | 0.003 | 0.013 | 7.86E-01 | 3.14E-01 |  |
| rs1415475 | 9 | 16591504 | T | 0.061 | -0.033 | 0.005 | 4.00E-11 | -0.006 | 0.025 | 8.12E-01 | 8.01E-01 |  |
| rs1441264 | 13 | 79006784 | A | 0.602 | 0.016 | 0.003 | 4.91E-11 | 0.023 | 0.012 | 6.37E-02 | 8.57E-01 |  |
| rs1446585 | 2 | 135649909 | A | 0.278 | -0.019 | 0.003 | 4.38E-11 | 0.042 | 0.016 | 1.05E-02 | 4.85E-01 |  |
| rs145350287 | 12 | 120469506 | T | 0.038 | -0.046 | 0.006 | 2.56E-13 | 0.003 | 0.033 | 9.21E-01 | 9.91E-01 |  |
| rs1460940 | 1 | 72348934 | A | 0.812 | 0.027 | 0.003 | 2.81E-18 | 0.025 | 0.016 | 1.12E-01 | 7.99E-01 |  |
| rs147730268 | 12 | 122539929 | G | 0.090 | -0.040 | 0.004 | 1.93E-20 | 0.013 | 0.022 | 5.75E-01 | 7.10E-01 |  |
| rs1486921 | 8 | 77963891 | C | 0.299 | -0.015 | 0.003 | 3.17E-08 | -0.004 | 0.013 | 7.38E-01 | 7.59E-01 |  |
| rs1491592 | 3 | 70605093 | T | 0.558 | 0.014 | 0.002 | 7.40E-09 | -0.022 | 0.012 | 7.20E-02 | 1.14E-01 |  |
| rs1503526 | 5 | 63724879 | C | 0.499 | 0.016 | 0.002 | 6.20E-11 | 0.000 | 0.012 | 1.00E+00 | 8.15E-01 |  |
| rs1535274 | 6 | 26520519 | C | 0.594 | 0.017 | 0.003 | 1.00E-11 | 0.021 | 0.013 | 9.90E-02 | 5.61E-01 |  |
| rs1538742 | 1 | 219494910 | C | 0.580 | -0.025 | 0.002 | 9.23E-25 | -0.020 | 0.012 | 1.07E-01 | 4.96E-01 |  |
| rs1568489 | 3 | 153955892 | G | 0.584 | 0.015 | 0.002 | 1.02E-09 | -0.001 | 0.012 | 9.30E-01 | 7.11E-01 |  |
| rs157845 | 5 | 56500812 | T | 0.737 | -0.017 | 0.003 | 3.45E-09 | 0.036 | 0.014 | 1.02E-02 | 4.88E-01 |  |
| rs1609906 | 3 | 94314755 | G | 0.528 | -0.020 | 0.002 | 9.13E-17 | 0.014 | 0.015 | 3.44E-01 | 9.18E-01 |  |
| rs1653892 | 7 | 32359857 | T | 0.697 | -0.017 | 0.003 | 1.66E-10 | 0.015 | 0.013 | 2.65E-01 | 8.63E-02 |  |
| rs16934748 | 10 | 33681391 | C | 0.152 | 0.019 | 0.003 | 3.11E-08 | 0.027 | 0.017 | 1.10E-01 | 5.65E-03 |  |
| rs16940859 | 18 | 24604457 | A | 0.190 | -0.018 | 0.003 | 1.13E-08 | -0.014 | 0.016 | 3.85E-01 | 2.79E-01 |  |
| rs16951304 | 15 | 67797280 | T | 0.190 | -0.027 | 0.003 | 9.49E-19 | -0.056 | 0.015 | 2.81E-04 | 8.21E-01 |  |
| rs1701820 | 7 | 113313425 | G | 0.553 | -0.015 | 0.002 | 2.49E-09 | -0.013 | 0.012 | 2.90E-01 | 2.65E-01 |  |
| rs17024393 | 1 | 109612066 | C | 0.031 | 0.060 | 0.008 | 1.17E-14 | 0.021 | 0.037 | 5.71E-01 | 8.66E-01 |  |
| rs17115183 | 14 | 29668201 | T | 0.402 | 0.015 | 0.003 | 2.59E-09 | 0.020 | 0.012 | 1.11E-01 | 5.97E-01 |  |
| rs17265513 | 20 | 41203988 | C | 0.206 | 0.018 | 0.003 | 1.85E-09 | 0.012 | 0.015 | 4.18E-01 | 9.55E-01 |  |
| rs17296856 | 15 | 83888698 | A | 0.289 | -0.027 | 0.003 | 5.13E-23 | 0.007 | 0.013 | 5.99E-01 | 4.56E-01 |  |
| rs1731260 | 2 | 26730486 | T | 0.476 | 0.015 | 0.002 | 8.19E-10 | 0.009 | 0.012 | 4.42E-01 | 3.55E-01 |  |
| rs17704028 | 7 | 95510719 | C | 0.156 | -0.019 | 0.003 | 4.29E-08 | -0.011 | 0.017 | 5.06E-01 | 1.37E-01 |  |
| rs17770336 | 9 | 28414627 | T | 0.330 | 0.021 | 0.003 | 2.81E-15 | 0.017 | 0.013 | 1.97E-01 | 5.30E-01 |  |
| rs1783541 | 11 | 65527328 | T | 0.196 | 0.017 | 0.003 | 2.29E-08 | 0.012 | 0.015 | 4.34E-01 | 3.45E-01 |  |
| rs1881505 | 11 | 1461947 | T | 0.946 | -0.029 | 0.005 | 2.22E-08 | -0.006 | 0.030 | 8.46E-01 | 4.84E-01 |  |
| rs1891215 | 1 | 7667794 | C | 0.466 | 0.014 | 0.002 | 1.61E-08 | 0.006 | 0.012 | 6.21E-01 | 1.02E-02 |  |
| rs1893659 |  |  | C | 0.458 | -0.020 | 0.002 | 4.42E-16 | -0.002 | 0.012 | 8.52E-01 | 1.76E-01 |  |
| rs1928185 | 6 | 50967800 | C | 0.178 | 0.031 | 0.003 | 1.35E-20 | 0.000 | 0.016 | 9.79E-01 | 4.97E-01 |  |
| rs1928496 | 13 | 30438767 | T | 0.732 | 0.018 | 0.003 | 3.11E-10 | -0.014 | 0.014 | 3.24E-01 | 2.53E-01 |  |
| rs2002023 | 10 | 75088766 | T | 0.419 | 0.016 | 0.003 | 5.42E-10 | 0.018 | 0.012 | 1.38E-01 | 8.91E-01 |  |
| rs2038646 | 1 | 96474815 | G | 0.595 | 0.019 | 0.003 | 1.24E-13 | 0.016 | 0.012 | 1.96E-01 | 3.82E-01 |  |
| rs2042864 | 3 | 141460137 | C | 0.382 | 0.016 | 0.003 | 6.65E-11 | 0.014 | 0.013 | 2.81E-01 | 4.20E-02 |  |
| rs2043016 | 2 | 197281657 | T | 0.391 | 0.016 | 0.003 | 2.11E-10 | 0.016 | 0.013 | 1.93E-01 | 7.04E-01 |  |
| rs2052607 | 18 | 43208422 | G | 0.357 | -0.020 | 0.003 | 3.95E-14 | -0.017 | 0.013 | 1.94E-01 | 8.63E-02 |  |
| rs208015 | 17 | 48174984 | T | 0.927 | -0.034 | 0.005 | 6.39E-12 | 0.010 | 0.024 | 6.86E-01 | 1.20E-01 |  |
| rs2112347 | 5 | 75719417 | T | 0.370 | -0.023 | 0.003 | 6.07E-19 | -0.006 | 0.013 | 6.27E-01 | 5.18E-01 |  |
| rs2172131 | 10 | 132165458 | T | 0.562 | -0.017 | 0.002 | 1.52E-11 | 0.020 | 0.013 | 1.37E-01 | 3.10E-01 |  |
| rs217672 | 14 | 61894303 | C | 0.264 | 0.016 | 0.003 | 4.30E-09 | 0.004 | 0.014 | 7.86E-01 | 2.81E-01 |  |
| rs2192527 | 4 | 18328201 | G | 0.451 | 0.018 | 0.002 | 1.66E-13 | -0.014 | 0.012 | 2.49E-01 | 9.68E-03 |  |
| rs2239647 | 14 | 32823537 | A | 0.552 | -0.017 | 0.002 | 2.05E-11 | -0.012 | 0.014 | 3.82E-01 | 9.52E-01 |  |
| rs2242449 | 17 | 7192188 | T | 0.427 | 0.014 | 0.002 | 7.22E-09 | -0.002 | 0.012 | 8.90E-01 | 6.08E-01 |  |
| rs2243928 | 13 | 111329360 | C | 0.635 | -0.018 | 0.003 | 6.02E-12 | 0.003 | 0.013 | 7.99E-01 | 2.30E-01 |  |
| rs2281819 | 6 | 33803896 | T | 0.238 | -0.018 | 0.003 | 1.15E-09 | -0.004 | 0.014 | 8.01E-01 | 9.15E-01 |  |
| rs2289379 | 7 | 44764626 | C | 0.422 | -0.017 | 0.003 | 4.82E-11 | -0.031 | 0.015 | 4.20E-02 | 5.98E-01 |  |
| rs2306937 | 1 | 112703884 | C | 0.220 | -0.019 | 0.003 | 3.94E-10 | -0.030 | 0.015 | 4.57E-02 | 9.73E-02 |  |
| rs2318543 | 4 | 66937545 | A | 0.777 | -0.018 | 0.003 | 1.62E-09 | -0.001 | 0.015 | 9.34E-01 | 5.84E-01 |  |
| rs2370982 | 14 | 79424334 | T | 0.220 | 0.026 | 0.003 | 2.95E-18 | 0.003 | 0.015 | 8.19E-01 | 7.32E-02 |  |
| rs2371767 | 3 | 64732582 | C | 0.268 | 0.018 | 0.003 | 1.14E-10 | -0.001 | 0.014 | 9.56E-01 | 4.33E-01 |  |
| rs2398861 | 9 | 93668465 | G | 0.268 | 0.016 | 0.003 | 7.31E-09 | -0.002 | 0.014 | 8.90E-01 | 1.11E-01 |  |
| rs241459 | 1 | 49291596 | A | 0.670 | -0.020 | 0.003 | 3.86E-14 | -0.005 | 0.013 | 7.08E-01 | 8.99E-01 |  |
| rs2436772 | 2 | 47056418 | G | 0.204 | -0.020 | 0.003 | 1.11E-10 | -0.011 | 0.016 | 4.85E-01 | 7.64E-01 |  |
| rs2439823 | 10 | 98018469 | G | 0.537 | 0.019 | 0.002 | 1.82E-14 | -0.004 | 0.013 | 7.68E-01 | 1.25E-01 |  |
| rs2455821 | 3 | 15640433 | A | 0.274 | 0.016 | 0.003 | 5.50E-09 | -0.001 | 0.014 | 9.49E-01 | 5.34E-01 |  |
| rs245775 | 5 | 171105101 | G | 0.717 | 0.019 | 0.003 | 1.97E-12 | -0.007 | 0.014 | 6.33E-01 | 6.21E-01 |  |
| rs2477534 | 10 | 94403282 | C | 0.638 | -0.014 | 0.003 | 3.22E-08 | -0.014 | 0.013 | 2.88E-01 | 5.66E-01 |  |
| rs249612 | 5 | 66904955 | T | 0.716 | 0.015 | 0.003 | 2.59E-08 | -0.001 | 0.014 | 9.14E-01 | 2.37E-01 |  |
| rs254024 | 5 | 104608319 | T | 0.428 | 0.014 | 0.002 | 5.80E-09 | -0.016 | 0.012 | 1.84E-01 | 3.42E-01 |  |
| rs2606228 | 3 | 183819971 | A | 0.637 | -0.016 | 0.003 | 4.87E-10 | -0.021 | 0.013 | 9.73E-02 | 7.38E-01 |  |
| rs2660241 | 16 | 4890022 | C | 0.363 | 0.015 | 0.003 | 2.24E-09 | -0.023 | 0.013 | 6.69E-02 | 4.93E-01 |  |
| rs2665856 | 17 | 63905361 | T | 0.677 | -0.015 | 0.003 | 2.79E-08 | -0.038 | 0.013 | 3.92E-03 | 3.14E-05 |  |
| rs2678204 | 1 | 201831383 | G | 0.340 | 0.021 | 0.003 | 3.68E-16 | 0.041 | 0.013 | 1.38E-03 | 6.22E-01 |  |
| rs2798297 | 4 | 3062277 | A | 0.362 | 0.015 | 0.003 | 3.44E-09 | 0.011 | 0.013 | 4.10E-01 | 2.37E-01 |  |
| rs2802774 | 1 | 203558684 | A | 0.545 | 0.014 | 0.002 | 4.90E-09 | 0.006 | 0.014 | 6.62E-01 | 2.90E-01 |  |
| rs28434748 | 18 | 65638772 | G | 0.791 | -0.017 | 0.003 | 4.31E-08 | 0.017 | 0.015 | 2.79E-01 | 1.02E-01 |  |
| rs28483178 | 19 | 33486300 | G | 0.284 | 0.024 | 0.003 | 2.36E-19 | -0.006 | 0.014 | 6.58E-01 | 6.76E-02 |  |
| rs2855818 | 17 | 44212647 | A | 0.246 | 0.024 | 0.003 | 2.21E-16 | -0.016 | 0.014 | 2.61E-01 | 3.39E-02 |  |
| rs28726372 | 1 | 83888156 | C | 0.306 | 0.016 | 0.003 | 3.04E-09 | -0.007 | 0.014 | 5.88E-01 | 5.71E-01 |  |
| rs28893270 | 2 | 69404777 | G | 0.406 | -0.015 | 0.002 | 1.13E-09 | 0.008 | 0.013 | 5.34E-01 | 3.92E-03 |  |
| rs2914231 | 5 | 158584427 | G | 0.222 | -0.022 | 0.003 | 8.30E-14 | -0.025 | 0.015 | 8.53E-02 | 7.49E-02 |  |
| rs2943650 | 2 | 226241205 | C | 0.643 | -0.021 | 0.003 | 1.22E-15 | 0.023 | 0.013 | 7.60E-02 | 9.34E-01 |  |
| rs2954033 | 8 | 125481504 | G | 0.703 | 0.016 | 0.003 | 4.84E-09 | -0.019 | 0.015 | 2.24E-01 | 1.22E-02 |  |
| rs2980240 | 8 | 75399875 | C | 0.816 | 0.023 | 0.003 | 3.20E-13 | -0.004 | 0.016 | 7.85E-01 | 3.38E-01 |  |
| rs2984618 | 1 | 47224766 | T | 0.406 | 0.015 | 0.002 | 2.70E-09 | -0.001 | 0.012 | 9.61E-01 | 4.02E-01 |  |
| rs301806 | 1 | 8422018 | T | 0.591 | 0.016 | 0.002 | 2.20E-10 | 0.022 | 0.012 | 7.13E-02 | 2.88E-01 |  |
| rs314279 | 6 | 104954208 | C | 0.884 | -0.022 | 0.004 | 8.46E-09 | -0.001 | 0.019 | 9.44E-01 | 9.06E-01 |  |
| rs33503 | 3 | 42386465 | G | 0.817 | -0.017 | 0.003 | 2.04E-08 | -0.006 | 0.016 | 7.11E-01 | 6.07E-01 |  |
| rs34220678 | 12 | 122417675 | A | 0.107 | 0.023 | 0.004 | 2.39E-09 | 0.072 | 0.063 | 2.55E-01 | 3.53E-02 |  |
| rs34356467 | 17 | 4075237 | T | 0.180 | 0.017 | 0.003 | 3.96E-08 | 0.004 | 0.016 | 8.12E-01 | 4.33E-01 |  |
| rs34417222 | 20 | 48833526 | C | 0.306 | -0.017 | 0.003 | 2.23E-10 | -0.012 | 0.013 | 3.55E-01 | 5.79E-01 |  |
| rs34483452 | 5 | 88690497 | A | 0.137 | 0.031 | 0.004 | 4.54E-18 | 0.034 | 0.018 | 5.97E-02 | 5.34E-01 |  |
| rs34580448 | 5 | 83515065 | T | 0.039 | -0.045 | 0.006 | 4.32E-13 | -0.022 | 0.032 | 4.82E-01 | 7.98E-01 |  |
| rs34769775 | 15 | 80696831 | C | 0.291 | -0.018 | 0.003 | 3.12E-11 | -0.024 | 0.014 | 7.82E-02 | 8.51E-01 |  |
| rs34811474 | 4 | 25407216 | G | 0.227 | -0.017 | 0.003 | 3.05E-09 | 0.036 | 0.021 | 8.63E-02 | 9.49E-01 |  |
| rs34898535 | 16 | 31014320 | C | 0.379 | -0.021 | 0.003 | 3.34E-17 | -0.019 | 0.013 | 1.32E-01 | 2.15E-01 |  |
| rs34966008 | 17 | 36563784 | C | 0.402 | -0.017 | 0.003 | 1.39E-11 | 0.001 | 0.012 | 9.55E-01 | 2.53E-01 |  |
| rs35060985 | 11 | 43671560 | A | 0.313 | 0.023 | 0.003 | 7.84E-18 | 0.009 | 0.013 | 5.02E-01 | 2.70E-01 |  |
| rs35128308 | 10 | 86350989 | C | 0.501 | 0.015 | 0.002 | 1.47E-09 | 0.023 | 0.012 | 5.81E-02 | 3.48E-01 |  |
| rs35154152 | 1 | 155202934 | T | 0.095 | -0.023 | 0.004 | 1.05E-08 | 0.005 | 0.021 | 8.19E-01 | 1.23E-01 |  |
| rs35557355 | 2 | 57019659 | T | 0.396 | 0.014 | 0.003 | 2.43E-08 | 0.016 | 0.013 | 2.08E-01 | 6.73E-01 |  |
| rs35644221 | 12 | 89834002 | G | 0.318 | -0.017 | 0.003 | 4.53E-10 | 0.013 | 0.013 | 3.45E-01 | 8.88E-01 |  |
| rs35761930 | 18 | 13182326 | T | 0.190 | 0.019 | 0.003 | 4.62E-09 | 0.000 | 0.016 | 9.92E-01 | 5.08E-01 |  |
| rs35882248 | 2 | 229763239 | T | 0.323 | 0.022 | 0.003 | 3.12E-16 | 0.008 | 0.013 | 5.57E-01 | 2.25E-01 |  |
| rs36090025 | 10 | 113014674 | A | 0.283 | -0.015 | 0.003 | 4.62E-08 | 0.008 | 0.013 | 5.71E-01 | 4.75E-01 |  |
| rs3736896 | 6 | 34768106 | C | 0.212 | 0.032 | 0.003 | 1.78E-25 | -0.043 | 0.015 | 4.35E-03 | 3.09E-01 |  |
| rs3764002 | 12 | 108224853 | C | 0.279 | -0.023 | 0.003 | 1.36E-16 | 0.011 | 0.016 | 5.02E-01 | 6.03E-01 |  |
| rs3766823 | 1 | 31731656 | A | 0.185 | 0.021 | 0.003 | 8.17E-11 | 0.024 | 0.020 | 2.35E-01 | 4.12E-01 |  |
| rs3796658 | 4 | 88787090 | G | 0.478 | -0.018 | 0.002 | 7.05E-13 | 0.000 | 0.013 | 9.91E-01 | 6.21E-01 |  |
| rs3803286 | 14 | 102780133 | A | 0.667 | -0.018 | 0.003 | 8.42E-12 | 0.013 | 0.013 | 3.14E-01 | 2.08E-01 |  |
| rs3810291 | 19 | 47065746 | A | 0.669 | 0.018 | 0.003 | 1.87E-11 | -0.009 | 0.014 | 4.98E-01 | 9.20E-03 |  |
| rs3817428 | 15 | 88872016 | C | 0.277 | -0.022 | 0.003 | 4.28E-15 | -0.012 | 0.015 | 4.38E-01 | 4.34E-02 |  |
| rs3826226 | 16 | 1425686 | A | 0.369 | 0.014 | 0.003 | 4.77E-08 | -0.002 | 0.014 | 8.88E-01 | 7.56E-02 |  |
| rs3847072 | 7 | 105451072 | G | 0.286 | -0.016 | 0.003 | 4.33E-08 | -0.060 | 0.033 | 7.32E-02 | 7.20E-02 |  |
| rs40071 | 5 | 108160401 | T | 0.183 | -0.021 | 0.003 | 3.74E-11 | -0.014 | 0.016 | 3.66E-01 | 6.39E-01 |  |
| rs41310284 | 10 | 100687890 | C | 0.096 | -0.026 | 0.004 | 3.20E-10 | 0.011 | 0.022 | 6.25E-01 | 9.20E-02 |  |
| rs4148866 | 12 | 122941028 | T | 0.408 | 0.014 | 0.002 | 2.48E-08 | -0.008 | 0.015 | 6.03E-01 | 8.90E-01 |  |
| rs4238585 | 16 | 20243775 | T | 0.856 | 0.021 | 0.004 | 8.05E-09 | -0.019 | 0.018 | 3.05E-01 | 1.26E-01 |  |
| rs4284389 | 11 | 10053668 | T | 0.502 | 0.016 | 0.002 | 1.83E-10 | 0.041 | 0.012 | 7.23E-04 | 1.84E-01 |  |
| rs4402589 | 16 | 29943333 | G | 0.543 | 0.020 | 0.002 | 2.38E-15 | 0.018 | 0.013 | 1.69E-01 | 6.62E-01 |  |
| rs4450871 | 4 | 4988571 | G | 0.447 | 0.014 | 0.002 | 5.73E-09 | -0.027 | 0.018 | 1.26E-01 | 4.35E-01 |  |
| rs4477562 | 13 | 53530833 | T | 0.130 | 0.022 | 0.004 | 2.69E-09 | 0.019 | 0.018 | 2.97E-01 | 1.96E-01 |  |
| rs4482463 | 2 | 204511186 | C | 0.925 | -0.026 | 0.005 | 1.77E-08 | -0.050 | 0.023 | 3.11E-02 | 8.15E-01 |  |
| rs4502882 | 5 | 153714438 | C | 0.660 | -0.015 | 0.003 | 3.30E-09 | -0.004 | 0.013 | 7.64E-01 | 5.17E-01 |  |
| rs4503172 | 9 | 121878562 | C | 0.608 | -0.015 | 0.003 | 5.63E-09 | -0.026 | 0.012 | 3.39E-02 | 7.49E-01 |  |
| rs4547132 | 11 | 112962091 | C | 0.307 | -0.018 | 0.003 | 2.09E-10 | 0.005 | 0.014 | 7.10E-01 | 8.19E-02 |  |
| rs4547574 | 2 | 48313864 | A | 0.217 | -0.016 | 0.003 | 2.40E-08 | -0.010 | 0.015 | 5.18E-01 | 9.61E-01 |  |
| rs4549080 | 2 | 228143731 | T | 0.338 | 0.017 | 0.003 | 2.32E-11 | 0.014 | 0.013 | 2.88E-01 | 5.81E-02 |  |
| rs4549685 | 7 | 39286879 | C | 0.329 | -0.018 | 0.003 | 1.57E-11 | 0.020 | 0.013 | 1.14E-01 | 1.11E-01 |  |
| rs4619804 | 3 | 18633152 | C | 0.727 | 0.018 | 0.003 | 5.80E-11 | 0.027 | 0.014 | 5.30E-02 | 4.91E-03 |  |
| rs4642249 | 4 | 20087388 | A | 0.889 | -0.024 | 0.004 | 1.19E-09 | -0.038 | 0.020 | 5.32E-02 | 2.15E-01 |  |
| rs4657796 |  |  | T | 0.371 | -0.016 | 0.003 | 2.06E-10 | -0.001 | 0.013 | 9.52E-01 | 4.10E-01 |  |
| rs4671328 | 2 | 58708147 | T | 0.561 | -0.018 | 0.002 | 7.88E-14 | 0.012 | 0.012 | 3.34E-01 | 4.42E-01 |  |
| rs4690324 | 4 | 966293 | G | 0.827 | -0.019 | 0.003 | 2.64E-09 | 0.004 | 0.017 | 8.15E-01 | 9.30E-01 |  |
| rs4703838 | 5 | 81688461 | T | 0.192 | -0.017 | 0.003 | 4.10E-08 | -0.011 | 0.016 | 4.97E-01 | 1.07E-01 |  |
| rs4709745 | 6 | 163684952 | C | 0.300 | 0.015 | 0.003 | 3.53E-08 | 0.009 | 0.013 | 5.17E-01 | 9.14E-01 |  |
| rs4718964 | 7 | 70573983 | T | 0.414 | 0.017 | 0.002 | 4.07E-12 | 0.003 | 0.012 | 8.21E-01 | 6.69E-02 |  |
| rs4722398 | 7 | 3085586 | T | 0.124 | 0.020 | 0.004 | 3.03E-08 | 0.028 | 0.018 | 1.34E-01 | 7.86E-01 |  |
| rs4727630 | 7 | 78204884 | G | 0.569 | 0.014 | 0.002 | 2.21E-08 | 0.010 | 0.012 | 4.12E-01 | 3.53E-01 |  |
| rs4752183 | 10 | 118638857 | T | 0.559 | -0.014 | 0.002 | 3.11E-08 | 0.008 | 0.013 | 5.34E-01 | 1.39E-01 |  |
| rs4759318 | 12 | 54026314 | T | 0.364 | 0.016 | 0.003 | 3.52E-10 | 0.026 | 0.013 | 3.74E-02 | 1.45E-01 |  |
| rs477895 | 11 | 64281440 | T | 0.841 | 0.018 | 0.003 | 3.13E-08 | -0.002 | 0.017 | 9.21E-01 | 9.71E-01 |  |
| rs4785955 | 16 | 4247650 | T | 0.222 | 0.017 | 0.003 | 2.14E-08 | -0.016 | 0.016 | 3.06E-01 | 7.32E-01 |  |
| rs479018 | 11 | 66293075 | G | 0.326 | -0.019 | 0.003 | 1.15E-13 | -0.027 | 0.013 | 4.49E-02 | 1.38E-01 |  |
| rs4790841 | 17 | 1932188 | C | 0.160 | -0.031 | 0.003 | 7.52E-20 | 0.012 | 0.018 | 5.00E-01 | 3.78E-02 |  |
| rs4821764 | 22 | 38203357 | G | 0.582 | -0.021 | 0.002 | 9.42E-18 | 0.026 | 0.012 | 3.58E-02 | 2.24E-01 |  |
| rs4864201 | 4 | 129810129 | T | 0.645 | -0.015 | 0.003 | 8.56E-09 | 0.009 | 0.013 | 4.93E-01 | 7.23E-04 |  |
| rs4876611 | 8 | 115659621 | G | 0.720 | 0.023 | 0.003 | 4.34E-17 | 0.020 | 0.014 | 1.43E-01 | 7.17E-01 |  |
| rs4976033 | 5 | 68418419 | A | 0.405 | -0.017 | 0.003 | 2.72E-11 | 0.009 | 0.013 | 4.84E-01 | 4.66E-03 |  |
| rs538656 | 18 | 60183189 | T | 0.226 | 0.030 | 0.003 | 1.13E-25 | 0.011 | 0.014 | 4.66E-01 | 7.74E-03 |  |
| rs539515 | 1 | 177919890 | C | 0.196 | 0.036 | 0.003 | 7.41E-33 | -0.016 | 0.015 | 2.84E-01 | 5.88E-01 |  |
| rs55637757 | 16 | 89469480 | C | 0.135 | -0.022 | 0.004 | 4.84E-10 | -0.006 | 0.019 | 7.55E-01 | 4.33E-01 |  |
| rs55714539 | 19 | 18096587 | C | 0.353 | 0.025 | 0.003 | 4.20E-22 | 0.005 | 0.013 | 7.07E-01 | 9.35E-01 |  |
| rs56226325 | 7 | 2039346 | C | 0.167 | -0.022 | 0.003 | 7.50E-11 | -0.036 | 0.017 | 3.35E-02 | 7.13E-02 |  |
| rs56288810 | 17 | 54854376 | G | 0.219 | 0.018 | 0.003 | 2.49E-09 | 0.021 | 0.015 | 1.61E-01 | 9.71E-01 |  |
| rs56399737 | 13 | 32807583 | C | 0.446 | -0.015 | 0.002 | 3.46E-09 | -0.001 | 0.012 | 9.34E-01 | 1.27E-02 |  |
| rs56803094 | 15 | 98679280 | A | 0.234 | -0.018 | 0.003 | 6.32E-10 | 0.005 | 0.015 | 7.44E-01 | 3.74E-02 |  |
| rs57292329 | 4 | 72684550 | A | 0.069 | 0.029 | 0.005 | 1.40E-08 | -0.023 | 0.025 | 3.50E-01 | 1.00E+00 |  |
| rs57866767 | 10 | 94263320 | T | 0.427 | -0.021 | 0.002 | 8.24E-18 | 0.032 | 0.012 | 1.04E-02 | 1.93E-01 |  |
| rs58360798 | 2 | 112344343 | C | 0.185 | 0.018 | 0.003 | 1.34E-08 | 0.002 | 0.016 | 8.84E-01 | 9.49E-01 |  |
| rs586461 | 13 | 85767924 | C | 0.241 | -0.017 | 0.003 | 1.91E-09 | -0.003 | 0.014 | 8.21E-01 | 7.86E-01 |  |
| rs591939 | 17 | 42546057 | G | 0.242 | 0.017 | 0.003 | 1.03E-09 | 0.050 | 0.014 | 5.18E-04 | 8.90E-01 |  |
| rs597309 | 18 | 60489058 | A | 0.170 | -0.018 | 0.003 | 3.11E-08 | -0.006 | 0.016 | 7.17E-01 | 2.81E-01 |  |
| rs6021948 | 20 | 52483881 | T | 0.305 | -0.017 | 0.003 | 2.78E-10 | 0.002 | 0.013 | 8.91E-01 | 6.37E-02 |  |
| rs61778068 | 1 | 74518677 | A | 0.038 | -0.035 | 0.006 | 1.24E-08 | 0.029 | 0.033 | 3.78E-01 | 8.98E-01 |  |
| rs61888762 | 11 | 27688083 | G | 0.312 | 0.027 | 0.003 | 1.92E-24 | -0.014 | 0.013 | 2.96E-01 | 4.23E-01 |  |
| rs61975142 | 14 | 58935872 | G | 0.180 | -0.022 | 0.003 | 6.34E-11 | -0.003 | 0.016 | 8.51E-01 | 8.21E-01 |  |
| rs62037365 | 16 | 28857641 | G | 0.399 | 0.031 | 0.003 | 3.37E-35 | -0.004 | 0.013 | 7.76E-01 | 4.34E-01 |  |
| rs62104477 | 19 | 29804084 | T | 0.338 | 0.019 | 0.003 | 1.66E-13 | 0.020 | 0.013 | 1.23E-01 | 3.15E-01 |  |
| rs62106258 | 2 | 417167 | T | 0.041 | -0.065 | 0.006 | 3.10E-30 | -0.024 | 0.046 | 5.97E-01 | 4.02E-01 |  |
| rs62261725 | 3 | 85849476 | A | 0.330 | -0.020 | 0.003 | 2.49E-14 | 0.024 | 0.013 | 6.72E-02 | 7.47E-01 |  |
| rs62277722 | 3 | 90224980 | A | 0.072 | 0.030 | 0.005 | 4.67E-09 | 0.028 | 0.075 | 7.13E-01 | 9.65E-02 |  |
| rs62285233 | 3 | 195181879 | G | 0.106 | -0.023 | 0.004 | 7.35E-09 | -0.034 | 0.021 | 1.17E-01 | 3.06E-01 |  |
| rs62414900 | 6 | 111937960 | C | 0.048 | -0.034 | 0.006 | 1.36E-09 | -0.003 | 0.029 | 9.07E-01 | 4.26E-01 |  |
| rs62477684 | 7 | 75472161 | C | 0.432 | -0.018 | 0.002 | 1.81E-13 | -0.002 | 0.013 | 8.82E-01 | 4.22E-01 |  |
| rs6545714 | 2 | 59080590 | G | 0.601 | -0.020 | 0.003 | 1.15E-15 | 0.000 | 0.012 | 9.99E-01 | 1.54E-01 |  |
| rs6561937 | 13 | 57683533 | T | 0.761 | -0.017 | 0.003 | 9.19E-10 | -0.010 | 0.014 | 4.88E-01 | 2.30E-01 |  |
| rs6575340 | 14 | 93557626 | A | 0.637 | 0.018 | 0.003 | 1.13E-12 | -0.033 | 0.013 | 1.04E-02 | 8.31E-01 |  |
| rs6686901 | 1 | 41970383 | C | 0.531 | -0.017 | 0.002 | 1.06E-11 | 0.008 | 0.012 | 5.30E-01 | 9.90E-02 |  |
| rs6688826 | 1 | 80346644 | C | 0.279 | 0.015 | 0.003 | 7.73E-09 | -0.020 | 0.013 | 1.39E-01 | 1.45E-01 |  |
| rs6717858 | 2 | 164683151 | C | 0.399 | 0.026 | 0.003 | 1.31E-24 | -0.010 | 0.012 | 4.12E-01 | 8.21E-01 |  |
| rs6739303 | 2 | 628749 | T | 0.828 | 0.034 | 0.003 | 2.27E-25 | 0.024 | 0.016 | 1.43E-01 | 5.55E-01 |  |
| rs67807996 | 1 | 150023307 | A | 0.377 | 0.022 | 0.003 | 3.34E-19 | -0.007 | 0.015 | 6.48E-01 | 2.33E-01 |  |
| rs6792984 | 3 | 108401337 | T | 0.307 | 0.015 | 0.003 | 2.75E-08 | -0.004 | 0.013 | 7.49E-01 | 5.18E-04 |  |
| rs68169458 | 8 | 66914972 | C | 0.294 | 0.017 | 0.003 | 8.71E-10 | -0.011 | 0.013 | 4.23E-01 | 2.88E-01 |  |
| rs6847975 | 4 | 79891806 | A | 0.339 | 0.017 | 0.003 | 2.25E-11 | 0.019 | 0.013 | 1.45E-01 | 6.93E-01 |  |
| rs6857 | 19 | 44888997 | C | 0.174 | -0.023 | 0.003 | 1.68E-12 | 0.050 | 0.017 | 3.33E-03 | 8.12E-01 |  |
| rs6861649 | 5 | 51568954 | C | 0.608 | 0.017 | 0.003 | 4.66E-12 | -0.005 | 0.012 | 6.93E-01 | 4.98E-01 |  |
| rs6875585 | 5 | 96257758 | C | 0.656 | 0.015 | 0.003 | 1.47E-08 | 0.023 | 0.013 | 8.19E-02 | 3.24E-01 |  |
| rs6888037 | 5 | 128070567 | T | 0.767 | 0.017 | 0.003 | 4.71E-09 | -0.014 | 0.014 | 3.15E-01 | 1.09E-01 |  |
| rs6893495 | 5 | 65113551 | T | 0.216 | 0.018 | 0.003 | 1.23E-09 | -0.001 | 0.015 | 9.62E-01 | 8.84E-01 |  |
| rs6927268 | 6 | 108544460 | T | 0.222 | -0.019 | 0.003 | 7.91E-10 | 0.002 | 0.015 | 9.02E-01 | 9.56E-01 |  |
| rs6948959 | 7 | 50629354 | G | 0.731 | -0.016 | 0.003 | 7.31E-09 | 0.014 | 0.014 | 3.10E-01 | 1.61E-01 |  |
| rs6977416 | 7 | 150845623 | G | 0.340 | -0.014 | 0.003 | 3.82E-08 | 0.011 | 0.013 | 4.02E-01 | 3.21E-03 |  |
| rs7020 | 20 | 25297964 | A | 0.443 | 0.018 | 0.002 | 7.63E-13 | -0.020 | 0.012 | 1.09E-01 | 3.30E-01 |  |
| rs7027304 | 9 | 126646011 | T | 0.667 | 0.014 | 0.003 | 3.73E-08 | 0.024 | 0.014 | 7.56E-02 | 5.67E-01 |  |
| rs704061 | 12 | 89378126 | C | 0.444 | 0.019 | 0.002 | 4.10E-15 | -0.001 | 0.012 | 9.51E-01 | 2.49E-01 |  |
| rs7046483 | 9 | 14777397 | G | 0.615 | -0.015 | 0.003 | 6.15E-09 | -0.022 | 0.013 | 7.49E-02 | 1.04E-02 |  |
| rs7094644 | 10 | 16731865 | A | 0.679 | 0.015 | 0.003 | 2.46E-08 | 0.017 | 0.013 | 1.96E-01 | 2.47E-01 |  |
| rs7117842 | 11 | 122663796 | C | 0.373 | 0.015 | 0.003 | 9.73E-09 | 0.010 | 0.013 | 4.35E-01 | 9.62E-01 |  |
| rs7124681 | 11 | 47508395 | A | 0.411 | 0.031 | 0.002 | 2.28E-35 | 0.004 | 0.012 | 7.30E-01 | 5.82E-01 |  |
| rs7133378 | 12 | 123924955 | A | 0.314 | 0.025 | 0.003 | 1.44E-21 | 0.004 | 0.013 | 7.34E-01 | 5.30E-02 |  |
| rs7135617 | 12 | 121631099 | G | 0.421 | 0.016 | 0.002 | 2.10E-10 | -0.001 | 0.012 | 9.35E-01 | 9.21E-01 |  |
| rs713586 | 2 | 24935139 | C | 0.477 | 0.032 | 0.002 | 1.32E-38 | -0.008 | 0.012 | 4.87E-01 | 4.18E-01 |  |
| rs7139583 | 13 | 98460976 | A | 0.287 | -0.020 | 0.003 | 4.71E-13 | 0.018 | 0.013 | 1.78E-01 | 2.21E-01 |  |
| rs7159203 | 14 | 101837300 | C | 0.068 | -0.025 | 0.005 | 4.99E-08 | -0.010 | 0.024 | 6.71E-01 | 1.96E-01 |  |
| rs7164727 | 15 | 72801650 | T | 0.668 | 0.020 | 0.003 | 4.35E-14 | -0.028 | 0.013 | 3.00E-02 | 9.92E-01 |  |
| rs71658797 | 1 | 77501822 | A | 0.273 | 0.029 | 0.004 | 1.71E-14 | -0.008 | 0.019 | 6.71E-01 | 3.35E-02 |  |
| rs7191938 | 16 | 71373627 | G | 0.710 | 0.015 | 0.003 | 8.79E-09 | 0.005 | 0.013 | 7.32E-01 | 3.37E-01 |  |
| rs7200589 | 16 | 299331 | G | 0.265 | -0.017 | 0.003 | 3.00E-10 | -0.001 | 0.014 | 9.18E-01 | 7.68E-01 |  |
| rs7206608 | 16 | 82839023 | G | 0.322 | 0.015 | 0.003 | 1.15E-08 | 0.017 | 0.014 | 2.15E-01 | 1.10E-01 |  |
| rs7238896 | 18 | 1840657 | G | 0.149 | 0.020 | 0.004 | 1.52E-08 | 0.015 | 0.017 | 3.93E-01 | 9.51E-01 |  |
| rs72755233 | 15 | 100152748 | G | 0.119 | -0.021 | 0.004 | 4.62E-08 | 0.043 | 0.056 | 4.47E-01 | 1.50E-01 |  |
| rs72820274 | 2 | 103796466 | A | 0.423 | 0.014 | 0.002 | 3.50E-08 | -0.005 | 0.013 | 6.73E-01 | 1.23E-01 |  |
| rs72917533 | 2 | 174374196 | T | 0.181 | -0.018 | 0.003 | 2.00E-08 | -0.011 | 0.016 | 4.85E-01 | 9.51E-01 |  |
| rs72961007 |  |  | C | 0.068 | -0.029 | 0.005 | 3.72E-09 | -0.027 | 0.030 | 3.78E-01 | 6.33E-01 |  |
| rs72976986 | 19 | 4050426 | G | 0.180 | -0.024 | 0.003 | 3.53E-14 | 0.025 | 0.017 | 1.53E-01 | 7.62E-01 |  |
| rs72995085 | 6 | 142872834 | T | 0.181 | -0.019 | 0.003 | 2.24E-09 | -0.012 | 0.016 | 4.61E-01 | 1.69E-01 |  |
| rs73021485 | 3 | 12285888 | G | 0.123 | -0.022 | 0.004 | 6.00E-09 | -0.001 | 0.019 | 9.44E-01 | 8.07E-01 |  |
| rs73041988 | 11 | 134647340 | T | 0.160 | -0.025 | 0.003 | 9.14E-14 | 0.031 | 0.017 | 5.89E-02 | 9.71E-01 |  |
| rs73213501 | 4 | 28513208 | A | 0.187 | -0.019 | 0.003 | 8.80E-09 | -0.046 | 0.016 | 3.99E-03 | 6.88E-01 |  |
| rs73236524 | 4 | 35036695 | A | 0.133 | 0.020 | 0.004 | 2.11E-08 | 0.005 | 0.018 | 7.85E-01 | 3.00E-02 |  |
| rs7412 | 19 | 44908822 | T | 0.076 | 0.027 | 0.005 | 1.13E-09 | -0.056 | 0.025 | 2.59E-02 | 1.34E-01 |  |
| rs7442885 | 5 | 88387060 | C | 0.211 | -0.019 | 0.003 | 1.32E-10 | 0.002 | 0.015 | 8.69E-01 | 3.93E-01 |  |
| rs7498044 | 15 | 92030409 | G | 0.225 | -0.017 | 0.003 | 9.67E-09 | 0.003 | 0.015 | 8.66E-01 | 5.02E-01 |  |
| rs7498798 | 16 | 73063130 | G | 0.649 | -0.015 | 0.003 | 8.69E-09 | -0.007 | 0.014 | 6.22E-01 | 7.85E-01 |  |
| rs7503604 |  |  | C | 0.509 | -0.014 | 0.002 | 3.19E-08 | 0.003 | 0.014 | 8.51E-01 | 7.61E-01 |  |
| rs7519259 | 1 | 65969060 | A | 0.524 | 0.014 | 0.002 | 8.27E-09 | 0.033 | 0.012 | 9.20E-03 | 1.67E-03 |  |
| rs7570258 | 2 | 192926994 | C | 0.508 | 0.014 | 0.002 | 3.30E-08 | 0.011 | 0.012 | 3.48E-01 | 1.97E-01 |  |
| rs7571753 | 2 | 50506820 | T | 0.398 | -0.014 | 0.003 | 2.02E-08 | -0.001 | 0.013 | 9.06E-01 | 4.37E-01 |  |
| rs75846784 | 5 | 125200984 | T | 0.081 | -0.026 | 0.005 | 2.22E-08 | -0.021 | 0.024 | 3.67E-01 | 1.38E-03 |  |
| rs75854315 | 1 | 156253265 | A | 0.060 | 0.029 | 0.005 | 5.99E-09 | 0.023 | 0.026 | 3.87E-01 | 3.05E-01 |  |
| rs7591494 | 2 | 211414215 | A | 0.261 | 0.017 | 0.003 | 1.47E-09 | -0.011 | 0.014 | 4.22E-01 | 9.75E-01 |  |
| rs7601895 | 2 | 55054765 | C | 0.299 | -0.015 | 0.003 | 8.23E-09 | 0.002 | 0.013 | 8.99E-01 | 2.35E-01 |  |
| rs76040172 | 21 | 45069044 | G | 0.057 | -0.039 | 0.005 | 7.13E-13 | 0.030 | 0.029 | 3.00E-01 | 8.40E-02 |  |
| rs76095247 | 15 | 66301130 | A | 0.248 | -0.016 | 0.003 | 2.48E-08 | 0.008 | 0.014 | 5.98E-01 | 8.88E-01 |  |
| rs7613261 | 3 | 131998951 | T | 0.206 | 0.022 | 0.003 | 1.54E-12 | -0.002 | 0.015 | 8.88E-01 | 5.57E-01 |  |
| rs7630228 | 3 | 71632336 | T | 0.443 | -0.015 | 0.002 | 2.65E-09 | -0.015 | 0.012 | 2.37E-01 | 7.62E-01 |  |
| rs7649970 | 3 | 12350773 | T | 0.132 | 0.039 | 0.004 | 1.18E-25 | -0.012 | 0.018 | 5.21E-01 | 2.97E-01 |  |
| rs76824303 | 3 | 62474144 | A | 0.108 | -0.023 | 0.004 | 3.98E-08 | 0.028 | 0.023 | 2.24E-01 | 5.95E-01 |  |
| rs7692075 | 4 | 77887515 | G | 0.156 | 0.020 | 0.003 | 9.45E-09 | -0.001 | 0.017 | 9.71E-01 | 6.48E-01 |  |
| rs7700107 | 4 | 17878793 | C | 0.126 | 0.020 | 0.004 | 3.94E-08 | 0.007 | 0.018 | 6.88E-01 | 3.67E-01 |  |
| rs7774 |  |  | A | 0.326 | 0.015 | 0.003 | 2.02E-08 | -0.044 | 0.016 | 4.91E-03 | 5.02E-01 |  |
| rs78265103 | 2 | 24245322 | A | 0.049 | -0.037 | 0.006 | 3.50E-11 | 0.009 | 0.029 | 7.47E-01 | 2.55E-01 |  |
| rs78801969 | 16 | 64673301 | C | 0.146 | -0.021 | 0.004 | 3.66E-09 | -0.019 | 0.018 | 2.86E-01 | 7.27E-01 |  |
| rs79113395 | 1 | 1659060 | G | 0.262 | -0.018 | 0.003 | 2.97E-10 | 0.018 | 0.076 | 8.15E-01 | 1.43E-01 |  |
| rs7925100 | 11 | 119070886 | A | 0.384 | 0.015 | 0.003 | 8.62E-10 | 0.012 | 0.013 | 3.53E-01 | 7.85E-01 |  |
| rs79478789 | 21 | 39181609 | C | 0.165 | 0.019 | 0.003 | 1.59E-08 | -0.025 | 0.017 | 1.50E-01 | 4.55E-01 |  |
| rs7953 | 7 | 77794257 | A | 0.475 | 0.014 | 0.002 | 1.56E-08 | -0.015 | 0.012 | 2.25E-01 | 3.39E-01 |  |
| rs7959140 | 12 | 107968357 | T | 0.774 | 0.019 | 0.003 | 1.41E-10 | -0.014 | 0.015 | 3.37E-01 | 2.61E-01 |  |
| rs7982447 | 13 | 53879676 | C | 0.206 | 0.021 | 0.003 | 3.52E-12 | 0.000 | 0.015 | 9.75E-01 | 7.68E-05 |  |
| rs79869125 | 2 | 175557510 | G | 0.104 | -0.023 | 0.004 | 1.82E-08 | 0.005 | 0.020 | 7.89E-01 | 8.78E-02 |  |
| rs79921461 | 2 | 158551986 | T | 0.155 | -0.019 | 0.003 | 3.13E-08 | 0.023 | 0.017 | 1.80E-01 | 6.58E-01 |  |
| rs80135947 | 17 | 67839885 | C | 0.209 | 0.031 | 0.003 | 3.58E-23 | 0.021 | 0.015 | 1.62E-01 | 6.65E-01 |  |
| rs8042404 | 15 | 31387813 | A | 0.270 | 0.016 | 0.003 | 1.17E-08 | -0.037 | 0.014 | 7.74E-03 | 7.07E-01 |  |
| rs8087074 | 18 | 48397111 | T | 0.257 | 0.017 | 0.003 | 6.00E-10 | 0.017 | 0.014 | 2.33E-01 | 7.34E-01 |  |
| rs809955 | 4 | 139953606 | G | 0.360 | -0.017 | 0.003 | 7.66E-12 | -0.009 | 0.013 | 4.96E-01 | 1.58E-01 |  |
| rs8112818 | 19 | 18701975 | A | 0.407 | -0.018 | 0.003 | 2.72E-12 | 0.008 | 0.013 | 5.61E-01 | 4.12E-01 |  |
| rs815163 | 1 | 190325596 | T | 0.553 | -0.014 | 0.002 | 1.74E-08 | -0.010 | 0.012 | 4.33E-01 | 8.19E-01 |  |
| rs815611 | 5 | 154139206 | G | 0.555 | -0.015 | 0.002 | 7.14E-10 | -0.019 | 0.012 | 1.25E-01 | 2.96E-01 |  |
| rs825501 | 12 | 124097669 | T | 0.631 | -0.016 | 0.003 | 5.72E-10 | 0.007 | 0.013 | 5.84E-01 | 1.12E-01 |  |
| rs878206 | 14 | 74797072 | G | 0.533 | 0.014 | 0.002 | 3.72E-08 | 0.014 | 0.012 | 2.70E-01 | 2.59E-02 |  |
| rs879620 | 16 | 3965728 | T | 0.608 | 0.022 | 0.003 | 6.80E-18 | -0.004 | 0.014 | 7.62E-01 | 6.71E-01 |  |
| rs9320823 | 6 | 97981461 | C | 0.617 | 0.023 | 0.003 | 8.81E-20 | 0.004 | 0.013 | 7.27E-01 | 3.50E-01 |  |
| rs9358657 | 6 | 23717956 | G | 0.733 | -0.015 | 0.003 | 3.87E-08 | -0.008 | 0.014 | 5.66E-01 | 3.87E-01 |  |
| rs9358912 | 6 | 26210918 | G | 0.298 | -0.032 | 0.003 | 6.17E-31 | -0.013 | 0.014 | 3.25E-01 | 7.13E-01 |  |
| rs9394307 | 6 | 35643455 | T | 0.338 | 0.017 | 0.003 | 5.58E-11 | 0.003 | 0.013 | 8.31E-01 | 4.66E-01 |  |
| rs9425633 | 1 | 184688117 | C | 0.484 | 0.014 | 0.002 | 5.39E-09 | 0.021 | 0.012 | 9.20E-02 | 9.79E-01 |  |
| rs9471333 | 6 | 40394284 | C | 0.536 | -0.022 | 0.002 | 9.25E-19 | -0.012 | 0.012 | 3.39E-01 | 1.62E-01 |  |
| rs9522279 | 13 | 111568949 | T | 0.431 | 0.018 | 0.002 | 5.58E-13 | -0.012 | 0.012 | 3.30E-01 | 7.76E-01 |  |
| rs9551991 | 13 | 19731852 | C | 0.134 | 0.021 | 0.004 | 1.02E-09 | 0.030 | 0.018 | 8.40E-02 | 7.30E-01 |  |
| rs9659073 | 1 | 150554878 | A | 0.487 | 0.015 | 0.002 | 6.47E-10 | 0.020 | 0.012 | 1.07E-01 | 5.97E-02 |  |
| rs972283 | 7 | 130782095 | A | 0.511 | -0.019 | 0.002 | 1.04E-14 | 0.011 | 0.012 | 3.61E-01 | 4.35E-03 |  |
| rs9747171 | 17 | 47133507 | G | 0.600 | 0.014 | 0.003 | 3.19E-08 | -0.013 | 0.012 | 3.09E-01 | 4.87E-01 |  |
| rs9788550 | 14 | 29211932 | G | 0.260 | -0.019 | 0.003 | 2.13E-11 | -0.019 | 0.014 | 1.94E-01 | 1.43E-01 |  |
| rs9803921 | 1 | 209063473 | C | 0.385 | -0.014 | 0.003 | 3.43E-08 | -0.007 | 0.013 | 5.79E-01 | 2.84E-01 |  |
| rs9843653 | 3 | 49883138 | C | 0.492 | 0.024 | 0.002 | 1.66E-22 | -0.021 | 0.012 | 8.78E-02 | 9.95E-01 |  |
| rs9865173 | 3 | 43975166 | T | 0.686 | -0.017 | 0.003 | 6.42E-10 | -0.018 | 0.013 | 1.76E-01 | 5.21E-01 |  |
| rs9902386 | 17 | 21376873 | T | 0.669 | -0.016 | 0.003 | 3.73E-10 | 0.024 | 0.014 | 8.63E-02 | 5.55E-02 |  |
| rs9906944 | 17 | 49014058 | C | 0.345 | -0.018 | 0.003 | 3.58E-12 | 0.042 | 0.015 | 4.32E-03 | 7.04E-01 |  |
| rs9948863 | 18 | 60301149 | G | 0.513 | 0.013 | 0.002 | 4.95E-08 | -0.015 | 0.012 | 2.15E-01 | 5.71E-01 |  |
| BFP: body fat percentage, CRC: colorectal cancer, EA: effect allele, EAF: effect allele frequency, associations between genetic variants and body fat percentage were extracted from Bycroft (2018), associations between BFP genetic variants and CRC risk were extracted from a meta-analysis of 15 primary CRC GWAS (Law 2019). | | | | | | | | | | | | |

| Table S9 Genetic variants associated with waist circumference recorded by Shungin (2015) | | | | | | | | |
| --- | --- | --- | --- | --- | --- | --- | --- | --- |
| rsid | Chr | Position | EA | EAF | Beta.WC | SE.WC | Beta.CRC | SE.CRC |
| rs10132280 | 14 | 25928179 | A | 0.333 | -0.022 | 0.004 | -0.008 | 0.012 |
| rs10511073 | 3 | 85653460 | G | 0.617 | -0.021 | 0.004 | 0.006 | 0.011 |
| rs10767658 | 11 | 27672252 | C | 0.358 | 0.031 | 0.004 | -0.005 | 0.012 |
| rs10840100 | 11 | 8669437 | G | 0.725 | 0.020 | 0.004 | 0.004 | 0.011 |
| rs10938397 | 4 | 45182527 | A | 0.567 | -0.032 | 0.004 | -0.011 | 0.011 |
| rs10968576 | 9 | 28414339 | G | 0.292 | 0.025 | 0.004 | 0.020 | 0.011 |
| rs11075986 | 16 | 53805344 | C | 0.900 | 0.038 | 0.007 | -0.049 | 0.019 |
| rs11165623 | 1 | 96893000 | A | 0.483 | 0.020 | 0.003 | 0.015 | 0.011 |
| rs11209963 | 1 | 72883303 | A | 0.178 | -0.028 | 0.004 | -0.014 | 0.014 |
| rs11873305 | 18 | 58049192 | A | 0.967 | 0.058 | 0.009 | -0.010 | 0.028 |
| rs12429545 | 13 | 54102206 | G | 0.900 | -0.031 | 0.005 | -0.016 | 0.016 |
| rs12446632 | 16 | 19935389 | A | 0.133 | -0.036 | 0.005 | -0.005 | 0.015 |
| rs12607795 | 18 | 57948098 | C | 0.225 | -0.025 | 0.004 | -0.014 | 0.013 |
| rs12885454 | 14 | 29736838 | C | 0.633 | 0.020 | 0.004 | 0.007 | 0.011 |
| rs12996547 | 2 | 602036 | C | 0.708 | -0.022 | 0.004 | 0.008 | 0.011 |
| rs1317006 | 2 | 656450 | C | 0.275 | 0.024 | 0.004 | 0.015 | 0.012 |
| rs1516725 | 3 | 185824004 | T | 0.092 | -0.031 | 0.005 | 0.006 | 0.016 |
| rs1549293 | 16 | 31141993 | T | 0.392 | -0.020 | 0.004 | -0.024 | 0.011 |
| rs1558902 | 16 | 53803574 | A | 0.450 | 0.074 | 0.004 | -0.001 | 0.011 |
| rs16894959 | 6 | 34825662 | C | 0.100 | 0.026 | 0.005 | -0.030 | 0.015 |
| rs16996700 | 20 | 50981945 | T | 0.700 | 0.023 | 0.004 | 0.005 | 0.012 |
| rs17391694 | 1 | 78623626 | T | 0.136 | 0.036 | 0.006 | -0.036 | 0.016 |
| rs1943226 | 18 | 58035204 | T | 0.875 | -0.034 | 0.006 | 0.004 | 0.019 |
| rs2112347 | 5 | 75015242 | G | 0.375 | -0.025 | 0.004 | -0.011 | 0.011 |
| rs2287019 | 19 | 46202172 | C | 0.850 | 0.035 | 0.005 | -0.005 | 0.014 |
| rs2293576 | 11 | 47434986 | A | 0.367 | -0.022 | 0.004 | -0.018 | 0.011 |
| rs2325036 | 3 | 85819412 | A | 0.592 | 0.023 | 0.004 | -0.010 | 0.011 |
| rs2370983 | 14 | 79903376 | G | 0.233 | -0.020 | 0.004 | -0.011 | 0.011 |
| rs2489623 | 6 | 127455821 | C | 0.558 | 0.019 | 0.003 | -0.002 | 0.011 |
| rs2531992 | 16 | 4021734 | A | 0.167 | -0.028 | 0.005 | -0.033 | 0.016 |
| rs2635727 | 6 | 50820940 | C | 0.758 | 0.025 | 0.004 | -0.003 | 0.012 |
| rs3127553 | 1 | 49438005 | G | 0.367 | 0.023 | 0.004 | 0.009 | 0.011 |
| rs3810291 | 19 | 47569003 | A | 0.625 | 0.026 | 0.004 | 0.003 | 0.012 |
| rs3849570 | 3 | 81792112 | A | 0.367 | 0.021 | 0.004 | 0.016 | 0.011 |
| rs4074134 | 11 | 27647285 | C | 0.800 | 0.032 | 0.004 | 0.050 | 0.013 |
| rs4130548 | 1 | 78463868 | C | 0.425 | 0.022 | 0.004 | -0.001 | 0.011 |
| rs4611674 | 2 | 648899 | G | 0.567 | -0.020 | 0.004 | -0.021 | 0.011 |
| rs4776970 | 15 | 68080886 | T | 0.342 | -0.020 | 0.004 | -0.016 | 0.011 |
| rs6163 | 10 | 104596924 | C | 0.608 | -0.019 | 0.004 | 0.006 | 0.011 |
| rs633715 | 1 | 177852580 | C | 0.267 | 0.043 | 0.004 | -0.009 | 0.013 |
| rs6440003 | 3 | 141094209 | G | 0.517 | -0.021 | 0.003 | -0.015 | 0.011 |
| rs6499653 | 16 | 53877592 | T | 0.225 | 0.022 | 0.004 | -0.016 | 0.013 |
| rs6545714 | 2 | 59307725 | G | 0.375 | 0.022 | 0.004 | 0.008 | 0.011 |
| rs6567160 | 18 | 57829135 | C | 0.283 | 0.048 | 0.004 | 0.022 | 0.013 |
| rs6755502 | 2 | 635721 | T | 0.125 | -0.051 | 0.005 | -0.020 | 0.014 |
| rs7138803 | 12 | 50247468 | G | 0.558 | -0.028 | 0.004 | -0.002 | 0.011 |
| rs7144011 | 14 | 79940383 | T | 0.275 | 0.033 | 0.004 | 0.004 | 0.013 |
| rs7186521 | 16 | 53792922 | G | 0.492 | 0.030 | 0.003 | 0.010 | 0.011 |
| rs7203521 | 16 | 53769293 | A | 0.650 | 0.030 | 0.004 | 0.026 | 0.011 |
| rs7239883 | 18 | 40147671 | G | 0.317 | 0.021 | 0.004 | 0.003 | 0.011 |
| rs7498665 | 16 | 28883241 | G | 0.358 | 0.034 | 0.004 | -0.006 | 0.011 |
| rs7531118 | 1 | 72837239 | T | 0.392 | -0.027 | 0.004 | -0.014 | 0.011 |
| rs7550711 | 1 | 110082886 | T | 0.034 | 0.058 | 0.010 | 0.008 | 0.033 |
| rs7903146 | 10 | 114758349 | T | 0.250 | -0.022 | 0.004 | 0.006 | 0.012 |
| rs806794 | 6 | 26200677 | G | 0.275 | -0.022 | 0.004 | -0.012 | 0.012 |
| rs929641 | 2 | 58792377 | A | 0.617 | 0.021 | 0.003 | 0.002 | 0.011 |
| rs9400239 | 6 | 108977663 | C | 0.700 | 0.024 | 0.004 | 0.015 | 0.012 |
| rs943005 | 6 | 50865820 | T | 0.100 | 0.039 | 0.004 | 0.006 | 0.014 |
| rs9956279 | 18 | 57942799 | T | 0.350 | 0.035 | 0.004 | 0.001 | 0.012 |
| WC: waist circumference, CRC: colorectal cancer, EA: effect allele, EAF: effect allele frequency, associations between genetic variants and waist circumference were extracted from Shungin (2015), associations between waist circumference genetic variants and CRC risk were extracted from a meta-analysis of 15 primary CRC GWAS (Law 2019). | | | | | | | | |

| Table S10 Genetic variants associated with body fat distribution | | | | | | | | | | | | | |
| --- | --- | --- | --- | --- | --- | --- | --- | --- | --- | --- | --- | --- | --- |
| rsid | Category | Chr | Position | EA | EAF | Beta.AFR | SE.AFR | Beta.LFR | SE.LFR | Beta.TFR | SE.TFR | Beta.CRC | SE.CRC |
| rs1138714 | AFR | 9 | 78510823 | A | 0.119 | 0.003 | 0.013 | 0.036 | 0.011 | -0.035 | 9.04E-08 | 0.004 | 0.012 |
| rs12971970 | AFR | 19 | 47559273 | T | 0.251 | -0.028 | 0.008 | 0.004 | 0.010 | 0.006 | 0.203 | 0.011 | 0.018 |
| rs13011472 | AFR | 2 | 57961602 | G | 0.489 | -0.024 | 0.007 | 0.007 | 0.008 | 0.003 | 0.560 | -0.005 | 0.011 |
| rs1789166 | AFR | 11 | 69482091 | C | 0.352 | -0.027 | 0.007 | 0.010 | 0.008 | 4.85E-04 | 0.913 | -0.028 | 0.011 |
| rs2044387 | AFR | 11 | 825110 | A | 0.432 | -0.024 | 0.007 | 0.002 | 0.009 | 0.007 | 0.121 | -0.002 | 0.011 |
| rs2635727 | AFR | 6 | 50820940 | T | 0.242 | -0.029 | 0.008 | 0.007 | 0.009 | 0.004 | 0.434 | 0.003 | 0.012 |
| rs34341 | AFR | 5 | 74934009 | A | 0.423 | -0.024 | 0.007 | 0.006 | 0.008 | 0.003 | 0.555 | -0.015 | 0.011 |
| rs351855 | AFR | 5 | 176520243 | A | 0.296 | 0.029 | 0.007 | 0.002 | 0.009 | -0.012 | 0.010 | 0.016 | 0.012 |
| rs4971091 | AFR | 15 | 100800840 | A | 0.325 | -0.008 | 0.008 | -0.015 | 0.008 | 0.018 | 5.28E-05 | -0.005 | 0.011 |
| rs4980661 | AFR | 11 | 69306579 | G | 0.474 | 0.024 | 0.007 | -0.008 | 0.008 | -0.002 | 0.572 | 0.009 | 0.013 |
| rs56282717 | AFR | 12 | 123051018 | G | 0.343 | 0.007 | 0.008 | -0.023 | 0.007 | 0.019 | 3.71E-05 | 0.022 | 0.013 |
| rs6731872 | AFR | 2 | 624205 | T | 0.168 | -0.047 | 0.009 | 0.006 | 0.011 | 0.014 | 0.011 | -0.018 | 0.014 |
| rs7306275 | AFR | 12 | 50250111 | A | 0.368 | 0.025 | 0.007 | -0.018 | 0.007 | 0.008 | 0.077 | 0.003 | 0.011 |
| rs754537 | AFR | 16 | 31104509 | G | 0.377 | -0.024 | 0.007 | 0.002 | 0.009 | 0.005 | 0.218 | -0.017 | 0.011 |
| rs8050894 | AFR | 7 | 28207300 | G | 0.295 | -0.019 | 0.008 | -0.016 | 0.008 | 0.024 | 2.90E-07 | -0.014 | 0.011 |
| rs10769282 | AFR, TFR, LFR | 11 | 47626492 | G | 0.291 | -0.022 | 0.008 | 0.026 | 0.007 | -0.017 | 2.20E-04 | -0.008 | 0.012 |
| rs11856122 | AFR, TFR, LFR | 15 | 84576348 | G | 0.478 | 0.026 | 0.007 | 0.021 | 0.007 | -0.031 | 3.68E-13 | 0.015 | 0.011 |
| rs12905253 | AFR, TFR, LFR | 15 | 74232437 | A | 0.470 | 0.016 | 0.007 | 0.018 | 0.007 | -0.023 | 4.43E-08 | -0.003 | 0.011 |
| rs3812049 | AFR, LFR | 5 | 127418850 | C | 0.256 | 0.033 | 0.008 | -0.023 | 0.008 | 0.010 | 0.037 | 0.014 | 0.014 |
| rs545608 | AFR, LFR | 1 | 177899121 | C | 0.208 | 0.038 | 0.008 | -0.021 | 0.009 | 0.007 | 0.206 | -0.011 | 0.013 |
| rs11856122 | AFR, TFR | 15 | 84576348 | G | 0.478 | 0.026 | 0.007 | 0.021 | 0.007 | -0.031 | 3.68E-13 | 0.015 | 0.011 |
| rs1986599 | AFR, TFR | 3 | 50034637 | G | 0.113 | 0.020 | 0.012 | 0.028 | 0.011 | -0.037 | 4.28E-08 | -0.017 | 0.017 |
| rs55872725 | AFR, TFR | 16 | 53809123 | T | 0.402 | 0.056 | 0.006 | -0.006 | 0.008 | -0.018 | 2.28E-05 | -0.001 | 0.011 |
| rs28394864 | AFR, TFR, LFR | 8 | 8907950 | A | 0.425 | 0.018 | 0.007 | 0.002 | 0.009 | -0.010 | 0.031 | -0.025 | 0.011 |
| rs3817428 | AFR, TFR, LFR | 15 | 89415247 | G | 0.269 | 0.021 | 0.008 | 0.031 | 0.007 | -0.038 | 2.30E-15 | -0.014 | 0.013 |
| rs481806 | AFR, TFR, LFR | 18 | 20724328 | G | 0.217 | 0.007 | 0.009 | 0.016 | 0.009 | -0.018 | 0.001 | 0.002 | 0.012 |
| rs72755233 | AFR, TFR, LFR | 15 | 100692953 | A | 0.113 | 0.030 | 0.011 | 0.034 | 0.011 | -0.044 | 6.43E-11 | -0.035 | 0.031 |
| rs1317415 | LFR | 15 | 67457698 | G | 0.059 | -0.020 | 0.016 | -0.030 | 0.015 | 0.039 | 1.86E-05 | 0.003 | 0.012 |
| rs6489111 | LFR | 6 | 142679572 | A | 0.283 | 0.012 | 0.008 | 0.021 | 0.008 | -0.025 | 7.66E-08 | -0.013 | 0.011 |
| rs10916174 | TFR | 4 | 82165790 | C | 0.308 | -0.009 | 0.008 | -0.018 | 0.008 | 0.021 | 6.71E-06 | 0.001 | 0.015 |
| rs2241069 | TFR | 7 | 20442796 | C | 0.412 | 0.007 | 0.008 | 0.019 | 0.007 | -0.021 | 1.38E-06 | 0.023 | 0.012 |
| rs2273368 | TFR | 1 | 227804041 | A | 0.154 | 0.027 | 0.010 | 0.012 | 0.010 | -0.022 | 1.49E-04 | -0.021 | 0.013 |
| rs2982708 | TFR | 4 | 8602798 | G | 0.461 | -0.010 | 0.007 | -0.016 | 0.007 | 0.021 | 1.41E-06 | 0.007 | 0.012 |
| rs4800148 | TFR | 8 | 130731484 | T | 0.489 | 0.007 | 0.008 | 0.018 | 0.007 | -0.020 | 2.81E-06 | 5.20E-05 | 0.013 |
| rs79334166 | TFR | 9 | 86639999 | G | 0.248 | 0.001 | 0.010 | -0.024 | 0.008 | 0.025 | 5.61E-07 | 0.024 | 0.016 |
| rs10402308 | TFR, LFR | 19 | 19657500 | A | 0.177 | -0.008 | 0.010 | 0.035 | 0.009 | -0.031 | 2.03E-08 | 0.004 | 0.014 |
| rs10962638 | TFR, LFR | 9 | 16846111 | A | 0.142 | 0.005 | 0.012 | 0.035 | 0.010 | -0.037 | 4.22E-09 | 0.014 | 0.017 |
| rs11049566 | TFR, LFR | 12 | 28532961 | T | 0.290 | 0.012 | 0.008 | 0.026 | 0.007 | -0.028 | 1.36E-09 | -0.029 | 0.012 |
| rs11205303 | TFR, LFR | 1 | 149906413 | C | 0.409 | -0.015 | 0.007 | -0.044 | 0.007 | 0.048 | 1.74E-28 | -0.006 | 0.011 |
| rs112416074 | TFR, LFR | 7 | 148647760 | AG | 0.169 | -0.007 | 0.010 | -0.029 | 0.009 | 0.032 | 2.07E-08 | 0.007 | 0.018 |
| rs143384 | TFR, LFR | 20 | 34025756 | G | 0.402 | 0.010 | 0.008 | -0.038 | 0.007 | 0.034 | 1.62E-15 | -0.003 | 0.011 |
| rs17511102 | TFR, LFR | 2 | 37960613 | T | 0.090 | -0.021 | 0.013 | -0.034 | 0.012 | 0.042 | 1.10E-08 | 0.010 | 0.026 |
| rs2071167 | TFR, LFR | 17 | 42287519 | T | 0.235 | -0.005 | 0.009 | -0.028 | 0.008 | 0.029 | 7.59E-09 | -0.014 | 0.013 |
| rs2074188 | TFR, LFR | 17 | 45888251 | G | 0.476 | -0.001 | 0.009 | -0.023 | 0.007 | 0.023 | 4.73E-08 | -0.006 | 0.011 |
| rs2492863 | TFR, LFR | 6 | 34603646 | A | 0.138 | 0.012 | 0.011 | -0.035 | 0.010 | 0.029 | 2.52E-06 | -0.030 | 0.016 |
| rs2820443 | TFR, LFR | 5 | 157952404 | C | 0.307 | 0.002 | 0.009 | -0.017 | 0.008 | 0.016 | 4.03E-04 | 0.025 | 0.012 |
| rs314263 | TFR, LFR | 6 | 105392745 | C | 0.320 | -0.012 | 0.008 | -0.029 | 0.007 | 0.033 | 3.99E-13 | 0.001 | 0.012 |
| rs35344761 | TFR, LFR | 1 | 219753509 | C | 0.299 | 0.004 | 0.009 | -0.025 | 0.007 | 0.022 | 3.16E-06 | -0.003 | 0.017 |
| rs35874463 | TFR, LFR | 17 | 47450775 | A | 0.462 | 0.011 | 0.007 | 0.018 | 0.007 | -0.022 | 1.42E-07 | 0.032 | 0.026 |
| rs3780327 | TFR, LFR | 9 | 129945847 | A | 0.220 | 0.008 | 0.009 | -0.031 | 0.008 | 0.028 | 8.70E-08 | -0.020 | 0.015 |
| rs3791679 | TFR, LFR | 2 | 56096892 | G | 0.226 | 0.006 | 0.009 | 0.030 | 0.008 | -0.031 | 8.73E-10 | -0.018 | 0.013 |
| rs3823974 | TFR, LFR | 6 | 152356220 | C | 0.271 | -0.015 | 0.008 | -0.010 | 0.008 | 0.016 | 8.44E-04 | 0.011 | 0.011 |
| rs41271299 | TFR, LFR | 6 | 19839415 | T | 0.052 | -0.017 | 0.017 | -0.047 | 0.015 | 0.055 | 9.83E-09 | 0.014 | 0.034 |
| rs4521268 | TFR, LFR | 3 | 49137904 | G | 0.333 | 0.007 | 0.008 | 0.023 | 0.007 | -0.025 | 4.30E-08 | 0.003 | 0.011 |
| rs465983 | TFR, LFR | 5 | 55812130 | G | 0.235 | -0.007 | 0.009 | -0.026 | 0.008 | 0.029 | 6.75E-09 | -0.020 | 0.013 |
| rs4694504 | TFR, LFR | 4 | 73496691 | G | 0.473 | -0.015 | 0.007 | -0.018 | 0.007 | 0.022 | 2.99E-07 | 0.018 | 0.011 |
| rs4733727 | TFR, LFR | 1 | 17306870 | G | 0.477 | 0.007 | 0.008 | 0.020 | 0.007 | -0.023 | 6.94E-08 | -0.005 | 0.011 |
| rs4846204 | TFR, LFR | 1 | 10308958 | T | 0.127 | -0.008 | 0.012 | -0.032 | 0.010 | 0.035 | 2.87E-08 | 0.011 | 0.016 |
| rs4988781 | TFR, LFR | 7 | 150657095 | A | 0.243 | -0.027 | 0.008 | 0.000 | 0.010 | 0.010 | 4.81E-02 | 0.003 | 0.012 |
| rs55750792 | TFR, LFR | 1 | 184009826 | GAA | 0.345 | -0.004 | 0.009 | -0.024 | 0.007 | 0.024 | 6.75E-08 | 0.003 | 0.015 |
| rs5779197 | TFR, LFR | 13 | 91993746 | A | 0.221 | -0.005 | 0.010 | -0.020 | 0.009 | 0.020 | 9.86E-05 | -0.020 | 0.013 |
| rs6570507 | TFR, LFR | 4 | 17859466 | G | 0.129 | 0.005 | 0.012 | 0.023 | 0.010 | -0.025 | 8.54E-05 | 0.016 | 0.012 |
| rs7039458 | TFR, LFR | 15 | 89361669 | A | 0.472 | -0.007 | 0.008 | -0.019 | 0.007 | 0.020 | 1.39E-06 | -0.026 | 0.012 |
| rs71420186 | TFR, LFR | 14 | 50960918 | A | 0.066 | 0.029 | 0.014 | -0.050 | 0.014 | 0.036 | 2.55E-05 | 0.041 | 0.022 |
| rs7236575 | TFR, LFR | 18 | 46653380 | A | 0.136 | 0.011 | 0.011 | 0.035 | 0.010 | -0.039 | 2.14E-10 | -0.014 | 0.016 |
| rs7680661 | TFR, LFR | 4 | 145565116 | G | 0.169 | 0.007 | 0.010 | 0.033 | 0.009 | -0.035 | 5.92E-10 | -0.009 | 0.014 |
| rs888762 | TFR, LFR | 5 | 178547313 | C | 0.330 | -0.009 | 0.008 | -0.023 | 0.007 | 0.026 | 6.32E-09 | 0.003 | 0.012 |
| rs9358913 | TFR, LFR | 6 | 26239404 | G | 0.258 | 0.002 | 0.010 | 0.028 | 0.008 | -0.028 | 8.44E-09 | -0.011 | 0.012 |
| rs9853018 | TFR, LFR | 3 | 141101961 | T | 0.446 | 0.002 | 0.008 | -0.029 | 0.007 | 0.028 | 3.23E-11 | 0.015 | 0.011 |
| rs991967 | TFR, LFR | 1 | 218615451 | C | 0.286 | -0.001 | 0.009 | -0.026 | 0.007 | 0.026 | 4.76E-08 | 0.001 | 0.012 |
| rs994014 | TFR, LFR | 12 | 576037 | C | 0.351 | -0.003 | 0.009 | -0.022 | 0.007 | 0.024 | 1.22E-07 | -3.76E-04 | 0.012 |
| AFR: arm fat ratio, TFR: trunk fat ratio, LFR: leg fat ratio, CRC: colorectal cancer, EA: effect allele, EAF: effect allele frequency | | | | | | | | | | | | | |
| Associations between genetic variants and body fat distribution were extracted from Rask-Andersen (2019), associations between body fat distribution genetic variants and CRC risk were extracted from a meta-analysis of 15 primary CRC GWAS (Law 2019). | | | | | | | | | | | | | |

| Table S11 Results of network analysis | | | | | | | | | | | | | | | | | | |
| --- | --- | --- | --- | --- | --- | --- | --- | --- | --- | --- | --- | --- | --- | --- | --- | --- | --- | --- |
|  | Effect of MVPA on BMI | | | | Effect of AMPA at P<5×10^-8^  (5 SNPs) on BMI | | | |  | Effect of AMPA at P<5×10^-9^  (3 SNPs) on BMI | | | | Effect of BMI on CRC | | | | |
|  | Causal effects (95%CI) | P | P_int_^a^ | P_het_^b^ | Causal effects (95%CI) | P | P_int_^a^ | P_het_^b^ |  | Causal effects (95%CI) | P | P_int_^a^ | P_het_^b^ | Causal effects (95%CI) | P | P_int_^a^ | P_het_^b^ |  |
| **Main analysis** | | | | | | | | | | | | | | | | | | |
| IVW | 1.02 (0.76,1.38) | 0.88 | / | 0.009 | 0.81 (0.63,1.03) | 0.09 | / | 0.007 |  | 0.80 (0.69,0.94) | 0.005 | / | 0.005 | 1.24 (1.11, 1.39) | 0.0001 | / | 0.16 |  |
| **Sensitivity analysis** | | | | | | | | | | | | | | | | | | |
| MBE | 0.89 (0.69,1.14) | 0.35 | / | / | 0.68 (0.50, 0.93) | 0.02 | / | / |  | 0.66 (0.44,0.99) | 0.05 | / | / | 1.12 (0.91, 1.38) | 0.28 | / | / |  |
| Weighted median | 0.90 (0.70,1.16) | 0.40 | / | / | 0.71 (0.58,0.86) | 0.0006 | / | / |  | 0.73 (0.58,0.92) | 0.007 | / | / | 1.17 (1.00, 1.36) | 0.05 | / | / |  |
| MR-Egger | 0.60 (0.23,1.53) | 0.28 | 0.23 | 0.02 | 3.75 (0.81,17.43) | 0.09 | 0.09 | 0.11 |  | 5.62 (1.71,18.44) | 0.004 | 0.001 | 0.73 | 1.09 (0.84, 1.41) | 0.53 | 0.28 | 0.17 |  |
| MR-Robust | 0.89 (0.48,1.64) | 0.71 | / | / | 0.80 (0.58,1.09) | 0.15 | / | / |  | 0.80 (0.71,0.90) | 0.0002 | / | / | 1.22 (1.08, 1.38) | 0.001 | / | / |  |
| MR-PRESSO | 1.02 (0.76,1.38) | 0.88 | / | / | 0.81 (0.63,1.03) | 0.09 | / | / |  | / | / | / | / | 1.24 (1.11, 1.39) | 0.0001 | / | / |  |
| MVPA: self-reported moderate-to-vigorous physical activity, AMPA: acceleration vector magnitude physical activity, BMI: body mass index, CI: confidence interval, P_int_^a^: P-value for the intercept of MR-Egger’s test, P_het_^b^: P values of χ2 Q test for heterogeneity, IVW: inverse variance-weighted, MBE: mode-based estimate, MR-PRESSO: MR–Pleiotropy Residual Sum and Outlier. | | | | | | | | | | | | | | | | | | |

| Table S12 Time spend on physical activity to achieve risk reduction of colorectal cancer | | | |
| --- | --- | --- | --- |
| Physical activity type | Approximate MET intensity (METs/hour) | *Time spend on MVPA to achieve 44% decreases of CRC risk (minutes) | *Time spend on AMPA to achieve 40% decreases of CRC risk (minutes) |
| Walking briskly (3 mph) | 3.3 | 90 | 55 |
| Casual bicycling | 4 | 74 | 45 |
| Swimming | 8 | 37 | 23 |
| Running (8 mph) | 13.5 | 22 | 13 |
| Aerobic calisthenics | 6-10 | 30-50 | 18-30 |
| CRC: colorectal cancer, MVPA: self-reported moderate-to-vigorous physical activity, AMPA: acceleration vector magnitude physical activity, MET: metabolic equivalent task, *: time spend for individuals replace daily sedentary behaviour with MVPA or AMPA to achieve a decrease of CRC risk. | | | |

# Supplementary figures

## Figure S1 Visualisation of Mendelian randomisation analysis of the effect of self-reported moderate-to-vigorous physical activity (MVPA), acceleration vector magnitude physical activity (AMPA) and sedentary time on colorectal cancer risk

A: scatter plot of MVPA on colorectal cancer risk; B: scatter plot of AMPA on colorectal cancer risk (P<5×10^-8^); C: scatter plot of accelerometer measured PA on colorectal cancer risk (P<5×10^-9^); D: scatter plot of sedentary time on colorectal cancer risk

A


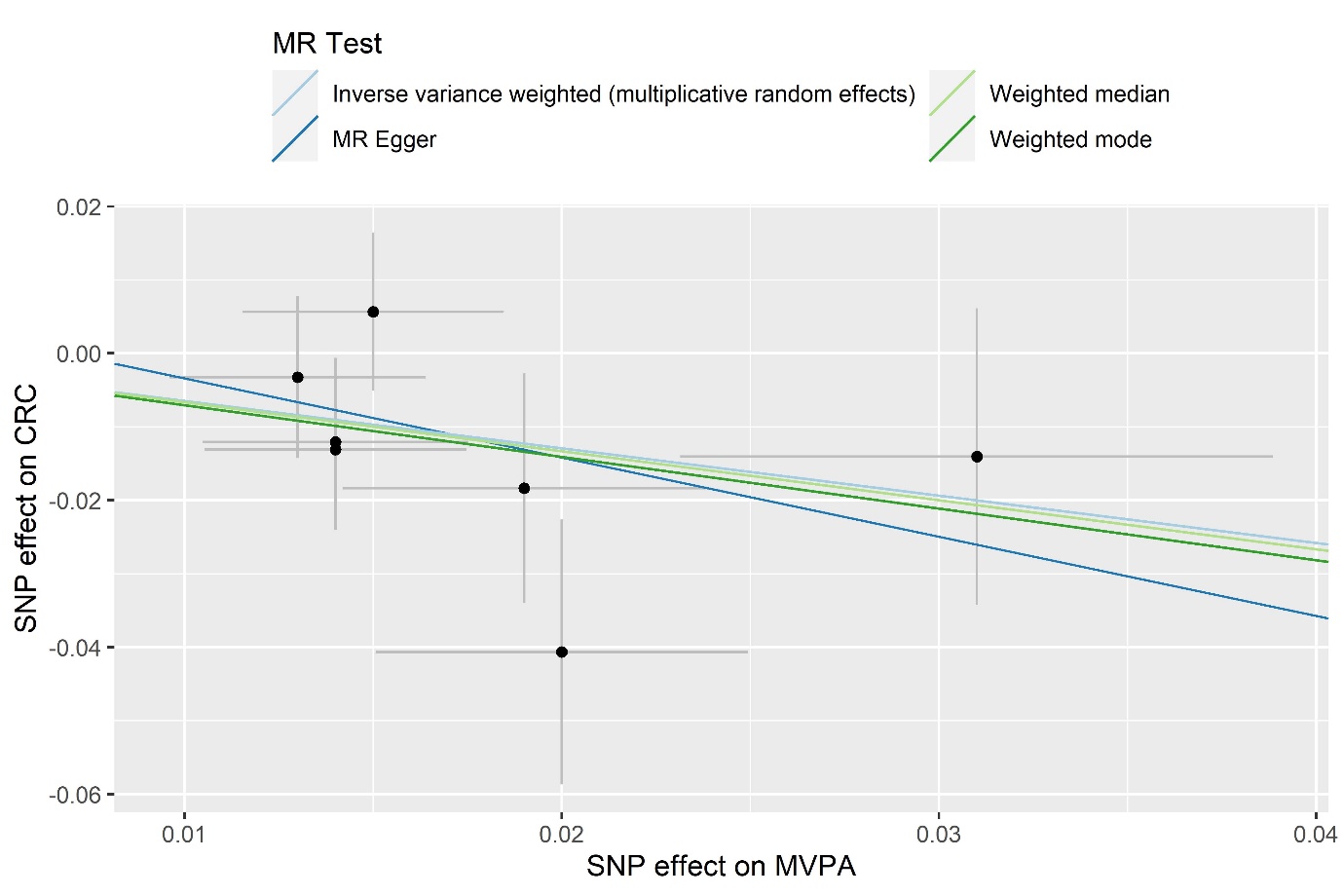


B
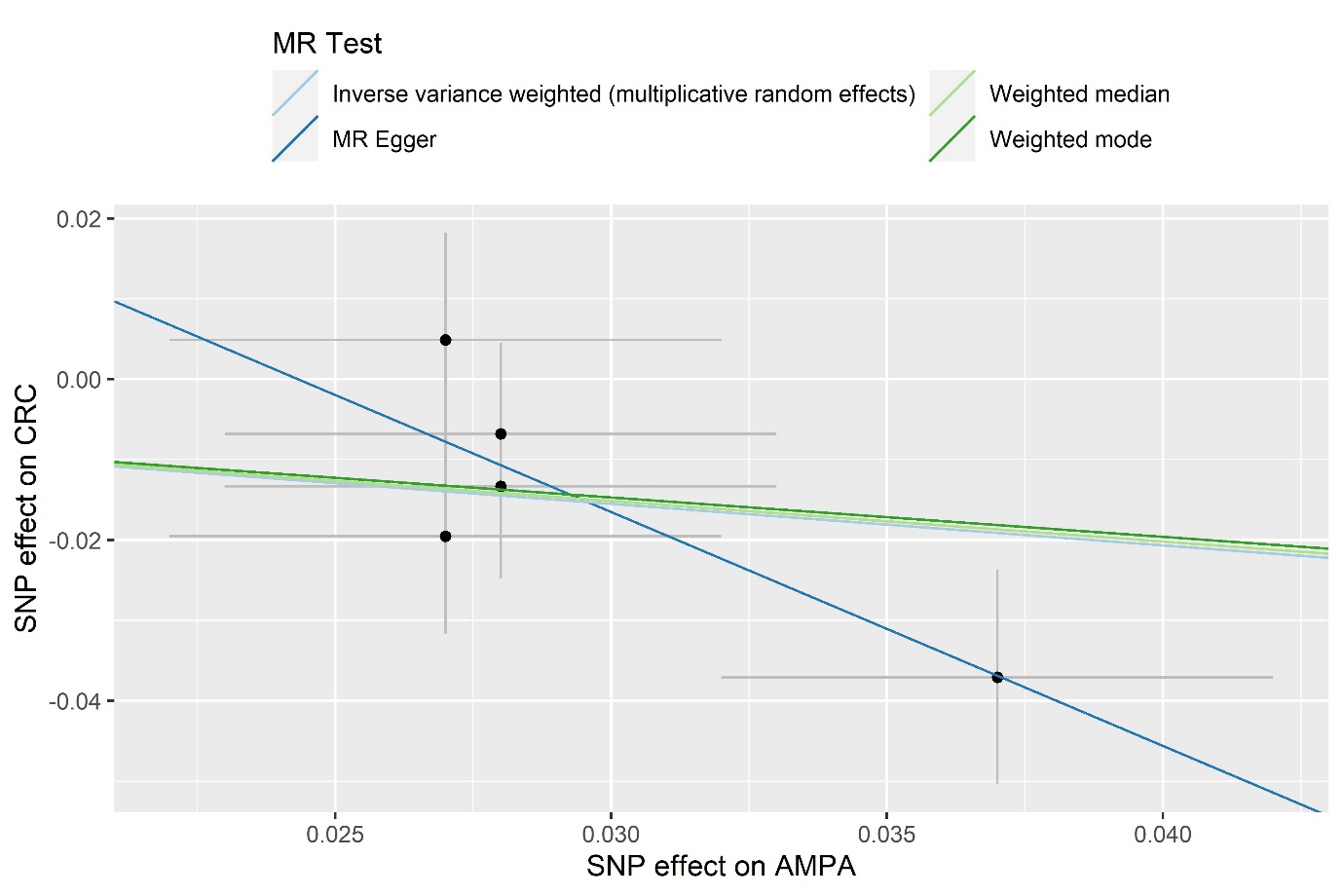


C


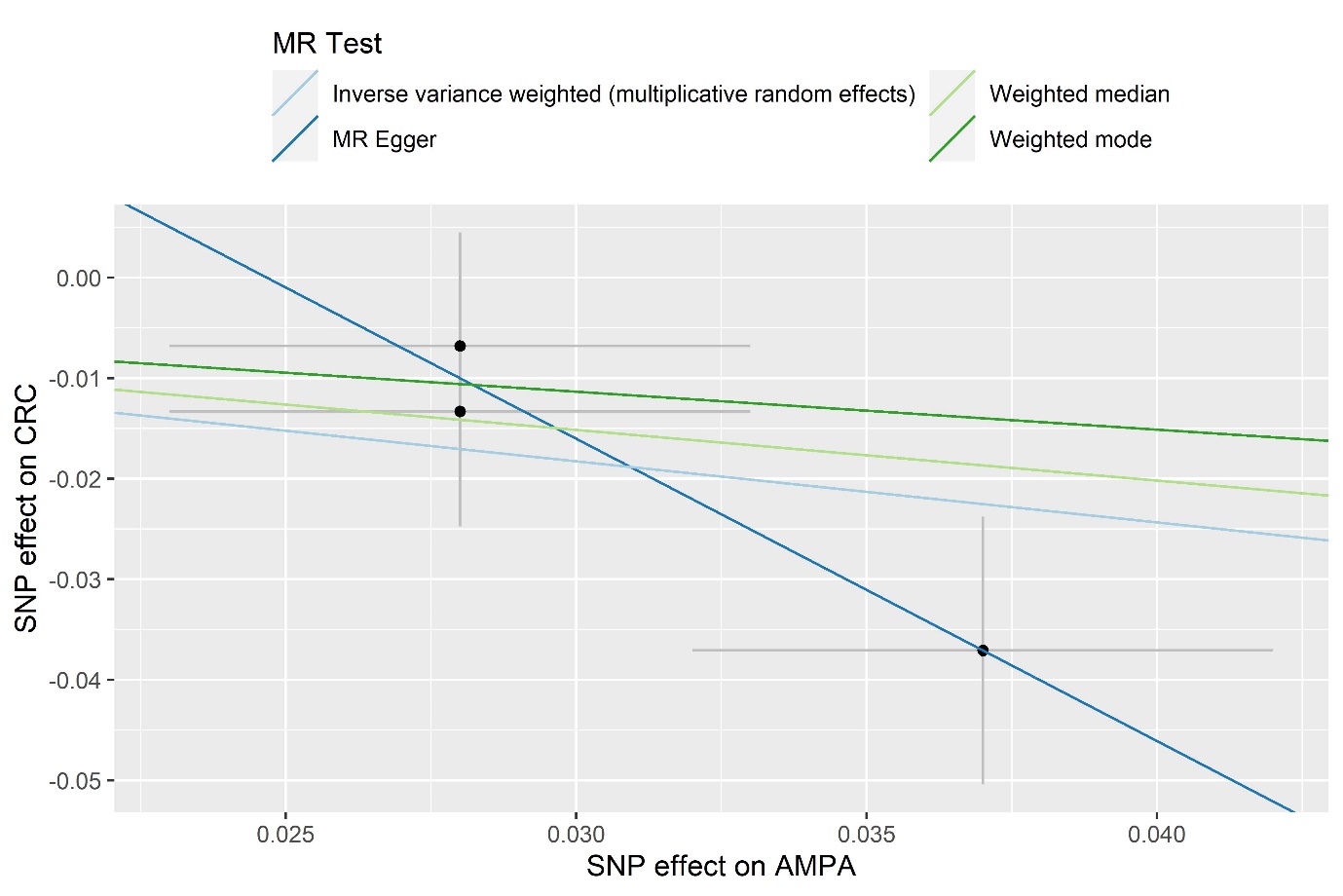


D

**
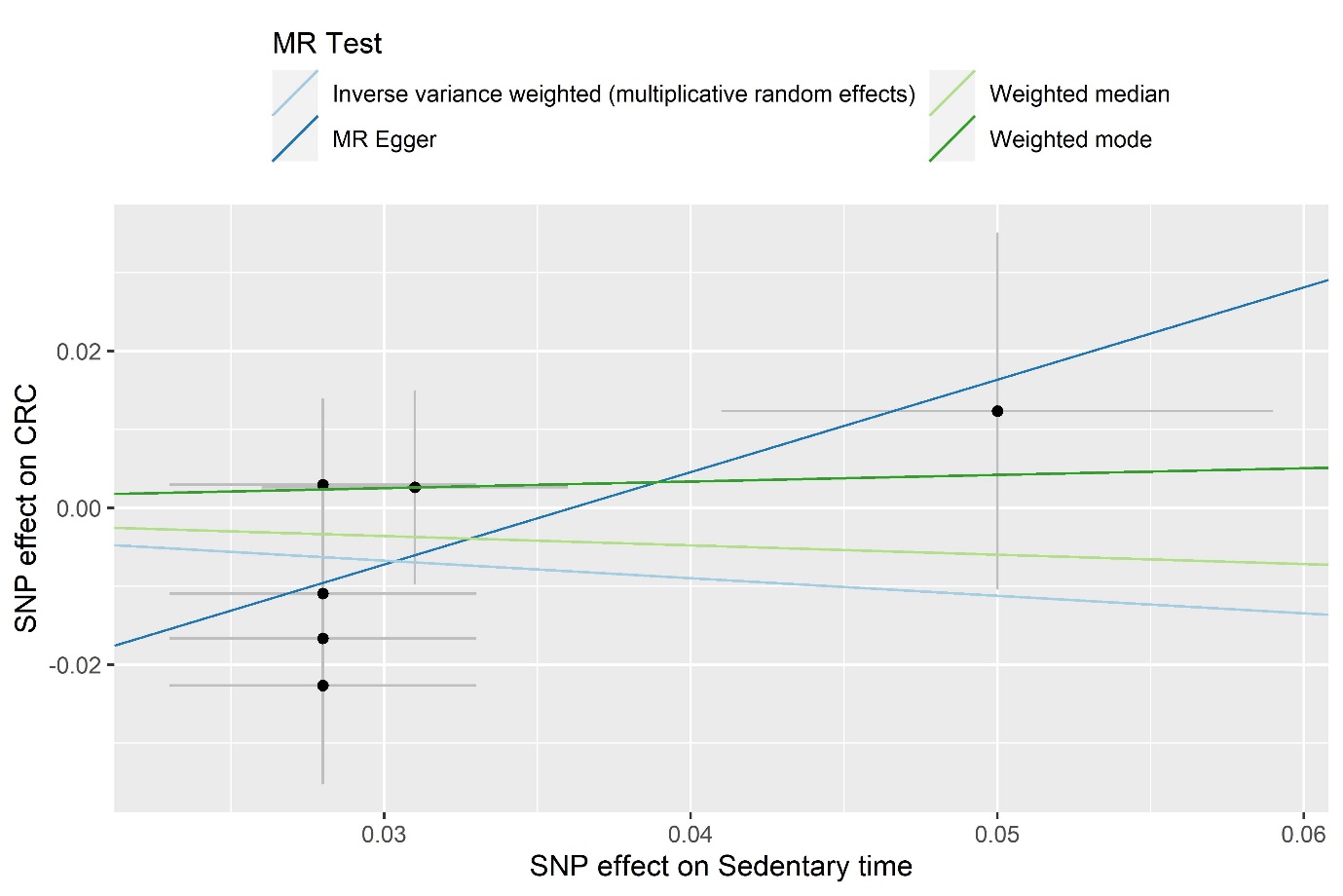
**

## Figure S2 Results of Mendelian randomisation (MR) analysis using MR-PRESSO and leave-one-out methods for the association between self-reported moderate-to-vigorous physical activity (MVPA) and colorectal cancer (CRC) risk

A: scatter plot of MR result, B: forest plot of MR of each MVPA SNP on CRC risk

**A**

**
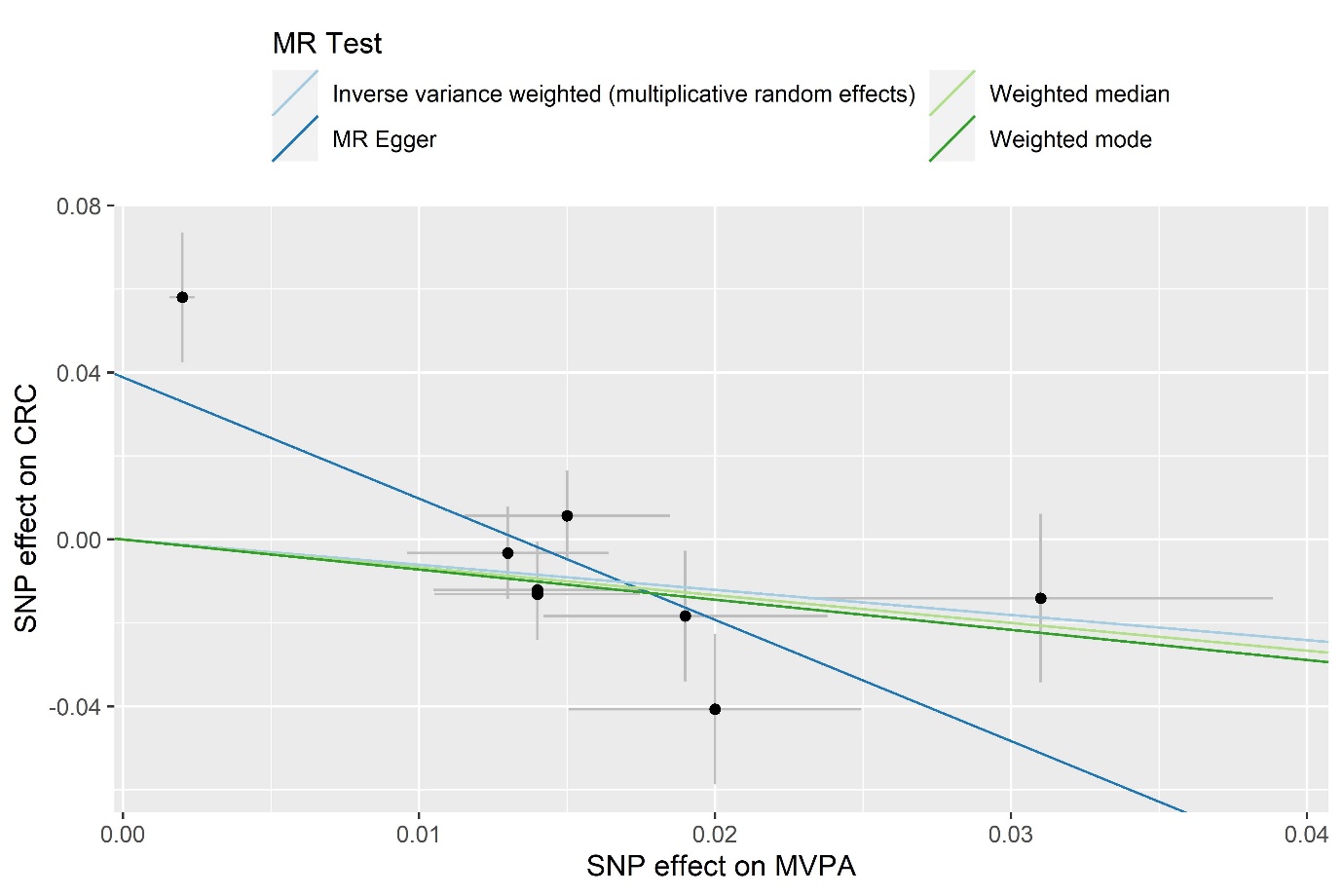
**

**B**

**
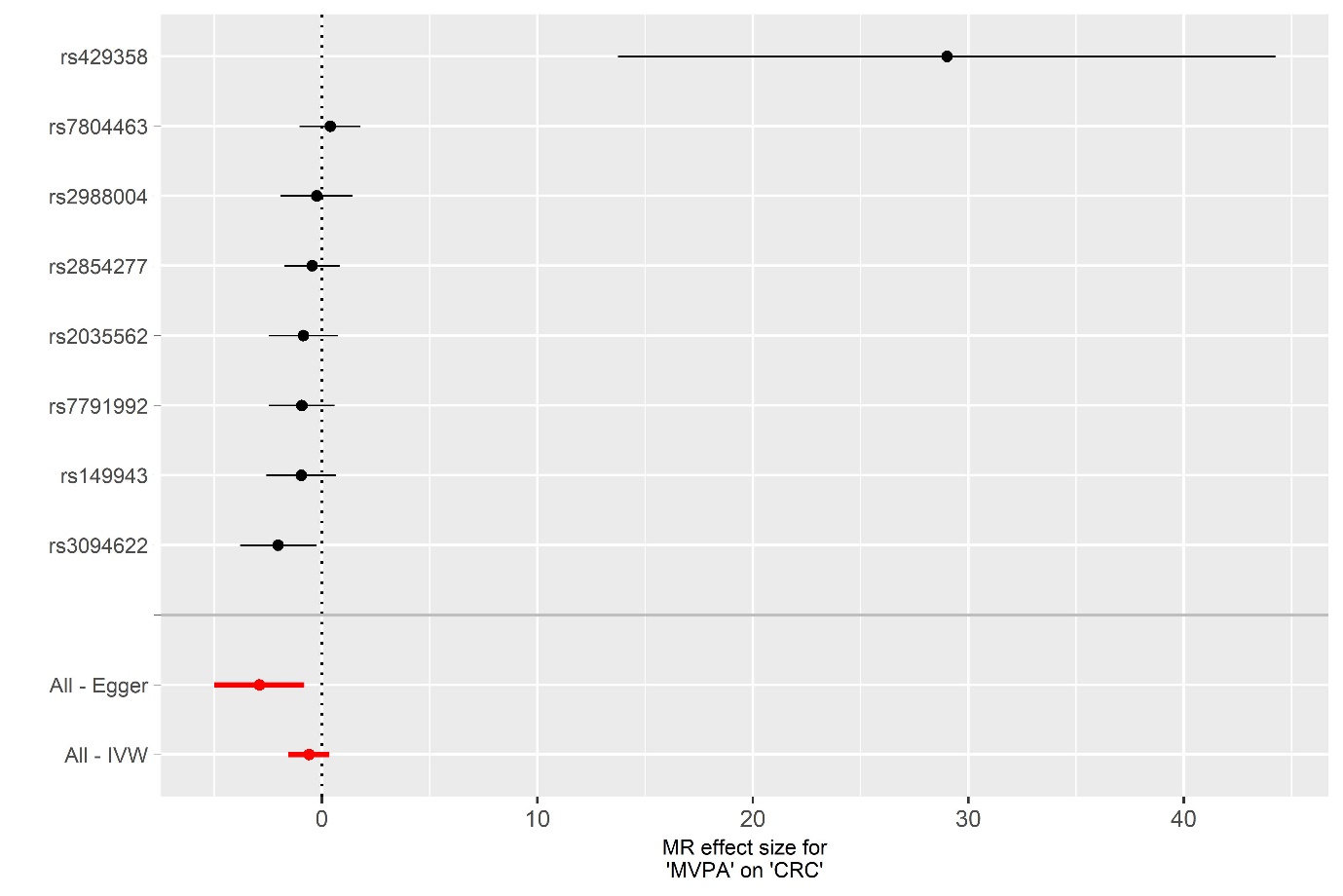
**

# Reference

1 Law PJ, Timofeeva M, Fernandez-Rozadilla C, Broderick P, Studd J, Fernandez-Tajes J, et al. Association analyses identify 31 new risk loci for colorectal cancer susceptibility. Nat Commun 2019; 10:2154.

2 Liu JZ, McRae AF, Nyholt DR, Medland SE, Wray NR, Brown KM, et al. A Versatile Gene-Based Test for Genome-wide Association Studies. The American Journal of Human Genetics 2010; 87:139-45.

3 Liu JZ, Tozzi F, Waterworth DM, Pillai SG, Muglia P, Middleton L, et al. Meta-analysis and imputation refines the association of 15q25 with smoking quantity. Nat Genet 2010; 42:436-40.

4 Klimentidis YC, Raichlen DA, Bea J, Garcia DO, Wineinger NE, Mandarino LJ, et al. Genome-wide association study of habitual physical activity in over 377,000 UK Biobank participants identifies multiple variants including CADM2 and APOE. Int J Obes (Lond) 2018; 42:1161-76.

5 Doherty A, Jackson D, Hammerla N, Plotz T, Olivier P, Granat MH, et al. Large Scale Population Assessment of Physical Activity Using Wrist Worn Accelerometers: The UK Biobank Study. PLoS One 2017; 12:e0169649.

6 Locke AE, Kahali B, Berndt SI, Justice AE, Pers TH, Day FR, et al. Genetic studies of body mass index yield new insights for obesity biology. Nature 2015; 518:197-206.

7 Bycroft C, Freeman C, Petkova D, Band G, Elliott LT, Sharp K, et al. The UK Biobank resource with deep phenotyping and genomic data. Nature 2018; 562:203-9.

8 Shungin D, Winkler TW, Croteau-Chonka DC, Ferreira T, Locke AE, Mägi R, et al. New genetic loci link adipose and insulin biology to body fat distribution. 2015; 518:187-96.

9 Rask-Andersen M, Karlsson T, Ek WE, Johansson A. Genome-wide association study of body fat distribution identifies adiposity loci and sex-specific genetic effects. Nat Commun 2019; 10:339.

10 Choi KW, Chen CY, Stein MB, Klimentidis YC, Wang MJ, Koenen KC, et al. Assessment of Bidirectional Relationships Between Physical Activity and Depression Among Adults A 2-Sample Mendelian Randomization Study. Jama Psychiatry 2019; 76:399-408.

11 Jia Y, Li F, Liu YF, Zhao JP, Leng MM, Chen L. Depression and cancer risk: a systematic review and meta-analysis. Public Health 2017; 149:138-48.

12 Relton CL, Davey Smith G. Two-step epigenetic Mendelian randomization: a strategy for establishing the causal role of epigenetic processes in pathways to disease. Int J Epidemiol 2012; 41:161-76.

13 Richmond RC, Hemani G, Tilling K, Davey Smith G, Relton CL. Challenges and novel approaches for investigating molecular mediation. Hum Mol Genet 2016; 25:R149-R56.

14 Carter AR, Sanderson E, Hammerton G, Richmond R, Smith GD, Heron J, et al. Mendelian randomisation for mediation analysis: current methods and challenges for implementation. BioRxiv 2019:835819.

15 Doherty A, Smith-Byrne K, Ferreira T, Holmes MV, Holmes C, Pulit SL, et al. GWAS identifies 14 loci for device-measured physical activity and sleep duration. Nat Commun 2018; 9:5257.
